# The Distant Siblings—A Phylogenomic Roadmap Illuminates the Origins of Extant Diversity in Fungal Aromatic Polyketide Biosynthesis

**DOI:** 10.1093/gbe/evv204

**Published:** 2015-11-03

**Authors:** Grzegorz Koczyk, Adam Dawidziuk, Delfina Popiel

**Affiliations:** ^1^ Department of Biometrics and Bioinformatics; ^2^ Department of Pathogen Genetics and Plant Resistance and Institute of Plant Genetics, Polish Academy of Sciences, Poznan, Poland

**Keywords:** polyketide, horizontal transfer, duplication, loss, secondary metabolism, sources of diversity

## Abstract

In recent years, the influx of newly sequenced fungal genomes has enabled sampling of secondary metabolite biosynthesis on an unprecedented scale. However, explanations of extant diversity which take into account both large-scale phylogeny reconstructions and knowledge gained from multiple genome projects are still lacking. We analyzed the evolutionary sources of genetic diversity in aromatic polyketide biosynthesis in over 100 model fungal genomes. By reconciling the history of over 400 nonreducing polyketide synthases (NR-PKSs) with corresponding species history, we demonstrate that extant fungal NR-PKSs are clades of distant siblings, originating from a burst of duplications in early *Pezizomycotina* and thinned by extensive losses. The capability of higher fungi to biosynthesize the simplest precursor molecule (orsellinic acid) is highlighted as an ancestral trait underlying biosynthesis of aromatic compounds. This base activity was modified during early evolution of filamentous fungi, toward divergent reaction schemes associated with biosynthesis of, for example, aflatoxins and fusarubins (C4–C9 cyclization) or various anthraquinone derivatives (C6–C11 cyclization). The functional plasticity is further shown to have been supplemented by modularization of domain architecture into discrete pieces (conserved splice junctions within product template domain), as well as tight linkage of key accessory enzyme families and divergence in employed transcriptional factors. Although the majority of discord between species and gene history is explained by ancient duplications, this landscape has been altered by more recent duplications, as well as multiple horizontal gene transfers. The 25 detected transfers include previously undescribed events leading to emergence of, for example, fusarubin biosynthesis in *Fusarium* genus. Both the underlying data and the results of present analysis (including alternative scenarios revealed by sampling multiple reconciliation optima) are maintained as a freely available web-based resource: http://cropnet.pl/metasites/sekmet/nrpks_2014.

## Introduction

The last 5 years of fungal genomics have been fruitful. We see mass characterization of fungal genomes, with increasing coverage given to both economically important and evolutionarily divergent lineages of eukaryotic microorganisms (Grigoriev et al. 2012; http://1000.fungalgenomes.org, last accessed May 30, 2015). Availability of next-generation sequencing methods sped up the pace of novel genomic projects, whereas the increased awareness and use of deletion libraries and heterologous expression systems allow characterization of larger sets of genes (e.g., Bergmann et al. 2007; Nielsen et al. 2011; Ahuja et al. 2012). However, our knowledge of evolutionary basis underlying the extant genetic diversity is still incomplete.

In particular, the origins of diversity in fungal polyketide synthesis have been the subject of intense investigations, even prior to the advent of whole-genome sequence analysis. Previously, the landmark paper by Kroken et al. (2003) has postulated that horizontal transfers are not required to explain major parts of observed variability in fungal iterative polyketide synthases. However, since then the increased sampling of genomic data from diverse taxonomic groups has enabled researchers from multiple groups to consider evidence in support of individual HGT (horizontal gene transfer) scenarios. Salient examples include the aflatoxin cluster (Slot and Rokas 2011), the fumonisin cluster (Khaldi and Wolfe 2011), as well as the ancient origin of partially reducing polyketide synthases from transfer originating in bacteria (Schmitt and Lumbsch 2009). Thus, the availability of data and advances in phylogenomics have permitted elucidation and study of individual scenarios contributing to the present-day diversity of fungal secondary metabolism. This “classic” approach to proposing and corroborating evolutionary scenarios typically (Schmitt and Lumbsch 2009; Khaldi and Wolfe 2011; Slot and Rokas 2011; Proctor et al. 2013) proceeds by thorough analysis of multiple individual reconstructions of gene histories in context of multiple reference species. The analysis is then followed by serial likelihood ratio tests aiming to partition the adjacent genes according to their associated phylogenies supporting one or more origin scenarios (e.g., duplication, horizontal transfer, and loss). The discussion of evolutionary events leading to extant diversity is thus limited to supporting the cases separately. Conversely, larger scale statistical modelling does not capture particular, individual events leading to known biosynthetic activities (e.g., CAFE analysis of gene family expansions/contractions in multiple lineages; Bie et al. 2006; Bushley and Turgeon 2010).

Consequently, what is still lacking are “phylogenetic roadmaps”—resources that strive to reconcile species and gene histories explicitly. These, by definition, will not provide a conclusive proof in favor of one or the other scenarios but can show the parts of family evolutionary history that are better explained with or without recourse to horizontal transfer versus duplication. A large scale inference followed by reconciliation can enumerate well-supported scenarios given different assumptions as to the cost of particular evolutionary events (i.e., transfer, duplication, loss). Based on maximum parsimony reconciliations we can identify monophyletic clades clearly dominated by speciation events (where orthologous relationships likely point to a conserved chemical structure of the base polyketide chain), as well as indicate potential transfers and pinpoint alternative donor–recipient scenarios. Fast reconciliation methods are now available (Doyon et al. 2011; Bansal et al. 2012), with scalable algorithms for efficiently sampling the space of optimal solutions at random (Bansal et al. 2013), as well as for finding the most likely scenario in a probabilistic framework (ALE, Amalgamated Likelihood Estimation; Szollosi, Rosikiewicz, et al. 2013).

Our work’s goal was to provide such a “proof-of-concept” roadmap for a large set of nonreducing polyketide synthases (NR-PKSs), involved in biosynthesis of diverse, well-characterized aromatic compounds (e.g., Cox 2007; Sanchez et al. 2012). This reconstruction and reconciliation was further supplemented by statistical analysis of genomic context and gene structure. For this, it was necessary to consider tradeoffs between scalability and accuracy of methods—choosing methods which produce best results in reasonable computation time (Anisimova et al. 2013) and provide a well-laid foundation for continuous inclusion of new (or corrected) data.

## Materials and Methods

**Fig. 1.—:**
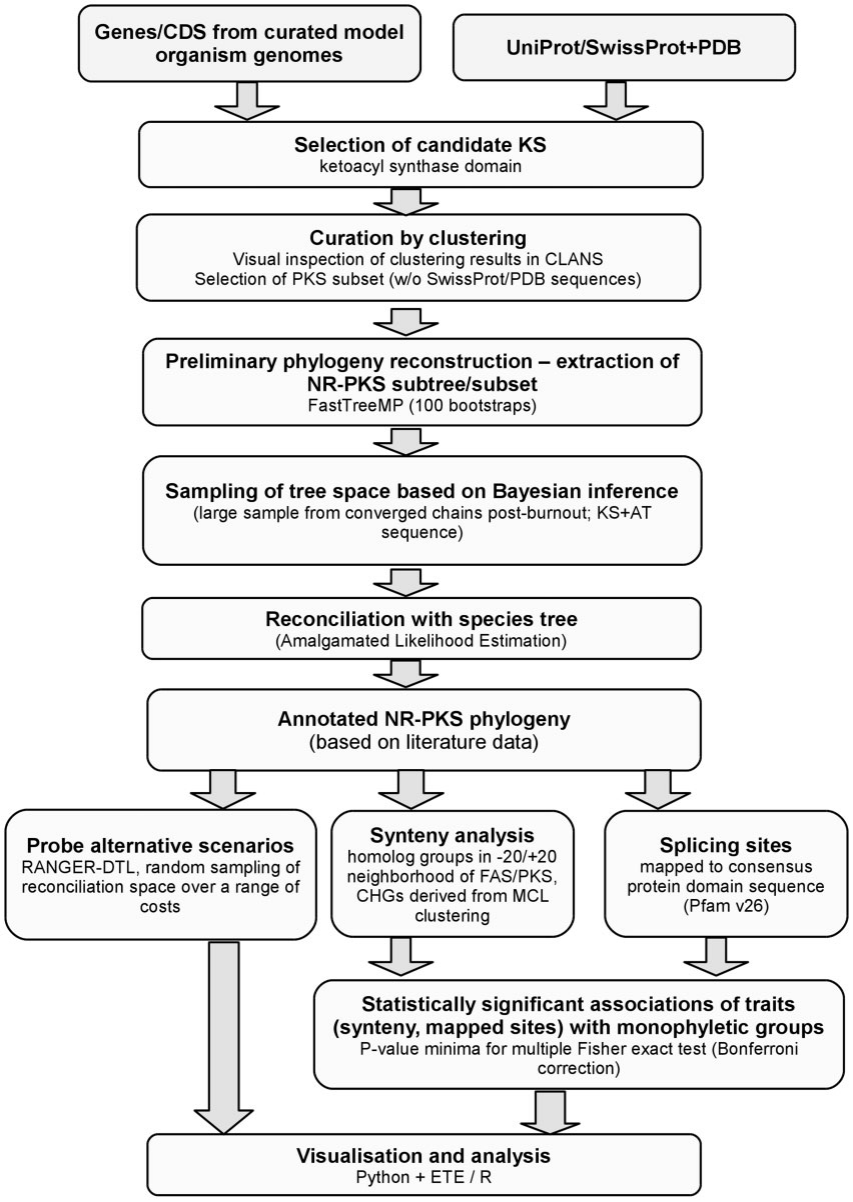
The workflow of phylogenomic analysis of NR-PKSs.

The entire workflow of conducted analysis is summarized on figure 1. Individual stages are described in detail below. All of the sequences and other supplementary material (i.e., trees, alignments, and annotations) referenced in this study can be found in the supplementary material, Supplementary Material online, as well as on the MetaSites database website, maintained by the authors: http://cropnet.pl/metasites/sekmet/nrpks_2014.

### Preparation of Reference Genomes

Genomes of 149 model fungi were gathered from the National Center for Biotechnology Information (NCBI)/GenBank (Benson et al. 2015), JGI-DOE/MycoCosm (Grigoriev et al. 2012), and Ensembl/Fungi (Flicek et al. 2014) repositories. This reference set has also included the PKS lacking genomes (mainly *Saccharomycetes* and *Schizosaccharomycetes*). The genome sequence of *Blumeria graminis* f. sp. *hordei* was taken from BluGen powder mildew genome database (http://www.blugen.org; Spanu et al. 2010, last accessed January 4, 2015). The complete list of genomes and data sources can be found in the supplementary table S1, Supplementary Material online. All of the reference genomes were curated to ensure that reading frames and gene structure matched the recorded protein translation for all protein-coding genes.

### Annotation of Domain Architecture

All protein-coding genes within the reference genomes were annotated with Hidden Markov Models, (HMMs) from Pfam database version 27 (Finn et al. 2014). Additionally, the mixed kingdom (Foerstner et al. 2008) and the fungi-specific (Delgado et al. 2012) models were created on basis of the alignments supplied with the original papers. These covered the typical NR-PKS domains (ketoacyl synthase, starter and main acyl transferase, thioesterase, methyltransferase, phosphopanthotein binding site, NAD-binding reductase). The HMM searches were conducted with the HMMer 3.0 hmmscan program (Eddy 2009) and were followed by double checking against the web-based version of NCBI Conserved Domain Database (Marchler-Bauer et al. 2015). The product template (PT) domain assignment was based solely on checking against hot dog fold superfamily signature in NCBI/CDD.

### Prescreening for NR-PKS Subset within Reference Genomes

The selection of full NR-PKS complements from model fungal genomes was supported by three independent criteria: The clustering of ketoacyl synthase sequences based on all versus all Basic Local Alignment Search Tool (BLAST) comparisons (pairwise similarities between individual sequences), the presence of architectural similarities (based on matching domain fingerprints), and inclusion in a monophyletic clade with all reference NR-PKS sequences during the preliminary phylogeny reconstruction step.

Initially, the set of ketoacyl synthases was selected from above-mentioned model genomes (including *Caenorhabditis elegans* as model outgroup), as well as PDB (Rose et al. 2013) and UniProt/SwissProt (UniProt Consortium 2014) sequences. The PDB (432 sequences) and SwissProt (611 sequences) subsets were included only during the clustering and initial selection of fungal PKS cluster, prior to preliminary phylogenetic tree reconstruction.

In the prescreening, only the sequences with at least one ketoacyl synthase domain occurrence (based on either fungal or mixed kingdom HMM matches, of length exceeding the threshold of 50 amino acids) were retained (3,067 in total). To further distinguish between the fragmentary sequences of incomplete PKSs (whether due to mistakes in gene prediction/annotation or due to evolutionary events) and the loosely associated unrelated ketoacyl thiolases with a partial KS domain signature, we conducted unsupervised clustering analysis with CLANS (Frickey and Lupas 2004). Clustering used a 10^−^^5^ inclusion threshold for hit *P* values. Based on membership of functionally and structurally annotated PKSs, the single cluster containing both *C. elegans* FAS and all reference PKS sequences (bacterial, fungal and protistan, 1,659 sequences) was chosen for further analysis.

To ensure the correct selection of the NR-PKS subset, we then employed an additional step based on the FastTree (Price et al. 2010; multithreaded version). One hundred bootstrap iterations were done on replicates obtained with SEQBOOT (Felsenstein 1989). This extended majority rule consensus tree was based solely on the ketoacyl synthase domain. MAFFT-LINSI v. 7.2 (Katoh and Standley 2013) was used to align the sequences, the resulting alignment was pruned with “trimal” (Capella-Gutiérrez et al. 2009) at 70% occupancy threshold for columns.

The final set of NR-PKS homologs was chosen by extracting the monophyletic fungal clade delineated by lowest common ancestor of all reference (experimentally validated) nonreducing polyketide synthases (node with full support from bootstrap). The 414 representatives from model fungal genomes (discarding PDB and SwissProt sequences, but including the fumonisin HR-PKS outgroup sequence) were retained for gene tree inference based on presence of conserved KS-AT module.

No outliers or additional sequences were indicated by either phylogeny or domain architecture, save for *Ustilago maydis PKS1*/*UM04105* (and its two counterparts from *Ustilago hordei* and *Sporisorium reilianum*) which lacks an acyl transferase domain and thus was not included in the final analysis. For a single experimentally characterized NR-PKS *dbaI* (*ANIA_07903*; Gerke et al. 2012), the sequence was manually updated to match revised AspGD gene model which translates to a full length PKS protein instead of the truncated version.

### Gene Tree Construction

The final NR-PKS alignment was constructed by aligning the core KS-AT module with MAFFT-LINSI (Katoh and Standley 2013). This alignment was curated with T-COFFEE transitive consistency score analysis (Chang et al. 2014; default exhaustive “proba_pair” setting—columns scoring 2 or above were kept in the final alignment, in keeping with the results of the above-mentioned article). The filtered alignment (available as supplementary material S2, Supplementary Material online, in NEXUS format; mappings of sequence identifiers to loci/gene names are available as additional supplementary file S3, Supplementary Material online) was used for final phylogeny reconstruction.

As the protein sequence identity levels can be low and homoplasies are expected in the data set, Bayesian inference (BI) was conducted with PhyloBayes-MPI (Lartillot et al. 2013; 4 chains 200,000 each, first 40,000 trees discarded as burn-in, every fifth tree sampled; two chains of best convergence chosen for further analysis; CAT-Poisson parameterization was used due to computational resource constraints). Chain convergence was assessed with pairwise comparison using “bpcomp” tool (Lartillot et al. 2009). The analysis was also carried out in maximum-likelihood (ML) framework with IQTREE 0.9.6 (Minh et al. 2013; 1,000 ultrafast bootstrap replicates; exhaustive nearest neighbor search setting “-nni5”; LG+G4+I+F model selected with IQTREE internal model testing). The Bayesian consensus tree and the underlying sampled trees were selected for reconciliation analysis with ALEml and DTL-RANGER; the resulting ALEml tree is hereafter referred to as “amalgamated.” Reconstructed gene tree topologies are available in supplementary material, Supplementary Material online (ML, Bayesian, and amalgamated in supplementary files S4–S6, Supplementary Material online). The amalgamated tree with annotated gene structure and domain architecture is shown in supplementary figure S1, Supplementary Material online.

Throughout the text, we refer to the nodes of the amalgamated gene tree by the “g<number>” notation derived from postorder traversal of tree structure. Where the original BI tree or the species tree is referenced, the analogous “go<number>” or “so<number>” notations are used.

### Species Tree Construction

The species tree was constructed based on 23 best scoring single-copy orthologs (see supplementary table S2, Supplementary Material online) with best topological scores (over 95%) as reported in FUNYBASE (Marthey et al. 2008). The topological scores were previously introduced by Aguileta et al. (2008) and measured concordance of ML trees based on individual orthologs with a reference supertree inferred on basis of over 120 single copy orthologs identified in that study. The ortholog protein sequences were aligned with MAFFT-LINSI and resulting alignments concatenated (the concatenated alignment is available in the supplementary file S7, Supplementary Material online, in NEXUS format). The gapped positions were curated using 70% occupancy threshold (-gt 0.7) in “trimal,” resulting in 15,314 columns in the final alignment (available as supplementary file S2, Supplementary Material online). BI was conducted with PhyloBayes-MPI 1.5a (Lartillot et al. 2013). The inference consisted of 4 chains of 40,000 iterations, first 5,000 trees from each chain were discarded as burn-in, every fifth tree was sampled. As in the case of gene tree reconstruction, two chains of best convergence were chosen for further analysis, CAT-Poisson parameterization was used due to computational resource constraints.

Additional ML reconstruction was conducted with IQTREE. Individual protein models were chosen for each of the 23 genes based on ProtTest v3 (Darriba et al. 2011) results (corroborated based on IQTREE’s own model testing). The reconstruction used ultrafast bootstrap with default stopping criterion and exhaustive NNI (Nearest Neighbor Interchange) search (-nni5 option), resulting in convergence after 100 ultrafast bootstrap iterations. *Caenorhabditis elegans* represented the outgroup used to root the tree.

To obtain the approximate chronogram, the dating was performed on PhyloBayes-MPI consensus tree. Relaxed, log-normal autocorrelated clock with soft bounds under a birth–death prior was used (as implemented in PhyloBayes 3.3f; Lartillot et al. 2009). The dating constraints were introduced based on *Fungi*/*Animalia* split dated at 983 Ma (based on Douzery et al. 2004), as well as number of additional constraints based on subsequent inquiries into fungal phylogeny (Sung et al. 2008; Gueidan et al. 2011; Ohm et al. 2012; O’Donnell et al. 2013). All dating constraints are summarized in supplementary table S3, Supplementary Material online. The Bayesian and ML species trees are shown on supplementary figures S2 and Supplementary Data, Supplementary Material online, the underlying topologies are also available in supplementary materials, Supplementary Material online, as Newick format files (Bayesian consensus tree—supplementary file S8, chronogram—supplementary file S9, and ML consensus tree—supplementary file S10, Supplementary Material online).

### Tree Reconciliation

The resulting chronogram (dated species tree) and the ensemble of Bayesian gene trees were reconciled using the ALE approach, as implemented in ALE v.0.3 (40,000 trees discarded as burnin, every fifth tree sampled from both runs). To minimize numerical errors on the large data set, scaled version of the program was compiled with floating point calculations carried out on 128 bit numbers (as implemented by “boost::multiprecision” library “float128” type). The inferred transfers are summarized in table 1.

**Table 1:** Summary of Transfers Predicted by ALE and the Consistency of Their Support Based on DTL-RANGER Sampling

Clade	No.	Donor Gene Tree Node^a^	Top Transfer Cost (at *L* = 1)^b^	Consistent^c^	Description of Affected Genes
β (azaphilones, meroterpenoids)	1	g109 (go109)	15 (15)	**+**	Origin of *O9MDRAFT_11331* in *Hysterium pulicare* (s212) as a transfer from the lineage of early-diverging leotiomycete *Glarea lozoyensis* (s135)
	2	g110 (go110)	12 (12)	**+**	Origin of *ACLA_061390* in *Aspergillus clavatus* (s264) as a transfer from the lineage of *Glarea lozoyensis* (s135)
	3	g166 (go167)	22 (10)		Origin of *TSTA_060720* (possible meroterpenoid biosynthesis gene) in *Talaromyces stipitatus* (s261), as a transfer from the ancestral lineage of *Thielavia terrestris* (s155)
	4	g229 (go226)	27 (12)	**+**	Origin of *Pc21g05070* in *Penicillium rubens* (s263), as a transfer from the ancestral lineage of *Colletotrichum graminicola* (s165)^d^. The affected gene is the sorbicillinoid biosynthetic NR-PKS (*sorbB*)
γ.C2–C7a monocyclic (orsellinic acid, resorcylic acid lactones)	5	g352 (go352)	12 (11)		Origin of *TRIATDRAFT_188840* in *Trichoderma atroviride* (s182), as a transfer from the donor lineage of *Talaromyces stipitatus* (s261)
	6	g355 (go372)	12 (11)		Origin of *MBM_06725* in *Marssonina brunea* (s137), as a transfer from the donor lineage of *Talaromyces marneffei* (s260)
	7	g361 (go367)	15 (15)	**+**	Origin of *ANIA_07909* (*orsA* gene) in *Aspergillus nidulans* (s271), as a transfer from the donor lineage of *Glarea lozoyensis* (s135)
	8	g362 (go368)	12 (11)		Origin of *O9CDRAFT_102749* in *Sphaerulina populicola,* as a transfer from the donor lineage of *Glarea lozoyensis* (s135) to the common ancestor of *Sphaerulina populicola* and *S. musiva* (s207)
	9	g369 (NA)	12 (NA)		Origin of *SETTUDRAFT_152662* in *Setosphaeria turcica* (s220), as transfer from the donor lineage of *Talaromyces marneffei* (s260)
	10	g370 (go359)	12 (11)		Origin of *PMAA_061720* in *Talaromyces marneffei* (s260), as transfer from the donor lineage of *Trichoderma* sp. (s186)
	11	g371 (go360)	12 (11)		Origin of *CHGG_08141* in *Chaetomium globosum* (s157), as a transfer from the donor lineage of *Trichoderma* sp. (s186)
	12	g383 (go381)	14 (13)	**+**	Origins of *LEMA_P09870* and *SETTUDRAFT_161587*, as transfer from the ancestor of *Colletotrichum higginsianum* (s164) to the acceptor lineage within *Pleosporales* (s228), postdivergence of *Stagonospora nodorum*)
γ.C6–C11 (emodins, atrochyrsone, asperthecin)	13	g418 (go419)	17 (10)		Origin of *BC1G_08227* in *Botrytis cinerea* (s136), as transfer from the common ancestor of *Pleosporales* (s229)
	14	g454 (go510)	7 (8)		Origin of *pkgA* orthologs (alternariol biosynthetic NR-PKS) in *Aspergillus nidulans*, *A. flavus* and *A. oryzae*. Donor is predicted to be the common ancestor of analyzed *Sordariomycetes* (s197), acceptor is the ancestral lineage of *Aspergillus* sp. sections *nidulantes, nigri,* and *flavi* (s283)
	15	g462 (go517)	17 (13)	**+**	Origin of *COCHEDRAFT_1108855*, *COCHEC4DRAFT_28198*. The genes are homologs of asperthecin biosynthetic NR-PKS (aptA). Transfer from the ancestral lineage of *Colletotrichum higginsianum* (s164) to the common ancestor of both model *Bipolaris maydis* strains (s224)
	16	g491 (go547)	12 (12)		Origin of *MAA_08920*, *MAA_04116*. The genes are homologs of neosartoricin biosynthetic NR-PKS (NscA). Transfer is indicated from the common ancestor of *Aspergillus* sp. section *fumigati* (s269). Acceptor is the common ancestor of *Metarhizium* sp. (s189)
	17	g490 (go546)	12 (12)	**+**	Origin of *MCYG_03598*, *MGYG_06588*, *TESG_06702*, *TERG_08357*, *TRV_00386*, *ARB_00538*. The genes are homologs of neosartoricin biosynthetic NR-PKS (*nscA*). Transfer from the common ancestor of *Aspergillus* sp. section *fumigati* (s269). Acceptor is the common ancestor of *Arthroderma* (*Trichophyton*) sp. (s257)
	18	g511 (go433)	21 (15)	**+**	Origin of *NFIA_101660* (distant homolog of *ACAS*) as transfer from the direct ancestor of *Trichoderma atroviride* (s182) to the direct ancestor of *Neosartorya fischerii* (s265)
	19	g542 (go468)	29 (20)	**+**	Origin of *MAA_06575* (distant homolog of *ACAS*) as transfer from the common ancestor of *Pleosporaceae* (s227) to the direct ancestor of *Metarhizium robertsii* (s187)
γ.C2–C7c multicyclic (naphtopyrone, melanins, aurofusarin, bikaverin)	20	g664 (go754)	>40 (>40)	**+**	Origin of *ATEG_07500* (previously described as putative *pksP* homolog; shown to be a xenolog of *pksN* pigment biosynthetic PKS from *Fusarium solani*). Transfer is indicated from the donor lineage of *Metarhizium anisopliae* (s181) to the ancestor of *Aspergillus terreus* (s272)
γ.C4–C9 (aflatoxins, sterigmatocystin, fusarubins)	21	g731 (go684)	15 (16)	**+**	Origin of *THITE_44861* as transfer from the common ancestral line of *Aspergillus* sp. (s275) to ancestor of *Thielavia terrestris* (s155). The gene belongs to the sister clade of fusarubin and aflatoxin NR-PKSs
	22	g740 (go647)	14 (NA)		Origin of *MGG_04208*, *GLRG_08620*, *CH063_02506* genes in *Sordariomycetes* (s197) line as transfer from the common ancestor of *Dothideomycetideae* (s211). The genes belong to the sister clade of fusarubin and aflatoxin NR-PKSs
	23	g766 (go667)	26 (27)	**+**	Transfer of sterigmatocystin biosynthetic cluster to *Podospora anserina* (s153) lineage, from the ancestral lineage of *Rhytidhysteron rufulum* (s213)
	24	g791 (go704)	17 (16)	**+**	Origin of *GLRG_11956* (distant homolog of *fsr1*) as transfer from the ancestral lineage of *Rhytidhysteron rufulum* (s213). Acceptor is the ancestor of *Colletotrichum graminicola* (s165)
	25	g807 (go719)	17 (16)	**+**	Transfer of *fsr1* (fusarubin biosynthesis NR-PKS) from common ancestor of *Pleosporales* (s229) to common ancestor of *Fusarium* sp. (s181)

^a^First number denotes node in gene tree post-ALE reconciliation; if bipartition is present in original BI tree, the number is given in parentheses.

^b^Transfer cost at which transfer event is predicted for majority of sampled scenarios; second number given in parentheses, if bipartition is present in the original BI tree.

^c^Most robust predictions where detected transfer is shown across multiple transfer cost thresholds (for *L* = 1, 2, 3; on both the amalgamated and the original gene tree) are indicated with plus sign.

^d^In nonamalgamated tree, the different parent bipartition results in two inferred transfers at *T* = 12.0 (second involves TRIREDRAFT_73621).

Additional support for transfers as well as alternative scenarios was explored by sampling the multiple optimal reconciliations with DTL-RANGER (Bansal et al. 2012). Here, the support for duplication/speciation/transfer events as well as mapping of individual events on the species tree was annotated on basis of 1) frequency of inferred events/mappings across different transfer cost values and 2) highest transfer cost where transfer event is indicated (only for inferred HGT events), with fixed duplication and loss costs (DTL-RANGER dated version with parameters *L* = {1, 2, 3}, Δ = 4, θϵ{5, . . . , 40}). The rationale here is that the horizontal transfer is best supported where “regrafts” between cotemporaneous parts of species tree are both most evident (appear at highly penalized transfer cost) and most consistent (as supported for a range of decreasing cost thresholds). For the purpose of sampling, the reconciliations at random, the algorithms from Bansal et al. (2013) were used with a random sample size of 1,000 optimal solutions per each cost combination.

### Synteny Analysis

To establish the enrichment or depletion of specific gene families/subfamilies in the syntenic context of NR-PKS core genes, we introduced an approach based on multiple applications of the Fisher exact test.

First, all homologs within +20/−20 genomic context were extracted and subjected to exhaustive, all against all BLASTP searches. The BLASTP expectation values were then used for clustering with MCL (Enright et al. 2002). Only (bidirectional) BLAST hits with *E* value less than 1E-10 and majority coverage (>50%) of the longer sequence were considered. The MCL inflation threshold was set to 1.4. The clustering parameters were chosen as a compromise between stringent clustering (*E* value) and observed property of high inflation thresholds erroneously breaking up the clusters for highly diverse sequences (Chan et al. 2013). For our analysis, the inflation threshold setting was also supported in the average values of silhouette width (Rousseeuw 1987), a cluster quality measure independent of predefined class labels which consistently presented a slight peak at the 1.4 setting.

The 2×2 contingency tables were set up to contrast inclusion of NR-PKS gene in a given clade (vs. the rest of the tree), with presence/absence of a candidate homolog group member in the vicinity of the said gene. Thus, the resulting candidate homolog groups numbering ten or more sequences (133 clusters) were iteratively tested for association with all possible subtrees containing five or more leaves. As remarked above, this was done with Fisher exact test, corrected for multiple testing with Bonferroni correction (based on total number of tests for all candidate homolog groups). The local *P*-value minima were marked and test results corresponding to the most significant, nonoverlapping subtrees reported for each homologous group. The corrected *P*-value threshold for inclusion in reported results was set to 0.0001. The detected associations are summarized in table 2 and an example demonstrating the detection of one of the strongest associations (β-lactamase accessory enzymes) is included in supplementary figure S4, Supplementary Material online.

**Table 2:** Summary of Strongest Associations between Syntenic Homologs Present in the Genomic Neighborhood (of the core NR-PKS) and Monophyletic Clades of NR-PKSs

Clade	Gnode	#	CHG	Description—CHG (Candidate Homolog Group)	*P* Value^a^	Example Accessory Genes
β (azaphilones, meroterpenoids)	*g184*	1	ch109	Terpene cyclase	4E-06	*trt1—Aspergillus terreus* terretonin biosynthesis terpene cyclase, *ausL—A. nidulans* austinol biosynthesis terpene cyclase
	*g204*	2	ch71	Mitochondrial carrier protein (Pfam: Mito_carr)	3E-05	—
	*g273*	3	ch73	YCII superfamily protein (Pfam: YCII)	8E-07	*dbaC*—implicated in biosynthesis of DHMBA (dihydroxy-3-methyl-6-(2-oxopropyl)benzaldehyde) in *A. nidulans*
	*g332*	4	ch7	Accessory HR-PKS	1E-06	*zea2—Fusarium graminearum* zearalenone biosynthetic HR-PKS, *afoG—A. nidulans* asperfuranone biosynthetic HR-PKS
	*g335*	5	ch9	FAD-binding oxidoreductase	3E-25	*tropB—Talaromyces stipitatus* tropolone biosynthesis FAD monooxygenase, *afoD—A. nidulans* asperfuranone biosynthesis FAD-dependent oxidase
		6	ch12	Zinc-binding oxidoreductase (alcohol dehydrogenase)	1E-06	*fsr4—F. fujikuroi* fusarubin cluster oxidoreductase^b^, *PMAA_101580*—*T. marneffei* mitorubrinol biosynthesis oxidoreductase
		7	ch48	Acetyltransferase	2E-09	*PMAA_101560*—*T. marneffei* mitorubrinol biosynthesis acyltransferase
	*g336*	8	ch20	FSH1 (serine hydrolase)	4E-19	*afoC—A. nidulans* asperfuranone biosynthesis oxidoreductase, *dbaE—A. nidulans* DHMBA biosynthesis esterase/lipase
		9	ch26	2-oxoglutarate/Fe(II)-dependent dioxygenase	7E-11	*tropC—T. stipitatus* nonheme iron(II)-dependent dioxygenase involved in tropolone biosynthesis, *encD—A. fumigatus* dioxygenase involved in endocrocin processing
	*g337*	10	ch17	Zinc-finger transcription factor	3E-24	*rp1—T. marneffei* transcriptional activator (red pigment/citrinin cluster), *dbaA—A. nidulans* DHMBA biosynthesis TF
γ.C2–C7a monocyclic (orsellinic acid, resorcylic acid lactones)	*g405*	11	ch7	Accessory HR-PKS	3E-08	*zea2—F. graminearum* zearalenone biosynthetic HR-PKS, *afoG—A. nidulans* asperfuranone biosynthetic HR-PKS
γ.C6–C11 (emodins, atrochrysone, asperthecin)	*g454*	12	ch46	Short-chain dehydrogenase (classical SDR subtype)	3E-05	*mppA—Monascus pilosus* azaphilone biosynthesis oxidoreductase^b^
	*g490*	13	ch68	Policyclic prenyltransferase (pcPTase)	6E-10	*dmaT*/*cpaD—A. flavus* cyclopiazonic acid biosynthetic tryptophan dimethylalliltransferase, *nscD—Neosartorya fischerii* pcPTase involved in neosartoricin biosynthesis
	*g492*	14	ch23	Zinc-finger transcription factor	8E-11	*aurR2—F. graminearum* aurofusarin biosynthetic TF, *nscR—N. fischerii* neosartoricin biosynthetic TF
	*g493*	15	ch60	FAD-binding oxidase (Pfam: FAD_binding_3)	1E-22	*aptC—A. nidulans* asperthecin biosynthetic monooxygenase, *nscC—N. fischerii* neosartoricin biosynthetic monooxygenase
	*g494*	16	ch32	FAD-binding oxidase (Pfam: FAD_binding_4)	8E-07	—
	*g548*	17	ch36	Scytalone dehydratase	5E-08	*arp1—N. fischerii* conidial pigment dehydratase, *mdpB—A. nidulans* dehydratase required for prenylated xanthones' biosynthesis
	*g549*	18	ch18	Dehydratase (EthD domain)	1E-09	*aurZ—F. graminearum* aurofusarin dehydratase
		19	ch51	Domain of unknown function (Pfam: DUF1772)	1E-05	*aflCa*/*hypC—A. flavus* noranthrone monooxygenase, *encC—A. fumigatus* endocrocin biosynthetic monooxygenase
		20	ch131	Methyltransferase	5E-09	*gedD—A. terreus* geodin biosynthesis methyltransferase
	*g550*	21	ch31	NAD-binding oxidase (Pfam: NAD_binding_10)	5E-22	*mdpK—A. nidulans* oxidoreductase required for prenylated xanthones' biosynthesis, *aflI/avfA/stcO—A. flavus* averufin oxidase
		22	ch41	Baeyer–Villiger oxidase (Pfam: DUF4243)	3E-07	*mdpL—A. nidulans* oxidase required for prenylated xanthones’ biosynthesis, *gedK—A. terreus* geodin biosynthesis oxidase
		23	ch43	Zinc-finger transcription factor (Pfam: AflR)	5E-09	*aflR—A. flavus* aflatoxin biosynthesis transcription factor, *mdpE—A. nidulans* monodictyphenone biosynthesis transcription factor
	*g552*	24	ch14	β-lactamase	3E-42	*gedB/ACTE—A. terreus* atrochrysone/geodin biosynthesis thioesterase, *aptB—A. nidulans* asperthecin biosynthesis thioesterase
γ.C2–C7b bicyclic (THN, melanins)	*g587*	25	ch81	Prefoldin (chaperone)	4E-19	— (housekeeping gene)
	*g623*	26	ch125	Pre-mRNA-splicing factor *prp1/6* homolog (proteasome component)	8E-09	— (housekeeping gene)
	*g624*	27	ch94	Imide hydrolase (Pfam: Hydantoinase)	9E-08	—
	*g627*	28	ch33	Zinc-finger transcription factor	1E-29	—
γ.C2–C7c multicyclic (naphtopyrone, melanins, aurofusarin, bikaverin)	*g669*	29	ch130	Maleylacetate reductase homolog (iron-containing alcohol dehydrogenase)	2E-10	—
		30	ch132	Zinc-finger transcription factor	7E-08	—
	*g670*	31	ch37	FAD-binding oxidase (Pfam: FAD_binding_3)	1E-06	*dbaB—A. nidulans* FAD-binding monooxygenase implicated in DHMBA biosynthesis
	*g696*	32	ch101	Multidomain protein (multiple zinc-finger sites, jumonji, ARID domains)—distant homology to histone demethylases (*JARID1C*; 27% sequence identity)	4E-09	—
		33	ch116	GIY-YIG nuclease superfamily protein (structure-specific endonuclease subunit *slx1* ortholog)	2E-07	— (housekeeping gene)
		34	ch126	Glycosyl hydrolase family 31 proteins (∼50% sequence identity to model α-glycoside hydrolases)	2E-10	—
γ.C4–C9 (aflatoxins, sterigmatocystin, fusarubins)	*g772*	35	ch43	Zinc-finger transcription factors (Pfam: AflR)	3E-06	*aflR—A. flavus* aflatoxin biosynthesis transcription factor, *mdpE—A. nidulans* prenylated xanthone biosynthesis TF
		36	ch49	Accessory FAS—type α	8E-09	*aflA/fas-2/stcJ—A. flavus* fatty acid synthase α (aflatoxin biosynthesis), *rp3—Talaromyces marneffei* red pigment biosynthesis 3-oxoACP reductase
		37	ch54	Accessory FAS—type β	1E-07	*aflB/fas-1/stcK—A. flavus* fatty acid synthase β (aflatoxin biosynthesis), *rp2—T. marneffei* red pigment biosynthesis fatty acid synthase β
	*g787*	38	ch58	3-keto-ACP reductase (classical SDR)	6E-05	—
		39	ch85	Flavin-binding monooxygenase (Pfam: FMO_like)	6E-06	*aurF—F. graminearum* rubrofusarin monooxygenase (aurofusarin biosynthesis)
	*g813*	40	ch1	Methyltransferase	7E-05	*aflO* and *aflP—A. flavus* methyltransferases involved in aflatoxin biosynthesis, *fsr2—F. fujikuroi* fusarubin biosynthesis methyltransferase^b^
γ.C2–C7b/c + γ.C4–C9	*g817*	41	ch6	Laccase (Cu-dependent oxidoreductase)	1E-06	*abr2—A. fumigatus* laccase required for oxidative polymerization of DHN, *gip1—F. graminearum* laccase dimerizing two 9-hydroxyrubrofusarin molecules (aurofusarin biosynthesis)
γ.C6–C11 + γ.C2–C7b/c + γ.C4–C9	*g818*	42	ch8	Enoyl-CoA reductase	2E-07	*ver1*/*aflM—A. flavus* versicolorin reductase (aflatoxin biosynthesis), *3HNR* and *4HNR—Magnaporthe grisea* trihydroxynaphtalene and tetrahydroxynaphtalene reductase (melanin biosynthesis)^b^, *mdpC—A. nidulans* reductase involved in biosynthesis of monodictyphenone and xanthones

^a^Post-Bonferroni correction.

^b^Model accessory genes from species not covered by the phylogeny reconstruction.

An analogous approach was employed for the gene structure, where exon positions were mapped to the respective protein domain sequence alignments and counts calculated for consensus positions based on the said alignments. The starter acyltransferase (SAT), ketoacyl synthase (KS), main acyltransferase (AT), and PT domains were analyzed based on the realignment (MAFFT-LINSI) of corresponding sequence fragments detected earlier. Due to highly divergent sequences of C-terminal domains (methyltransferase, thioesterase, NAD-dependent reductase) and the apparent incompleteness of a fraction of gene models, these were excluded from splicing site analysis.

### Visualization

The trees/dendrograms and annotation (gene structure, domain architecture, reconciled events) were visualized using custom Python scripts dependent on BioPython (Cock et al. 2009; Talevich et al. 2012) and ETE2 (Huerta-Cepas et al. 2010). Where final, annotated versions of published genomes had yet to appear in NCBI/GenBank, annotated gene names were derived following the practice of combining existing gene numbers with organism-specific prefix (locus prefix tag) based on codes available through genome sequencing project pages (http://www.ncbi.nlm.nih.gov/bioproject/, last accessed January 4, 2015).

The functional annotation for experimentally verified and described nonreducing polyketide synthases was gathered from available scientific literature manually (for complete list and descriptions, see supplementary table S4, Supplementary Material online). Classification of cyclization activities (C2–C7, C6–C11, C4–C9) originates from nomenclature used by Li et al. (2010). The structural model of PT domain was visualized with PyMol 1.7.2 (www.pymol.org, last accessed May 30, 2015).

## Results and Discussion

### Inferred History of NR-PKS Complement Is Strongly Supported

**Fig. 2.—:**
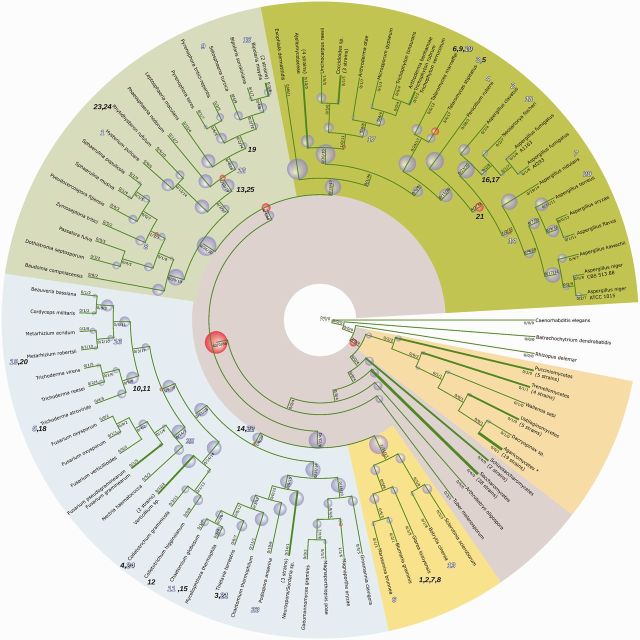
The ancestral duplications, subsequent transfers, and gene losses in the NR-PKS complement—annotated on the simplified species tree of higher fungi. The events were annotated based on the results of ALE reconciliation. Deletions are indicated by violet bubbles, duplications by red bubbles. Transfer events are marked through their respective numbers from table 1 (filled outlines indicate donors, hollow outlines indicate transfer acceptors). Salient broad taxa within higher fungi are highlighted by colored backgrounds (in clockwise direction: *Basidiomycota*, *Leotiomycetes*, *Sordariomycetes*, *Dothideomycetes*, *Eurotiomycetes*). Subscript on branches denotes predicted numbers of, respectively, duplications/deletions/genes for respective branch. For ease of reference, some nodes were collapsed (thickened branches, proportional to the number of species/strains). Duplications within the *Agaricomycetes* clade are not shown due to space constraints (see fig. 4).

The reconstructed species tree (see fig. 2 for simplified representation showing predicted evolutionary events), based on 23 single copy orthologous genes, is largely in accord with previously published results, in particular the comprehensive phylogenomic analysis by Ebersberger et al. (2012) and alignment-free composition vector-based comparison by Wang et al. (2009). Notably, the set of selected orthologs supports the alliance of *Dothideomycetes* and *Eurotiomycetes* postulated by both. The individual splits are well supported by both inference methods (BI and ML), with majority of splits within Bayesian consensus tree supported by both methods (see supplementary material, Supplementary Material online).

**Fig. 3.—:**
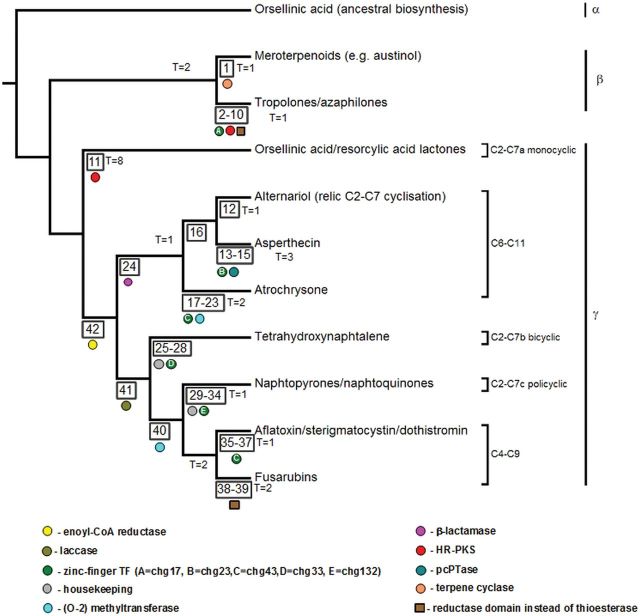
The divergence of major NR-PKS clades, which enabled diversification of biosynthesized aromatic compounds, was facilitated by close linkage with key accessory enzymes and transcriptional factors. The simplified phylogeny (based on amalgamated gene tree) shows selected groups of syntenic homologs as colored shapes (also indicated by numbers in box shape, which reference descriptions in table 2).

The reconstruction of NR-PKS phylogeny (the gene tree) was based on the conserved KS-AT fragment of the megasynthases. Again, majority of splits are well supported by both Bayesian and ML results. Notably, although approximately 15% of splits (60/413) present in the Bayesian consensus tree are challenged during ALE reconciliation, the subdivision into major clades (as depicted on fig. 3) is not modified.

### Inferred Events Are Largely Supported by Both Reconciliation Methods

Within the limits of comparability, the predictions obtained with DTL-RANGER and ALE align well (see supplementary tables S5–S7, Supplementary Material online, for details). Expectedly, there is a marked decrease of reconciliation cost on the amalgamated topology (which resolves uncertainties in the original Bayesian consensus in favor of the species tree) with reported minimum costs on average 10% lower, across all investigated parameter combinations (see supplementary table S5, Supplementary Material online, for detailed comparison).

Based on the amalgamated topology (where direct comparison is possible) predicted duplications and speciations are largely the same for DTL-RANGER (across varying cost thresholds). In particular, for the following parameter combinations there is over 99% identity in predictions (costs given in <D, T, L> format for, respectively, duplication, transfer, and loss): <4, 10–12, 1>, <4, 18–20, 2>, and <4, 25–28, 3>. Analogous results were observed for mapping of the events to the corresponding nodes of the species tree for the above-mentioned parameter values. Overall, although predictions for speciation and duplication nodes correspond to ALE results in overwhelming majority (peaking at 98-99%), this correspondence is worse for transfer predictions (peaking at 92%, considerably worse for many parameter values). The DTL-RANGER predictions on the original tree (compared over the 352 bipartitions shared between the original Bayesian consensus and the amalgamated topology) also align well, showing that simple reconciliation without rearrangement can capture at least part of the events correctly (see supplementary tables S6 and Supplementary Data, Supplementary Material online, for details).

Pertinently, both the inferred existence and the tentative topological dating of majority of duplication events (∼70%) are unperturbed regardless of the assumed parameters. Thus, our prediction of the ancestral nature of most underlying duplications is largely independent of detected transfers.

### Biosynthesis of Orsellinic Acid Is an Ancestral Trait of Higher Fungi

**Fig. 4.—:**
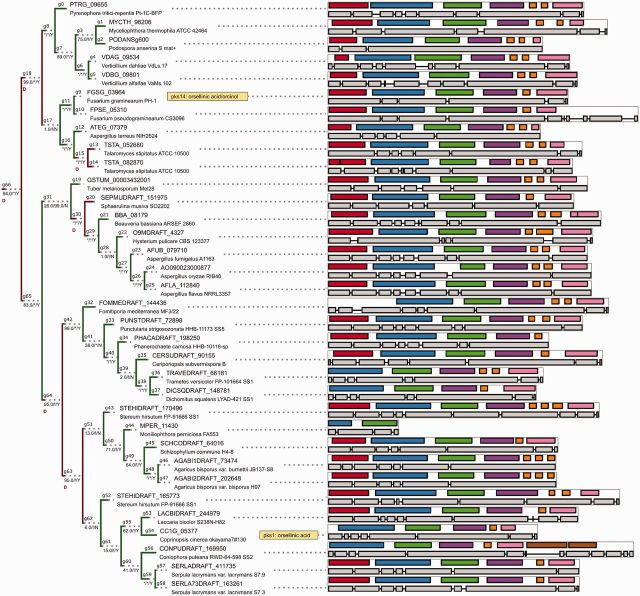
The phylogeny of ancestral clade α, which predates basidiomycete–ascomycete split, contains conserved NR-PKSs biosynthesizing orsellinic acid (*pks14*, *pks1*). Support noted below branches in form BI/ML/C (BI, support from BI; ML, support from ML ultrafast bootstrap; C, whether bipartition is present in Bayesian consensus tree; * denotes full support, % denotes support below 1% level). Domain architecture and gene structure are visualized on the right side (colors: red, SAT; blue, KS; green, AT; purple, PT; orange, ACP; pink, TE; brown, R). Color of the tree edges and letters denote predicted events (D, duplication; TD, transfer donated; TA, transfer accepted). Tree is not drawn to scale.

The obtained results support ancient origins of biosynthesis of compounds derived from modification of one or more molecules of orsellinic acid, in all three ancestral clades (α, β, and γ). The earliest diverging clade α (see figs. 3 and 4) encompasses both basidiomycete and ascomycete sequences, including ones with experimentally established propensity toward orsellinic acid biosynthesis (*pks1*—*Coprinus cinereus*, *pks14*—*Fusarium graminearum*). Likewise, clade β presents multiple examples of biosynthesis of derivatives of orsellinic acid as the first intermediate metabolites (6-MOS, tropolone, violaceol, mitorubrinic acid—clades α and β). A final argument is provided by the unequivocal placement of *Ustilago/Sporisorium* clade within the γ clade of NR-PKSs (by both BI and ML inference), as well as the confirmed orsellinic acid biosynthesis (*Aspergillus nidulans* gene *orsA*, predicted to be of transferred origin) in the early diverging γ.C2–C7a clade. Notably, although grouping of these three clades is resolved differently by original gene trees (β as an outgroup to α/γ—notion supported by divergent signatures of C-terminal thioesterase, as well as presence of the methyltransferase domain throughout β), the support for the groups themselves is strong and unchallenged.

### Ancient Duplications Underlie a Patchwork of Distant Siblings

As remarked above, the predicted events giving rise to the present-day diversity of aromatic compounds are likely ancient (see fig. 2, for graphical summary). The amalgamated likelihood approach predicts them to be mostly duplications followed by extensive losses (849 predicted loss events according to ALE). By this view, extant nonreducing polyketide synthases constitute a patchwork set of distantly related sibling groups.

In particular, repeated emergence of novel cyclization specificities within the γ clade (C6–C11—emodins, asperthecin, monodictyphenone; and C4–C9—sterigmatocystin, aflatoxins, fusarubin) is shown to be monophyletic and ancient. The topological dating of relevant nodes provided by reconciliations places the origin in the common ancestral lineage of four major classes of filamentous fungi (*Leotiomycetes*, *Sordariomycetes*, *Dothideomycetes*, and *Eurotiomycetes*).

Of the total 87 duplication events inferred by ALE reconciliation, 62 are predicted to have occurred in this common ancestral lineage. Furthermore, three ancestral duplication events are predicted to predate the split between *Basidiomycetes* and *Ascomycetes* (the two duplications underlying division into α, β, and γ clades of NR-PKSs and a singular duplication within the α clade itself). Four duplications are tentatively dated at *Dothideomycetes*–*Eurotiomycetes* split. Slightly higher numbers of more recent duplications are inferred in *Dothideomycetes* and *Eurotiomycetes* (mostly in phytopathogenic *Pleosporales*—2 and geni *Aspergillus*—5 and *Talaromyces*—3). This seems related to their rich secondary metabolite repertoires and a relatively dense sampling of the closely related genomes from the respective species. Notably, such patterns cannot be seen for other extensive secondary metabolite producers such as the saprobic/pathogenic *Metarhizium* species. In *Metarhizium*, the high number of NR-PKS genes seems to stem from less extensive selection and not late duplications (only a singular duplication has affected the *Hypocreales* lineage—resulting in the doubling of aurofusarin ancestor gene).

By the same token, the extensive thinning of NR-PKS repertoire (874 losses) started after the divergence of filamentous fungi (estimated losses for *Sordariomycetes*–*Leotiomycetes* common ancestor—23, for *Dothideomycetes*–*Eurotiomycetes* common ancestor—4) and the losses continue following subsequent divergences. As indicated in the previous paragraph, the selective process did not affect all resulting species equally, with some lineages retaining more of the original diversity.

### Transfers Are a Source of Additional Diversity

A ranked list of the top detected transfers with their ALE-based mappings are summarized in Table 1 (alternative scenarios found by DTL-RANGER can be examined on the results website). Briefly, 14 of the 25 transfers (56%) are detected consistently even prior to amalgamation (i.e., the bipartition is present in both amalgamated and original gene trees, the transfer is predicted on both topologies by DTL-RANGER across multiple cost thresholds). The detailed results, obtained from sampling of multiple optimal reconciliations at random, are available through the gene tree visualization (at http://cropnet.pl/metasites/sekmet/nrpks_2014/genetree) by following links to individual node numbers.

**Fig. 5.—:**
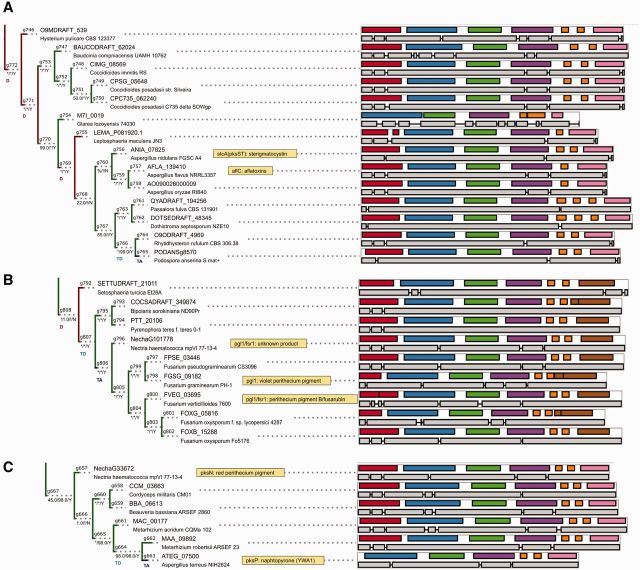
Selected examples of predicted horizontal transfers, acting as additional source of diversity: (*A*) Origin of sterigmatocystin biosynthesis in *P. anserina* (predicted donor in *Rhytidhysteron rufulum* lineage), (*B*) origin of fusarubin biosynthesis in *Fusarium* sp. (HGT from *Pleosporales*), (*C*) origin of putative *pksP* pigment biosynthetic gene into *A. terreus*. Visual conventions (branch support, exon, domains) analogous to figure 4, see also table 1 (summary of predicted HGT events). Trees are not drawn to scale.

Most predicted transfers are fairly recent in comparison to duplications (fig. 2), in keeping with majority of the transfers being lost over time. The key predicted transfer events are predominantly in clade γ (21 transfers) and include several cases of transfers involving well-characterized core biosynthetic genes: HGT of the norsolorinic acid NR-PKS into *Podospora anserina* genome (fig. 5*A*), as well as transfers of pigment-related NR-PKSs (fusarubins—fig. 5*B*, acquisition of a putative *pksP* homolog by *Aspergillus terreus* from *Metarhizium*, fig. 5*C*). Very few transfers are predicted in clade β and no transfers are indicated (postamalgamation) for the early-diverging clade α.

The former example of sterigmatocystin biosynthetic cluster in *P. anserina* constitutes perhaps the best documented individual case of horizontal transfer involving a highly toxic and large secondary metabolite biosynthetic cluster (Slot and Rokas 2011). In our analysis, both the support for the event itself and its mappings are consistent. Interestingly, the prediction indicates transfer donor in *Dothideomycetes*, within *R. rufulum* lineage. Notably, this origin as unfragmented cluster in *Dothideomycetes* has been postulated in a recent paper (Bradshaw et al. 2013).

In some cases, the ALE has resulted in a significantly different scenario from simply sampling the optimal reconciliations based on the original tree topology. For a singular *Tuber melanosporum* NR-PKS (*GSTUM_00003432001*), its placement in the original BI tree (at 74% BPP, Bayesian Posterior Probability) has introduced an apparent HGT from early *Pezizomycotina* to *Basidiomycetes*. This scenario is rejected by ALE where an alternative grouping is incorporated in the final tree instead (bipartition *g31* supported at 26% BPP; see fig. 4). This rearrangement shows that consideration of alternative rather than majority bipartitions is required for validating transfer events, even at most restrictive cost settings.

As the sampling of taxa is not even across the species tree, one can expect that increasing coverage of undersampled species will reveal more lineage-specific duplications and transfer events. This is well demonstrated by *Eurotiales* clade. For this reason, we refrain from making quantitative calls about the singular numbers of events. Still, some observations stand out. In particular, the branch leading to *Glarea lozoyensis* (*Helotiales*) appears to be a rather frequent donor (four donated transfers). This is partially explained by both the length of the corresponding branch of the species tree (by the dated chronogram the split between *Glarea* and *B. graminis* lines occurred ∼250 Ma) as well as the other represented species being plant pathogenic rather than saprobic throughout their lifecycle. Also, a high number of transfers (8) are predicted within the γ.C2–C7a subclade (represented by *orsA* and *zea1*) which presents a sparse distribution of homologs from distantly related species. Although artifactual origins are possible (genes strongly differ in structure), HGT was previously (Xu et al. 2014) invoked as an explanation of the observed diversity of macrocyclic lactones. Additionally, the highly reducing polyketide synthases (indicative of the biosynthesis of lactone part not derived from orsellinic acid molecule) are strongly enriched for the entire γ.C2–C7a clade (as well as part of the β clade containing the asperfuranone biosynthetic NR-PKS).

### Speciation, Duplication, and Transfer Differentially Affected the Genomic Context

We investigated the overall differences in conservation of genomic context (+20/−20 genes) at each bipartition (in the amalgamated tree) by looking at the average number of conserved homologs shared between neighborhoods of any two members of the respective descendant clades (left and right subtrees of inner nodes in the tree). This estimate was then contrasted with event labels ascribed to bipartition by ALE. Duplications were found to disrupt the synteny most (11% of shared homolog groups on the average are retained), with significantly (Wilcoxon ranked sum test) higher retention for speciation (40%; *P* < 10^−^^5^) and slightly higher for transfers (21%, not significant). The number of the putative counterparts decays with the depth of the node (Pearson correlation between the ratio of shared homologs vs. depth of the node in the tree ρ = −0.22, *P* = 5 × 10^−^^6^), confirming that context is also lost over multiple events (in part due to most of the deeper nodes being duplications, while many recent ones are speciations). Notably, we think that these estimates err on the strong side as counterparts were identified on basis of membership in the same CHG (gene family or subfamily) rather than precise orthology/paralogy relationships.

Although for some of the predicted transfer cases the genomic context is partially preserved (e.g., sterigmatocystin in *Podospora—*17% genes have a counterpart in *Rhytidhysteron*), there are five cases (*g361*, *g511*, *g664*, *g731*, *g791*), including the strongly supported transfer of the *pksP* into *A. terreus* (*g664*), where genomic neighborhood is not shared between predicted donor and acceptor branches at all.

### Conserved Splice Junctions and Overrepresented Accessory Enzymes Are Associated with Emergence of Different Cyclizations

The gene groups significantly overrepresented in association with particular monophyletic clades are summarized in table 2 (graphic summary of selected associations is shown on fig. 3). Out of 132 homolog groups, 40 display significant (*P* < 0.0001) enrichment for at least one monophyletic clade in the tree (possibly indicating the point of origin for acquiring the accessory enzyme). Strikingly, no enrichment of any particular gene family was noted in association with the early diverging clade α (which contains sequences from both *Basidiomycetes* and *Ascomycetes*). This trend was not mirrored in exon junction positions which are conserved relative to consensus sequences of KS and AT domains encoded by α clade members.

In terms of splicing site conservation, all three clades (α/β/γ) demonstrate specific patterns (see supplementary table S8, Supplementary Material online). In group α, the member genes typically present higher overall number of exons due to multiple splice junctions embedded within the above-mentioned KS and AT domains. In group β, the core NR-PKS domains are typically encoded on a single large exon, with a clear exception of the clade (*g182*) associated with biosynthesis of meroterpenoid products (terretonin, austinol). The meroterpenoid NR-PKSs show their own characteristic pattern of splice junctions within SAT, KS, and AT domains.

**Fig. 6.—:**
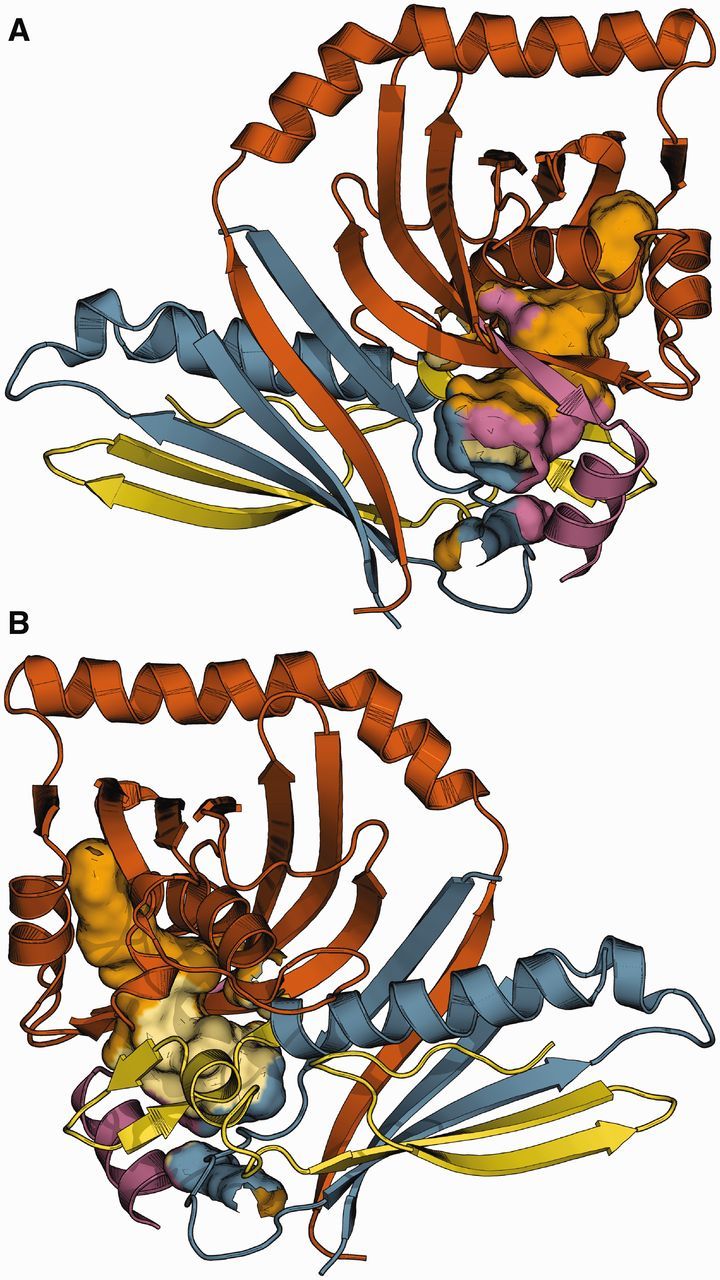
The fragmentation of PT domain into pieces encoded on different exons facilitates gated access to cyclization chamber (clade γ). The structural relationships are visualized on *A. parasiticus* PksA model (PDB: 3HRQ, chain A). Different colors correspond to fragments encoded on separate exons. The large, filled shape corresponds to the inner surface of cyclization chamber, where all exons contribute to the chamber entrance area. Views from the front (*A*) and back (*B*) are both shown.

The γ clade of NR-PKSs encompasses synthases capable of a number of divergent modes of cyclization (C2–C7, C6–C11, C4–C9; Li et al. 2010). This plasticity of function is perhaps facilitated by the observed tendency toward exon gain within both the SAT and the PT domains. Notably, by relating to available protein structure of the PT domain (norsolorinic acid synthase from *Aspergillus parasiticus*, C4–C9 cyclization type) one can see that the highest degree of fragmentation results in four individually encoded pieces, each of which contributes to the gating of the cyclization chamber (see fig. 6—the model of PksA aflatoxin NR-PKS with delineated structural elements corresponding to coding sequence parts separated by splice junctions).

The subfunctionalization toward different cyclization schemes and/or end products is also shown to be related to recruitment of specific accessory enzymes. The manganese-dependent β-lactamases are unambiguously associated with γ.C6–C11 subclade (*g552*, corrected *P* = 8.6e-43), with the notable exception of endocrocin sister clade (*g439*; where β-lactamase is not always present in the vicinity of NR-PKS). Taken as an exemplary association, the endocrocin example demonstrates that strong linkage follows recruitment of the accessory enzyme and thus provides an upper constraint on the timing of the original involvement in the biosynthetic pathway.

As with most of the predicted duplications, the topological dating places this tight linkage with β-lactamases (Li et al. 2011), prior to the divergence of major classes of filamentous fungi. As in other clades, the syntenic relationships persist through multiple predicted horizontal transfer events, involving among others: The acquisition of capacity to synthesize alternariol/isocoumarins by *A. nidulans*/*oryzae*/*flavus* (*pkgA* homologs, predicted to originate from *Sordariomycetes*, HGT supported solely by ALE) and spread of asperthecin biosynthetic PKS *aptA* homologs to *Arthroderma* sp. as well as *Metarhizium* sp. (with the original donor in *Aspergillus fischerii*/*fumigatus* clade—a likely ortholog of the extant neosartoricin NR-PKS *nscA* involved in prenylated xanthone biosynthesis).

The enrichment analysis suggests that diversification of activity was also associated with recruitment of conserved but divergent groups of transcription factors (of the zinc-binding finger variety). Of the five transcription factor groups, only one (*ch43*) is enriched for two separate subclades of γ. The only other case, where a homolog group is strongly enriched in more than one part of the tree, concerns the highly reducing polyketide synthases mentioned in the previous subsection.

As a sidenote, there are multiple cases where accessory enzymes strongly associated with a monophyletic clade are nevertheless present (in singular numbers) around NR-PKS genes from different clades (see table 2, e.g.: Several aflatoxin biosynthetic cluster genes, aurofusarin biosynthesis dehydratase *aurZ*). Depending on the respective gene family histories, the presence of such “outliers” raises the salient questions of frequency and modes of exchange of accessory enzymes between different clusters, particularly if biosynthetic activities of individual clusters are linked by common end product further down the line (e.g., Andersen et al. 2013).

### The Evolution of Pigment Biosynthesis Presents Both Conservation and Diversification

**Fig. 7.—:**
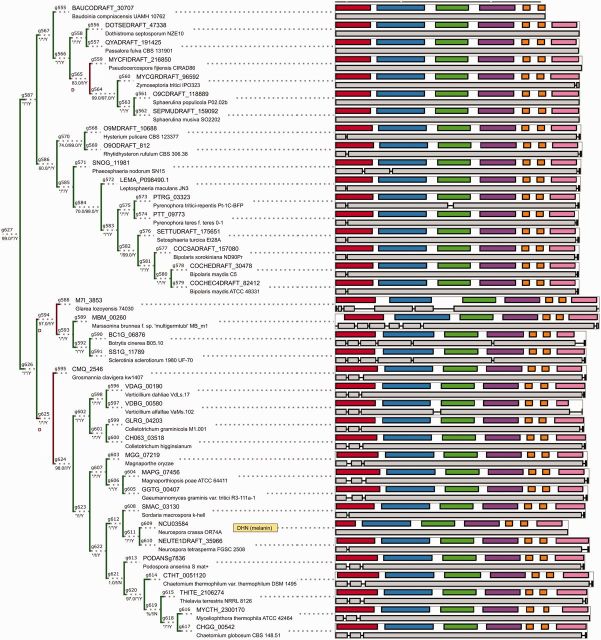
The phylogeny of tetrahydroxynaphthalene synthases (the core enzyme of melanin biosynthesis) mirrors speciation explicitly in the majority of *Dothideomycetes*, *Leotiomycetes,* and *Sordariomycetes.* Visual conventions (branch support, exon, domains) analogous to figure 4. Gene models for MBM_00260 and M7I_3853 were truncated from 5′ and 3′ sides, respectively.

Perhaps, the most striking examples of both conservation and variation concern the evolution of melanin biosynthesis (see fig. 7) and splitting of the biosynthesis of alternative pigments and toxins from this conserved branch. First, the common ancestor of γ.C6–C11, γ.C2–C7b/c, and γ.C4–C9 (fig. 3) acquired an accessory enoyl-CoA reductase (of the short chain dehydrogenase superfamily). This likely enabled biosynthesis of reduced intermediate compounds (such as versicolorin during the biosynthesis of aflatoxins, tetra/trihydrohydroxynaphthalene during biosynthesis of melanins, monodictyphenone during the biosynthesis of xanthones). Next, the linkage with copper-dependent laccase enzymes enabled oxidative polymerization (e.g., in biosynthesis of melanins, aurofusarin) in the common ancestor γ.C2–C7b/c and γ.C4–C9.

Movement between different components of the (two-speed) fungal genome is evidenced in the placement of melanin biosynthetic NR-PKSs (including the genes involved in biosynthesis of melanin through naphtopyrone intermediate) in the vicinity of confirmed housekeeping genes (see table 2). Conservation is further underscored by inheritance of the tetrahydroxynaphtalene synthase (core NR-PKS) as an ortholog in the majority of filamentous fungi (fig. 7—most of *Leotiomycetes*, *Sordariomycetes*, and *Dothideomycetes*). In select *Sordariomycetes* (*Hypocreales*) as well as most *Eurotiomycetes*, pigment biosynthesis is instead carried out through modified naphtopyrone routes (through YWA1 heptaketide intermediate product—e.g., Chiang et al. 2011). Notably both the speciation patterns and the conserved gene structure of the core melanin biosynthetic NR-PKS (the tetrahydroxynaphthalene synthase) suggest possible application as a source of additional barcode markers across the relevant taxa.

The basic building blocks (accessory enzymes) partaking in biosynthesis of melanins have been further retained for biosynthesis of other compounds (and are present in the extant clusters). Furthermore, possibly due to the increased toxicity of C4–C9 cyclization products (such as sterigmatocystin and aflatoxins, javanicin, and fusarubins) that subclade is associated with significantly overrepresented retention of accessory methyltransferases in the close neighborhood of the core megasynthase.

**Fig. 8.—:**
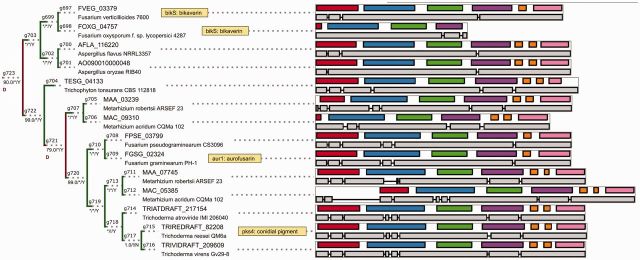
The parology/orthology relationships of bikaverin, aurofusarin, and *Trichoderma* conidial pigment core polyketide synthases are due to an ancestral duplication in filamentous fungi (*g723*), followed by an additional duplication in *Hypocreales* (*g721*). Visual conventions (branch support, exon, domains) are analogous to figure 4. Tree fragment is not drawn to scale.

The analysis of top-scoring, consistently predicted transfer events suggests that biosynthesis of aromatic pigments, following the divergence of genes involved in melanin biosynthesis through the DHN route, could have been influenced by key transfer events (e.g., origin of *Pgl1*/*Fsr1* orthologs in *Fusarium* as a horizontal transfer from early *Pleosporales*, reintroduction of a putative pigment biosynthesis gene into *A. terreus* as a transfer from *Metarhizium*). As an example of finer resolution, due to the increased taxon sampling of our analysis (relative to earlier phylogenomic analysis by Brown et al. [2012]), the core genes of bikaverin and aurofusarin biosynthesis are now shown to be nonorthologous in origin. These NR-PKSs are now revealed to belong to sister clades which diverged early during the evolution of filamentous *Ascomycota* and (in case of aurofusarin NR-PKS) underwent an additional *Hypocreales*’ specific duplication. The aurofusarin biosynthetic gene is however shown to be orthologous to experimentally characterized *Trichoderma* PKS4 (pigment synthesis PKS; Atanasova et al. 2013), following the said duplications (fig. 8).

Last point of note concerns the replacement of thioesterase domain with NAD-binding reductive domain leading to aldehyde end products of cyclization. This event has likely occurred twice during the evolution of NR-PKSs (respectively, in γ.C4–C9 clade leading to fusarubin subclade—fig. 5*B*—and in the part of the β clade associated with biosynthesis of azaphilones and tropolone-derived compounds). Based on the gene structure and similarity of protein sequences of affected domains, a likely possibility is the recombination (or fusion) between a donor gene from β clade and an acceptor gene from γ clade (or vice versa). Regardless of whether the domain has been acquired from an outside or inside source, the implied scenarios show the need for considering mosaic ancestry of multidomain proteins, where different regions of the protein/gene could have conflicting origins.

### Toward Phylogenomic Roadmaps of Secondary Metabolite Biosynthesis in Fungi

The reconstruction of evolutionary history of fungal NR-PKSs faced a number of obstacles. Foremost among these was the quality of gene tree inference for a large and diversified gene (sub)family across multiple species. First, the data are strongly saturated worsening the risks associated with long branch attraction and incorrect resolution of deeper nodes in the tree. Second, due to being focused on a conserved fragment of limited length (the KS–AT core modules and intervening region) attempts to put conservative filters on data, commonly accepted in species-focused phylogenomics, would likely introduce stochastic bias (particularly) in the resolution of rapid successions of events. Nevertheless, we have taken several precautions against the common artifacts of phylogeny reconstruction (use of protein sequences, filtering protein alignments based on transitive consistency score, use of different methods for phylogeny reconstruction including models able to take into account the heterogeneity of rates across informative sites).

In its present iteration, the reconstruction uncertainty is also alleviated by conscious inclusion of species trees in phylogeny reconstruction process (so in the absence of prevailing evidence, neutral scenario implied by species tree topology is considered by ALE). The contemporary developments in probabilistic models for inferring both species and gene phylogeny simultaneously (Boussau et al. 2013) and for inferring the gene phylogeny based on simultaneous reconciliation with a putative species tree (Szollosi, Rosikiewicz, et al. 2013) formalize this approach in a probabilistic framework. In our example, the application of ALE has also given an added benefit of explicitly modelling the two-step transfers through intermediary donors from extinct lineages (Szollosi, Tannier, et al. 2013).

The results demonstrate the utility of maximally parsimonious reconciliations and phylogenetic inference in uncovering the sources of extant diversity in a highly diverged gene family (duplication, transfer, and selective loss). As a proof-of-concept our analysis demonstrates that in considering the evolution of secondary metabolism in microbial *Eukaryotes*, the scientists have to take into account transfers (at varying degrees) as a viable origin hypothesis for present metabolite diversity. This is even though, in this case, majority of molecular innovation seems ancient in origin and (throughout the history of multiple extant fungal lineages) has been subjected mostly to selective losses. The use of probabilistic methods (ALE) allowed us to address the parameter choice (costs of different events) in an unsupervised way—with additional support lent to early diversification of biosynthetic mechanisms and a limited, but not negligible, number of predicted transfer events.

In the future, the increased sampling of previously uncharacterized taxa of higher fungi will likely increase the number and improve the resolution of individual events. The biases in taxonomic coverage resulting from uneven sampling of divergent taxa are liable to influence what is perceived as an incorrect outlier (errant bipartition due to inclusion of a gene from a rogue taxa) rather than accumulated evidence from multiple, more distantly related species (Szollosi et al. 2015). The information about spread and retention of biosynthetic potentials in different lineages may thus reveal stronger and/or different trends among donor and receiver groups, as well as contrast these across ecological niches occupied by related organisms. The resolution of transfers, in particular can be challenged, should novel organisms from related clades (the increasing public coverage of previously unsampled lineages, such as *Chaetothyriales*, *Lecanoromycetes* or *Xylonomycetes*) imply different distributions of genes. This is one of the reasons why roadmap nature of the resource should be emphasized—one taking into account the “landmarks” (species) available at the time of its creation.

Likewise, the continuous development of novel computational approaches taking advantage of high-performance computing infrastructure results in more accessible methods increasingly able to account for some of the unavoidable biases in the data. For example, both rate heterogeneity and misleading signals, arising due to saturation, are better handled by improved Bayesian and ML methods, as evidenced by a number of studies (e.g., Boussau and Gouy 2006; Lartillot et al. 2009; Husník et al. 2011).

Last but not least aspect of the presented analysis lies in providing, for the first time, a phylogeny-based annotation of NR-PKS core genes involved in fungal secondary metabolism. With increasing number of inquiries into the phylogenetic basis of eukaryotic secondary metabolism based on functional experiments (knockout, deletion mutant libraries) and growing genomic coverage, similar initiatives should complement large tools and databases (Khaldi et al. 2010; Blin et al. 2013; Wang et al. 2014). In particular, a phylogeny-centric resource should facilitate research focused on chemotaxonomy, as well as further dissection of the evolutionary fates of specific compounds and clusters (e.g., adaptation of pigment molecules for protection from both abiotic and biotic stresses, emergence and spread of high toxigenic potential).

## Conclusions

We present, for the first time, a phylogeny-based analysis elucidating the origins of diversity in one of the largest and most important groups of core genes involved in fungal secondary metabolism—the nonreducing polyketide synthases. The results support ancient duplications of a limited number of NR-PKSs into subsets catalyzing divergent chemical reactions. The highly diverse representation of these genes in extant fungal genomes is revealed to be a result of subsequent, large-scale selective losses, moderated by a number of more recent duplications and horizontal transfers (associated with, e.g., emergence of fusarubin biosynthesis in fusaria). The complementary analysis of phylogeny-associated traits (genomic neighborhood, splice junctions) shows gain of tightly linked accessory genes and modularization of key PKS domains (PT domain), as crucial traits accompanying the functional diversification.

## Supplementary Material

Supplementary Data

## References

[evv204-B1] Aguileta G 2008 Assessing the performance of single-copy genes for recovering robust phylogenies. Syst Biol. 57:613–627. 18709599 10.1080/10635150802306527

[evv204-B2] Ahuja M 2012 Illuminating the Diversity of Aromatic Polyketide Synthases in *Aspergillus nidulans*. J Am Chem Soc. 134:8212–8221. 22510154 10.1021/ja3016395 PMC3357392

[evv204-B3] Andersen MR 2013 Accurate prediction of secondary metabolite gene clusters in filamentous fungi. Proc Natl Acad Sci U S A. 110:E99–E107. 23248299 10.1073/pnas.1205532110 PMC3538241

[evv204-B4] Anisimova M 2013 State-of the art methodologies dictate new standards for phylogenetic analysis. BMC Evol Biol. 13:161. 23914788 10.1186/1471-2148-13-161 PMC3751533

[evv204-B5] Atanasova L Knox BP Kubicek CP Druzhinina IS Baker SE 2013 The polyketide synthase gene pks4 of *Trichoderma reesei* provides pigmentation and stress resistance. Eukaryot Cell. 12:1499–1508. 24036343 10.1128/EC.00103-13 PMC3837940

[evv204-B6] Bansal MS Alm EJ Kellis M 2012 Efficient algorithms for the reconciliation problem with gene duplication, horizontal transfer and loss. Bioinformatics 28:i283–i291. 22689773 10.1093/bioinformatics/bts225 PMC3371857

[evv204-B7] Bansal MS Alm EJ Kellis M 2013 Reconciliation revisited: handling multiple optima when reconciling with duplication, transfer, and loss. J Comp Biol. 20:738–754. 10.1089/cmb.2013.0073 PMC3791060 24033262

[evv204-B8] Benson DA 2015 GenBank. Nucleic Acids Res. 43:D30–D35. 25414350 10.1093/nar/gku1216 PMC4383990

[evv204-B9] Bergmann S 2007 Genomics-driven discovery of PKS-NRPS hybrid metabolites from *Aspergillus nidulans*. Nat Chem Biol. 3:213–217. 17369821 10.1038/nchembio869

[evv204-B10] Bie TD Cristianini N Demuth JP Hahn MW 2006 CAFE: a computational tool for the study of gene family evolution. Bioinformatics 22:1269–1271. 16543274 10.1093/bioinformatics/btl097

[evv204-B11] Blin K 2013 antiSMASH 2.0—a versatile platform for genome mining of secondary metabolite producers. Nucleic Acids Res. 41:W204–W212. 23737449 10.1093/nar/gkt449 PMC3692088

[evv204-B12] Boussau B 2013 Genome-scale coestimation of species and gene trees. Genome Res. 23:323–330. 23132911 10.1101/gr.141978.112 PMC3561873

[evv204-B13] Boussau B Gouy M 2006 Efficient likelihood computations with nonreversible models of evolution. Syst Biol. 55:756–768. 17060197 10.1080/10635150600975218

[evv204-B14] Bradshaw RE 2013 Fragmentation of an aflatoxin-like gene cluster in a forest pathogen. New Phytol. 198:525–535. 23448391 10.1111/nph.12161

[evv204-B15] Brown DW Butchko RAE Baker SE Proctor RH 2012 Phylogenomic and functional domain analysis of polyketide synthases in *Fusarium*. Fungal Biol. 116:318–331. 22289777 10.1016/j.funbio.2011.12.005

[evv204-B16] Bushley KE Turgeon BG 2010 Phylogenomics reveals subfamilies of fungal nonribosomal peptide synthetases and their evolutionary relationships. BMC Evol Biol. 10:26. 20100353 10.1186/1471-2148-10-26 PMC2823734

[evv204-B17] Capella-Gutiérrez S Silla-Martínez JM Gabaldón T 2009 trimAl: a tool for automated alignment trimming in large-scale phylogenetic analyses. Bioinformatics 25:1972–1973. 19505945 10.1093/bioinformatics/btp348 PMC2712344

[evv204-B18] Chan CX Mahbob M Ragan MA 2013 Clustering evolving proteins into homologous families. BMC Bioinformatics 14:120. 23566217 10.1186/1471-2105-14-120 PMC3637521

[evv204-B19] Chang J-M Di Tommaso P Notredame C 2014 TCS: a new multiple sequence alignment reliability measure to estimate alignment accuracy and improve phylogenetic tree reconstruction. Mol Biol Evol. 31:1625–1637. 24694831 10.1093/molbev/msu117

[evv204-B20] Chiang Y-M 2011 Characterization of a polyketide synthase in *Aspergillus niger* whose product is a precursor for both dihydroxynaphthalene (DHN) melanin and naphtho-γ-pyrone. Fungal Genet Biol. 48:430–437. 21176790 10.1016/j.fgb.2010.12.001 PMC3118676

[evv204-B21] Cock PJA 2009 Biopython: freely available Python tools for computational molecular biology and bioinformatics. Bioinformatics 25:1422–1423. 19304878 10.1093/bioinformatics/btp163 PMC2682512

[evv204-B22] Cox RJ 2007 Polyketides, proteins and genes in fungi: programmed nano-machines begin to reveal their secrets. Org Biomol Chem. 5:2010–2026. 17581644 10.1039/b704420h

[evv204-B23] Darriba D Taboada GL Doallo R Posada D 2011 ProtTest 3: fast selection of best-fit models of protein evolution. Bioinformatics 27:1164–1165. 21335321 10.1093/bioinformatics/btr088 PMC5215816

[evv204-B24] Delgado JA Al-Azzam O Denton AM Markell SG Goswami RS 2012 A resource for the in silico identification of fungal polyketide synthases from predicted fungal proteomes. Mol Plant Pathol. 13:494–507. 22112245 10.1111/j.1364-3703.2011.00760.x PMC6638892

[evv204-B25] Douzery EJP Snell EA Bapteste E Delsuc F Philippe H 2004 The timing of eukaryotic evolution: does a relaxed molecular clock reconcile proteins and fossils? Proc Natl Acad Sci U S A. 101:15386–15391. 10.1073/pnas.0403984101 PMC524432 15494441

[evv204-B26] Doyon J-P Ranwez V Daubin V Berry V 2011 Models, algorithms and programs for phylogeny reconciliation. Brief Bioinform. 12:392–400. 21949266 10.1093/bib/bbr045

[evv204-B27] Ebersberger I 2012 A consistent phylogenetic backbone for the fungi. Mol Biol Evol. 29:1319–1334. 22114356 10.1093/molbev/msr285 PMC3339314

[evv204-B28] Eddy SR 2009 A new generation of homology search tools based on probabilistic inference. Genome Inform. 23:205–211. 20180275

[evv204-B29] Enright AJ Van Dongen S Ouzounis CA 2002 An efficient algorithm for large-scale detection of protein families. Nucleic Acids Res. 30:1575–1584. 11917018 10.1093/nar/30.7.1575 PMC101833

[evv204-B30] Felsenstein J 1989 PHYLIP—Phylogeny Inference Package (Version 3.2). Cladistics 5:164–166.

[evv204-B31] Finn RD 2014 Pfam: the protein families database. Nucleic Acids Res. 42:D222–D230. 24288371 10.1093/nar/gkt1223 PMC3965110

[evv204-B32] Flicek P 2014 Ensembl 2014. Nucleic Acids Res. 42:D749–D755. 24316576 10.1093/nar/gkt1196 PMC3964975

[evv204-B33] Foerstner KU Doerks T Creevey CJ Doerks A Bork P 2008 A computational screen for type I polyketide synthases in metagenomics shotgun data. PLoS Onee3515. 18953415 10.1371/journal.pone.0003515 PMC2568958

[evv204-B34] Frickey T Lupas A 2004 CLANS: a Java application for visualizing protein families based on pairwise similarity. Bioinformatics 20:3702–3704. 15284097 10.1093/bioinformatics/bth444

[evv204-B35] Gerke J 2012 Breaking the silence: protein stabilization uncovers silenced biosynthetic gene clusters in the fungus *Aspergillus nidulans*. Appl Environ Microbiol. 78:8234–8244. 23001671 10.1128/AEM.01808-12 PMC3497355

[evv204-B36] Grigoriev IV 2012 The genome portal of the Department of Energy Joint Genome Institute. Nucleic Acids Res. 40:D26–D32. 22110030 10.1093/nar/gkr947 PMC3245080

[evv204-B37] Gueidan C Ruibal C de Hoog GS Schneider H 2011 Rock-inhabiting fungi originated during periods of dry climate in the late Devonian and middle Triassic. Fungal Biol. 115:987–996. 21944211 10.1016/j.funbio.2011.04.002

[evv204-B38] Huerta-Cepas J Dopazo J Gabaldón T 2010 ETE: a python Environment for Tree Exploration. BMC Bioinformatics 11:24. 20070885 10.1186/1471-2105-11-24 PMC2820433

[evv204-B39] Husník F Chrudimský T Hypša V 2011 Multiple origins of endosymbiosis within the *Enterobacteriaceae* (*γ-Proteobacteria*): convergence of complex phylogenetic approaches. BMC Biol. 9:87. 22201529 10.1186/1741-7007-9-87 PMC3271043

[evv204-B40] Katoh K Standley DM 2013 MAFFT multiple sequence alignment software version 7: improvements in performance and usability. Mol Biol Evol. 30:772–780. 23329690 10.1093/molbev/mst010 PMC3603318

[evv204-B41] Khaldi N 2010 SMURF: genomic mapping of fungal secondary metabolite clusters. Fungal Genet Biol. 47:736–741. 20554054 10.1016/j.fgb.2010.06.003 PMC2916752

[evv204-B42] Khaldi N Wolfe KH 2011 Evolutionary origins of the fumonisin secondary metabolite gene cluster in *Fusarium verticillioides* and *Aspergillus niger*. Int J Evol Biol. 2011:423821. 21716743 10.4061/2011/423821 PMC3119522

[evv204-B43] Kroken S Glass NL Taylor JW Yoder OC Turgeon BG 2003 Phylogenomic analysis of type I polyketide synthase genes in pathogenic and saprobic ascomycetes. Proc Natl Acad Sci U S A. 100:15670–15675. 14676319 10.1073/pnas.2532165100 PMC307626

[evv204-B44] Lartillot N Lepage T Blanquart S 2009 PhyloBayes 3: a Bayesian software package for phylogenetic reconstruction and molecular dating. Bioinformatics 25:2286–2288. 19535536 10.1093/bioinformatics/btp368

[evv204-B45] Lartillot N Rodrigue N Stubbs D Richer J 2013 PhyloBayes MPI: phylogenetic reconstruction with infinite mixtures of profiles in a parallel environment. Syst Biol. 62:611–615. 23564032 10.1093/sysbio/syt022

[evv204-B46] Li Y Chooi Y-H Sheng Y Valentine JS Tang Y 2011 Comparative characterization of fungal anthracenone and naphthacenedione biosynthetic pathways reveals an α-hydroxylation-dependent Claisen-like cyclization catalyzed by a dimanganese thioesterase. J Am Chem Soc. 133:15773–15785. 21866960 10.1021/ja206906d PMC3183131

[evv204-B47] Li Y 2010 Classification, prediction, and verification of the regioselectivity of fungal polyketide synthase product template domains. J Biol Chem. 285:22764–22773. 20479000 10.1074/jbc.M110.128504 PMC2906267

[evv204-B48] Marchler-Bauer A 2015 CDD: NCBI’s conserved domain database. Nucleic Acids Res. 43:D222–D226. 25414356 10.1093/nar/gku1221 PMC4383992

[evv204-B49] Marthey S 2008 FUNYBASE: a FUNgal phYlogenomic dataBASE. BMC Bioinformatics 9:456. 18954438 10.1186/1471-2105-9-456 PMC2600828

[evv204-B50] Minh BQ Nguyen MAT von Haeseler A 2013 Ultrafast approximation for phylogenetic bootstrap. Mol Biol Evol. 30:1188–1195. 23418397 10.1093/molbev/mst024 PMC3670741

[evv204-B51] Nielsen ML 2011 A genome-wide polyketide synthase deletion library uncovers novel genetic links to polyketides and meroterpenoids in *Aspergillus nidulans*. FEMS Microbiol Lett. 321:157–166. 21658102 10.1111/j.1574-6968.2011.02327.x

[evv204-B52] O’Donnell K 2013 Phylogenetic analyses of *RPB1* and *RPB2* support a middle Cretaceous origin for a clade comprising all agriculturally and medically important fusaria. Fungal Genet Biol. 52:20–31. 23357352 10.1016/j.fgb.2012.12.004

[evv204-B53] Ohm RA 2012 Diverse lifestyles and strategies of plant pathogenesis encoded in the genomes of eighteen *Dothideomycetes* fungi. PLoS Pathog. 8:e1003037. 23236275 10.1371/journal.ppat.1003037 PMC3516569

[evv204-B54] Price MN Dehal PS Arkin AP 2010 FastTree 2—approximately maximum-likelihood trees for large alignments. PLoS One 5:e9490. 20224823 10.1371/journal.pone.0009490 PMC2835736

[evv204-B55] Proctor RH 2013 Birth, death and horizontal transfer of the fumonisin biosynthetic gene cluster during the evolutionary diversification of *Fusarium*. Mol Microbiol. 90:290–306. 23937442 10.1111/mmi.12362

[evv204-B56] Rose PW 2013 The RCSB Protein Data Bank: new resources for research and education. Nucleic Acids Res. 41:D475–D482. 23193259 10.1093/nar/gks1200 PMC3531086

[evv204-B57] Rousseeuw PJ 1987 Silhouettes: a graphical aid to the interpretation and validation of cluster analysis. J Comput Appl Math. 20:53–65.

[evv204-B58] Sanchez JF Somoza AD Keller NP Wang CCC 2012 Advances in *Aspergillus* secondary metabolite research in the post-genomic era. Nat Prod Rep. 29:351–371. 22228366 10.1039/c2np00084a PMC4568942

[evv204-B59] Schmitt I Lumbsch HT 2009 Ancient horizontal gene transfer from bacteria enhances biosynthetic capabilities of fungi. PLoS One 4:e4437. 19212443 10.1371/journal.pone.0004437 PMC2636887

[evv204-B60] Slot JC Rokas A 2011 Horizontal transfer of a large and highly toxic secondary metabolic gene cluster between fungi. Curr Biol. 21:134–139. 21194949 10.1016/j.cub.2010.12.020

[evv204-B61] Spanu PD 2010 Genome expansion and gene loss in powdery mildew fungi reveal tradeoffs in extreme parasitism. Science 330:1543–1546. 21148392 10.1126/science.1194573

[evv204-B62] Sung G-H Poinar GO Jr Spatafora JW 2008 The oldest fossil evidence of animal parasitism by fungi supports a Cretaceous diversification of fungal–arthropod symbioses. Mol Phylogenet Evol. 49:495–502. 18817884 10.1016/j.ympev.2008.08.028

[evv204-B63] Szollosi GJ Davín AA Tannier E Daubin V Boussau B 2015 Genome-scale phylogenetic analysis finds extensive gene transfer among fungi. Philos Trans R Soc Lond B Biol Sci. 370(1678). pii:20140335. 10.1098/rstb.2014.0335 PMC4571573 26323765

[evv204-B64] Szollosi GJ Rosikiewicz W Boussau B Tannier E Daubin V 2013 Efficient exploration of the space of reconciled gene trees. Syst Biol. 62:901–912. 23925510 10.1093/sysbio/syt054 PMC3797637

[evv204-B65] Szollosi GJ Tannier E Lartillot N Daubin V 2013 Lateral gene transfer from the dead. Syst Biol. 62:386–397. 23355531 10.1093/sysbio/syt003 PMC3622898

[evv204-B66] Talevich E Invergo BM Cock PJ Chapman BA 2012 Bio.Phylo: a unified toolkit for processing, analyzing and visualizing phylogenetic trees in Biopython. BMC Bioinformatics 13:209. 22909249 10.1186/1471-2105-13-209 PMC3468381

[evv204-B67] UniProt Consortium. 2014 Activities at the Universal Protein Resource (UniProt). Nucleic Acids Res. 42:D191–D198. 24253303 10.1093/nar/gkt1140 PMC3965022

[evv204-B68] Wang H Fewer DP Holm L Rouhiainen L Sivonen K 2014 Atlas of nonribosomal peptide and polyketide biosynthetic pathways reveals common occurrence of nonmodular enzymes. Proc Natl Acad Sci U S A. 111:9259–9264. 24927540 10.1073/pnas.1401734111 PMC4078802

[evv204-B69] Wang H Xu Z Gao L Hao B 2009 A fungal phylogeny based on 82 complete genomes using the composition vector method. BMC Evol Biol. 9:195. 19664262 10.1186/1471-2148-9-195 PMC3087519

[evv204-B70] Xu Y 2014 Insights into the biosynthesis of 12-membered resorcylic acid lactones from heterologous production in *Saccharomyces cerevisiae*. ACS Chem Biol. 9:1119–1127 24597618 10.1021/cb500043g PMC4033647

