# Supplementary material for: The Distant Siblings—A Phylogenomic Roadmap Illuminates the Origins of Extant Diversity in Fungal Aromatic Polyketide Biosynthesis
Source: Genome Biol Evol. 2015 Nov 3;7(11):3132–54. doi: 10.1093/gbe/evv204 (PMC5635595; doi:10.1093/gbe/evv204)
Supplement: Supplementary Data [file SM1_tables_figures.pdf › SM1_tables_figures.pdf]

List of contents:

Tables:

Supplementary Table ST1. Model genomes used in the phylogenomic analysis. Asterisk denotes genome assemblies which have been available via GenBank for at least 1 year without formal publication.

Supplementary Table ST2. Single-copy, orthologous, housekeeping genes chosen for species tree reconstruction. Choice was made on basis of topological score over 90% (as reported in FUNYBASE (Aguileta et al. 2008; Marthey et al. 2008)).

Supplementary Table ST3. Dating constraints introduced for calibration. All ages are denoted in million years. Delinating taxa are given for most recent common ancestor (MRCA) identification of the dated node.

Supplementary Table ST4. Reference non-reducing PKSs with known activity and/or (end) products.

Supplementary Table ST5. Event counts and optimal solution costs across different parameter values for DTL-RANGER analysis of the original (Bayesian Inference consensus) and amalgamated (ALE) tree topologies. The shown parameter combinations are limited to transfer costs between 5 and 20. DTL-RANGER solutions could not be obtained for the cost combination of <D=4, T=5, L=3> (indicated by N/A values).

Supplementary Table ST6. Fraction of event labels (speciation, transfer donor, duplication) shared between DTL-RANGER and ALE for both original and amalgamated (ALE) tree topology. For DTL-RANGER type of event was determined by frequency within sampled reconciliations. Solutions could not be obtained for the cost combination of <D=4, T=5, L=3> (indicated by N/A values). The shown parameter combinations are limited to transfer costs between 5 and 20. For the original tree (Bayesian consensus) only the 352 non-rearranged bipartitions (shared between amalgamated and original trees) were taken into account.

Supplementary Table ST7. Fraction of event mappings (species tree nodes) shared between DTL-RANGER and ALE for both original and amalgamated (ALE) trees. Measure is tentative as ALE and DTL-RANGER model transfer process differently (with ALE explicitly modelling two-step transfer through extinct intermediate lineages). Mapping was taken to be in accord, if (all of) the most frequent predictions by DTL-RANGER were indicated in the ALE output for given node. Solutions could not be obtained for the cost combination of <D=4, T=5, L=3> (indicated by N/A values). The shown parameter combinations are limited to transfer costs between 5 and 20. For the original tree (Bayesian consensus) only the 352 non-rearranged bipartitions (shared between amalgamated and original trees) were taken into account.

Supplementary Table ST8. Splice site locations significantly associated with monophyletic clades (P<0.0001). Locations mapped to consensus sequences of core NR-PKS domains (SAT – starter acyl transferase, KS – ketoacyl synthase, AT – acyl transferase, PT – product template, TE – thioesterase). Inherited splice junctions (i.e. overrepresented at an ancestor node) are given in parentheses.

Figures:

Supplementary Figure SF1 Bayesian Inference species tree topology. Supports noted above branches as BI/ML (BI=support from Bayesian inference, ML=support from Maximum Likelihood ultrafast bootstrap; * denotes full support, - denotes branch is missing from ML consensus). Numbers below tree nodes are induced by post-order traversal of the tree.

Supplementary Figure SF2 Maximum Likelihood species tree topology. Support noted above branches (* denotes full support).

Supplementary Figure SF3 Amalgamated gene tree with annotated exon boundaries and detected domain signatures (with supports, branches not drawn to scale). Supports noted below branches in form BI/ML/C (BI=support from Bayesian inference, ML=support from Maximum Likelihood ultrafast bootstrap, C=whether bipartition is present in Bayesian consensus tree; * denotes full support, % denotes support below 1% level). Domain architecture and gene structure are visualised on the right side (colors: red – SAT, blue – KS, green – AT, purple – PT, orange – ACP, pink – TE, brown – R). Color of the tree edges and letters denote predicted events (D – duplication, TD – transfer donated, TA – transfer accepted).

Supplementary Figure SF4 Example of statistical testing conducted to detect strongest, distinct relationships between monophyletic clades and presence/absence of candidate homolog. In the example, β-lactamases (key accessory enzyme for C6-C11 cyclisation) show strongest association with subtree (node) *g552*, where P-value (pre-Bonferroni correction; one-sided Fisher exact test) hits a distinct minimum. All such non-overlapping minima, scoring above 0.0001 post-correction were further analysed (see Table 2). Notation used: *g* – gene (core NR-PKS), *S* – subtree, *chg* – candidate homolog group member gene.

Supplementary Table ST1. Model genomes used in the phylogenomic analysis. Asterisk denotes genome assemblies which have been available via GenBank for at least 1 year without formal publication.

**Taxon**	**Data source**	**Reference**
Agaricus bisporus var. bisporus H97	JGI/DOE	(Morin et al. 2012)
Agaricus bisporus var. burnettii JB137 S8	JGI/DOE	(Morin et al. 2012)
Ajellomyces capsulatus NAm1	NCBI	(Sharpton et al. 2009)
Ajellomyces dermatitidis SLH14081	NCBI	*
Arthrobotrys oligospora ATCC 24927	NCBI	(Yang et al. 2011)
Arthroderma benhamiae CBS 112371	NCBI	*
Arthroderma otae CBS 113480	NCBI	(Martinez et al. 2012)
Ashbya gossypii ATCC 10895	NCBI	(Dietrich et al. 2004)
Aspergillus clavatus NRRL 1	NCBI	(Fedorova et al. 2008)
Aspergillus flavus NRRL3357	Ensembl	(Mabey Gilsenan et al. 2012)
Aspergillus fumigatus A1163	Ensembl	(Fedorova et al. 2008; Mabey Gilsenan et al. 2012)
Aspergillus fumigatus Af293	Ensembl	(Nierman et al. 2005)
Aspergillus kawachii IFO 4308	NCBI	(Futagami et al. 2011)
Aspergillus nidulans FGSC A4	Ensembl	(Galagan et al. 2005; Wortman et al. 2009)
Aspergillus niger ATCC 1015	NCBI	(Andersen et al. 2011)
Aspergillus niger CBS 513.88	NCBI	(Pel et al. 2007)
Aspergillus oryzae RIB40	Ensembl	(Machida et al. 2005)
Aspergillus terreus NIH2624	Ensembl	(Mabey Gilsenan et al. 2012)
Auricularia delicata TFB 10046 SS5	NCBI	(Floudas et al. 2012)
Batrachochytrium dendrobatidis JAM81	NCBI	*
Baudoinia compniacensis UAMH 10762	JGI/DOE	(Ohm et al. 2012)
Beauveria bassiana ARSEF 2860	NCBI	(Xiao et al. 2012)
Bipolaris maydis ATCC 48331	JGI/DOE	(Ohm et al. 2012)
Bipolaris maydis C5	JGI/DOE	(Ohm et al. 2012)
Bipolaris sorokiniana ND90Pr	JGI/DOE	(Ohm et al. 2012)
Blumeria graminis f sp hordei DH14	BluGen	(Spanu et al. 2010)
Botrytis cinerea B05.10	NCBI	(Amselem et al. 2011)
Caenorhabditis elegans	NCBI	(C. elegans Sequencing Consortium

		1998)
Candida albicans WO 1	NCBI	(Butler et al. 2009)
Candida dubliniensis CD36	NCBI	(Jackson et al. 2009)
Candida glabrata CBS 138	NCBI	(Dujon et al. 2004)
Candida tenuis ATCC 10573	NCBI	(Wohlbach et al. 2011)
Candida tropicalis MYA 3404	NCBI	(Butler et al. 2009)
Ceriporiopsis subvermispora B	JGI/DOE	(Fernandez-Fueyo et al. 2012)
Chaetomium globosum CBS 148.51	NCBI	*
Chaetomium thermophilum var. thermophilum DSM 1495	NCBI	(Amlacher et al. 2011)
Clavispora lusitaniae ATCC 42720	NCBI	(Butler et al. 2009)
Coccidioides immitis RS	NCBI	(Sharpton et al. 2009)
Coccidioides posadasii C735 delta SOWgp	NCBI	(Sharpton et al. 2009)
Coccidioides posadasii str. Silveira	NCBI	(Sharpton et al. 2009)
Colletotrichum graminicola M1.001	NCBI	(O'Connell et al. 2012)
Colletotrichum higginsianum	NCBI	(O'Connell et al. 2012)
Coniophora puteana RWD 64 598 SS2	NCBI	(Floudas et al. 2012)
Coprinopsis cinerea okayama7 130	NCBI	(Stajich et al. 2010)
Cordyceps militaris CM01	NCBI	(Zheng et al. 2011)
Cryptococcus gattii WM276	NCBI	(D'Souza et al. 2011)
Cryptococcus neoformans var. neoformans B 3501A	NCBI	*
Cryptococcus neoformans var. neoformans JEC21	NCBI	(Loftus et al. 2005)
Dacryopinax sp. DJM 731 SS1	NCBI	(Floudas et al. 2012)
Debaryomyces hansenii CBS767	NCBI	(Dujon et al. 2004)
Dekkera bruxellensis AWRI1499	NCBI	(Curtin et al. 2012)
Dichomitus squalens LYAD 421 SS1	NCBI	(Floudas et al. 2012)
Dothistroma septosporum NZE10	JGI/DOE	(Ohm et al. 2012)
Eremothecium cymbalariae DBVPG 7215	NCBI	(Wendland and Walther 2011)
Exophiala dermatitidis NIH/UT8656	NCBI	*
Fomitiporia mediterranea MF3/22	NCBI	(Floudas et al. 2012)
Fusarium graminearum PH 1	Ensembl	(Cuomo et al. 2007)
Fusarium oxysporum f. sp. lycopersici 4287	Ensembl	(Ma et al. 2010)
Fusarium oxysporum Fo5176	NCBI	(Thatcher et al. 2012)

Fusarium pseudograminearum CS3096	NCBI	(Gardiner et al. 2012)
Fusarium verticillioides 7600	Ensembl	(Ma et al. 2010)
Gaeumannomyces graminis var. tritici R3 111a 1	Ensembl	*[1]
Glarea lozoyensis 74030	NCBI	(Youssar et al. 2012)
Grosmannia clavigera kw1407	NCBI	(DiGuistini et al. 2011)
Hysterium pulicare CBS 123377	JGI/DOE	(Ohm et al. 2012)
Kazachstania africana CBS 2517	NCBI	(Gordon et al. 2011)
Kluyveromyces lactis NRRL Y 1140	NCBI	(Dujon et al. 2004)
Komagataella pastoris GS115	NCBI	(De Schutter et al. 2009)
Laccaria bicolor S238N H82	NCBI	(Martin et al. 2008)
Lachancea thermotolerans CBS 6340	NCBI	(Génolevures Consortium et al. 2009)
Leptosphaeria maculans JN3	NCBI	(Rouxel et al. 2011)
Lodderomyces elongisporus NRRL YB 4239	NCBI	(Butler et al. 2009)
Magnaporthe oryzae	Ensembl	(Dean et al. 2005)
Magnaporthiopsis poae ATCC 64411	Ensembl	*1
Malassezia globosa CBS 7966	NCBI	(Xu et al. 2007)
Marssonina brunnea f. sp. 'multigermtubi' MB m1	NCBI	(Zhu et al. 2012)
Melampsora laricis-populina 98AG31	NCBI	(Duplessis et al. 2011)
Metarhizium acridum CQMa 102	NCBI	(Gao et al. 2011)
Metarhizium robertsii ARSEF 23	NCBI	(Gao et al. 2011)
Meyerozyma guilliermondii ATCC 6260	NCBI	(Butler et al. 2009)
Microsporum gypseum CBS 118893	NCBI	(Martinez et al. 2012)
Mixia osmundae IAM 14324	NCBI	(Nishida et al. 2011)
Moniliophthora perniciosa FA553	NCBI	(Mondego et al. 2008)
Myceliophthora thermophila ATCC 42464	NCBI	(Berka et al. 2011)
Naumovozyma castellii CBS 4309	NCBI	(Gordon et al. 2011)
Naumovozyma dairenensis CBS 421	NCBI	(Gordon et al. 2011)
Nectria haematococca mpVI 77 13 4	Ensembl	(Coleman et al. 2009)
Neosartorya fischeri NRRL 181	NCBI	(Fedorova et al. 2008)
Neurospora crassa OR74A	NCBI	(Galagan et al. 2003)
Neurospora tetrasperma FGSC 2508	NCBI	(Ellison et al. 2011)
Ogataea parapolymorpha DL 1	NCBI	(Ramezani-Rad et al. 2003)
Paracoccidioides brasiliensis Pb18	NCBI	(Desjardins et al. 2011)
Paracoccidioides sp. 'lutzii' Pb01	NCBI	(Desjardins et al. 2011)

Passalora fulva CBS 131901	JGI/DOE	(Ohm et al. 2012)
Penicillium rubens Wisconsin 54 1255	NCBI	(Nierman et al. 2005)
Phaeosphaeria nodorum SN15	NCBI	(Hane et al. 2007)
Phanerochaete carnosa HHB 10118 sp	JGI/DOE	(Fernandez-Fueyo et al. 2012)
Piriformospora indica DSM 11827	NCBI	(Zuccaro et al. 2011)
Podospora anserina S mat+	NCBI	(Paoletti and Saupe 2008)
Postia placenta Mad 698 R	NCBI	(Martinez et al. 2009)
Pseudocercospora fijiensis CIRAD86	JGI/DOE	(Ohm et al. 2012)
Puccinia graminis f. sp. tritici CRL 75 36 700 3	NCBI	(Duplessis et al. 2011)
Puccinia triticina 1 1 BBBD Race 1	Ensembl	(Duplessis et al. 2011)
Punctularia strigosozonata HHB 11173 SS5	NCBI	(Floudas et al. 2012)
Pyrenophora teres f. teres 0 1	NCBI	(Ellwood et al. 2010)
Pyrenophora tritici-repentis Pt-1C-BFP	NCBI	*
Rhizopus delemar RA 99 880	NCBI	(Ma et al. 2009)
Rhodotorula glutinis ATCC 204091	NCBI	*
Rhytidhysteron rufulum CBS 306 38	JGI/DOE	(Ohm et al. 2012)
Saccharomyces cerevisiae S288c	NCBI	(Goffeau et al. 1996)
Scheffersomyces stipitis CBS 6054	NCBI	(Jeffries et al. 2007)
Schizophyllum commune H4 8	NCBI	(Ohm et al. 2010)
Schizosaccharomyces japonicus yFS275	NCBI	(Rhind et al. 2011)
Schizosaccharomyces pombe 972h	NCBI	(Wood et al. 2002)
Sclerotinia sclerotiorum 1980 UF 70	NCBI	(Amselem et al. 2011)
Serpula lacrymans var. lacrymans S7.3	NCBI	(Eastwood et al. 2011)
Serpula lacrymans var. lacrymans S7.9	NCBI	(Eastwood et al. 2011)
Setosphaeria turcica Et28A	JGI/DOE	(Ohm et al. 2012)
Sordaria macrospora k hell	NCBI	(Nowrousian et al. 2010)
Spathaspora passalidarum NRRL Y 27907	NCBI	(Wohlbach et al. 2011)
Sphaerulina musiva SO2202	JGI/DOE	(Ohm et al. 2012)
Sphaerulina populicola P02.02b	JGI/DOE	(Ohm et al. 2012)
Sporisorium reilianum SRZ2	NCBI	(Schirawski et al. 2010)
Stereum hirsutum FP 91666 SS1	NCBI	(Floudas et al. 2012)
Talaromyces marneffei ATCC 18224	NCBI	*
Talaromyces stipitatus ATCC 10500	NCBI	*
Tetrapisispora blattae CBS 6284	NCBI	(Gordon et al. 2011)
Tetrapisispora phaffii CBS 4417	NCBI	(Gordon et al. 2011)

Thielavia terrestris NRRL 8126	NCBI	(Berka et al. 2011)
Torulaspora delbrueckii	NCBI	(Gordon et al. 2011)
Trametes versicolor FP 101664 SS1	NCBI	(Floudas et al. 2012)
Tremella mesenterica DSM 1558	NCBI	(Floudas et al. 2012)
Trichoderma atroviride IMI 206040	NCBI	(Kubicek et al. 2011)
Trichoderma reesei QM6a	NCBI	(Martinez et al. 2008)
Trichoderma virens Gv29 8	NCBI	(Kubicek et al. 2011)
Trichophyton rubrum CBS 118892	NCBI	(Martinez et al. 2012)
Trichophyton tonsurans CBS 112818	NCBI	(Martinez et al. 2012)
Trichophyton verrucosum HKI 0517	NCBI	(Martinez et al. 2012)
Trichosporon asahii var. asahii CBS 8904	NCBI	(Yang et al. 2012)
Tuber melanosporum Mel28	NCBI	(Martin et al. 2010)
Uncinocarpus reesii 1704	NCBI	(Sharpton et al. 2009)
Ustilago hordei	NCBI	(Laurie et al. 2012)
Ustilago maydis 521	NCBI	(Kämper et al. 2006)
Vanderwaltozyma polyspora DSM 70294	NCBI	(Scannell et al. 2007)
Verticillium alfalfae VaMs.102	NCBI	(Klosterman et al. 2011)
Verticillium dahliae VdLs.17	NCBI	(Klosterman et al. 2011)
Wallemia sebi CBS 633.66	NCBI	(Padamsee et al. 2012)
Yarrowia lipolytica CLIB122	NCBI	(Dujon et al. 2004)
Zygosaccharomyces rouxii CBS 732	NCBI	(Génolevures Consortium et al. 2009)
Zymoseptoria tritici IPO323	NCBI	(Goodwin et al. 2011)

1 - *Magnaporthe* Comparative Sequencing Project, Broad Institute of Harvard and MIT (http://www.broadinstitute.org/),

* - Draft genome available on NCBI/RefSeq or Ensembl/Fungi, for a period over 12 months, without traceable genome announcement publication.

References:

Amlacher S, Sarges P, Flemming D, van Noort V, Kunze R, Devos DP, Arumugam M, Bork P, Hurt E. 2011. Insight into structure and assembly of the nuclear pore complex by utilizing the genome of a eukaryotic thermophile. Cell 146:277–289.

Amselem J, Cuomo CA, van Kan JAL, Viaud M, Benito EP, Couloux A, Coutinho PM, de Vries RP, Dyer PS, Fillinger S, et al. 2011. Genomic analysis of the necrotrophic fungal pathogens Sclerotinia sclerotiorum and Botrytis cinerea. PLoS Genet. 7:e1002230.

Andersen MR, Salazar MP, Schaap PJ, van de Vondervoort PJI, Culley D, Thykaer J, Frisvad JC, Nielsen KF, Albang R, Albermann K, et al. 2011. Comparative genomics of citric-acid-producing Aspergillus niger ATCC 1015 versus enzyme-producing CBS 513.88. Genome Res. 21:885–897.

Berka RM, Grigoriev IV, Otillar R, Salamov A, Grimwood J, Reid I, Ishmael N, John T, Darmond C, Moisan M-C, et al. 2011. Comparative genomic analysis of the thermophilic biomass-degrading fungi Myceliophthora thermophila and Thielavia terrestris. Nat. Biotechnol. 29:922–927.

Butler G, Rasmussen MD, Lin MF, Santos MAS, Sakthikumar S, Munro CA, Rheinbay E, Grabherr M, Forche A, Reedy JL, et al. 2009. Evolution of pathogenicity and sexual reproduction in eight Candida genomes. Nature 459:657–662.

C. elegans Sequencing Consortium. 1998. Genome sequence of the nematode C. elegans: a platform for investigating biology. Science 282:2012–2018.

Coleman JJ, Rounsley SD, Rodriguez-Carres M, Kuo A, Wasmann CC, Grimwood J, Schmutz J, Taga M, White GJ, Zhou S, et al. 2009. The genome of Nectria haematococca: contribution of supernumerary chromosomes to gene expansion. PLoS Genet. 5:e1000618.

Cuomo CA, Güldener U, Xu J-R, Trail F, Turgeon BG, Di Pietro A, Walton JD, Ma L-J, Baker SE, Rep M, et al. 2007. The Fusarium graminearum genome reveals a link between localized polymorphism and pathogen specialization. Science 317:1400–1402.

Curtin CD, Borneman AR, Chambers PJ, Pretorius IS. 2012. De-novo assembly and analysis of the heterozygous triploid genome of the wine spoilage yeast Dekkera bruxellensis AWRI1499. PloS One 7:e33840.

Dean RA, Talbot NJ, Ebbole DJ, Farman ML, Mitchell TK, Orbach MJ, Thon M, Kulkarni R, Xu J-R, Pan H, et al. 2005. The genome sequence of the rice blast fungus Magnaporthe grisea. Nature 434:980–986.

Desjardins CA, Champion MD, Holder JW, Muszewska A, Goldberg J, Bailão AM, Brigido MM, Ferreira ME da S, Garcia AM, Grynberg M, et al. 2011. Comparative genomic analysis of human fungal pathogens causing paracoccidioidomycosis. PLoS Genet. 7:e1002345.

Dietrich FS, Voegeli S, Brachat S, Lerch A, Gates K, Steiner S, Mohr C, Pöhlmann R, Luedi P, Choi S, et al. 2004. The Ashbya gossypii genome as a tool for mapping the ancient Saccharomyces cerevisiae genome. Science 304:304–307.

DiGuistini S, Wang Y, Liao NY, Taylor G, Tanguay P, Feau N, Henrissat B, Chan SK, Hesse-Orce U, Alamouti SM, et al. 2011. Genome and transcriptome analyses of the mountain pine beetle-fungal symbiont Grosmannia clavigera, a lodgepole pine pathogen. Proc. Natl. Acad. Sci. U. S. A. 108:2504–2509.

D'Souza CA, Kronstad JW, Taylor G, Warren R, Yuen M, Hu G, Jung WH, Sham A, Kidd SE, Tangen K, et al. 2011. Genome variation in Cryptococcus gattii, an emerging pathogen of immunocompetent hosts. mBio 2:e00342–00310.

Dujon B, Sherman D, Fischer G, Durrens P, Casaregola S, Lafontaine I, De Montigny J, Marck C, Neuvéglise C, Talla E, et al. 2004. Genome evolution in yeasts. Nature 430:35–44.

Duplessis S, Cuomo CA, Lin Y-C, Aerts A, Tisserant E, Veneault-Fourrey C, Joly DL, Hacquard S, Amselem J, Cantarel BL, et al. 2011. Obligate biotrophy features unraveled by the genomic analysis of rust fungi. Proc. Natl. Acad. Sci. 108:9166–9171.

Eastwood DC, Floudas D, Binder M, Majcherczyk A, Schneider P, Aerts A, Asiegbu FO, Baker SE, Barry K, Bendiksby M, et al. 2011. The plant cell wall-decomposing machinery underlies the functional diversity of forest fungi. Science 333:762–765.

Ellison CE, Stajich JE, Jacobson DJ, Natvig DO, Lapidus A, Foster B, Aerts A, Riley R, Lindquist EA, Grigoriev IV, et al. 2011. Massive changes in genome architecture accompany the transition to self-fertility in the filamentous fungus Neurospora tetrasperma. Genetics 189:55–69.

Ellwood SR, Liu Z, Syme RA, Lai Z, Hane JK, Keiper F, Moffat CS, Oliver RP, Friesen TL. 2010. A first genome assembly of the barley fungal pathogen Pyrenophora teres f. teres. Genome Biol. 11:R109.

Fedorova ND, Khaldi N, Joardar VS, Maiti R, Amedeo P, Anderson MJ, Crabtree J, Silva JC, Badger JH, Albarraq A, et al. 2008. Genomic islands in the pathogenic filamentous fungus Aspergillus fumigatus. PLoS Genet. 4:e1000046.

Fernandez-Fueyo E, Ruiz-Dueñas FJ, Ferreira P, Floudas D, Hibbett DS, Canessa P, Larrondo LF, James TY, Seelenfreund D, Lobos S, et al. 2012. Comparative genomics of Ceriporiopsis subvermispora and Phanerochaete chrysosporium provide insight into selective ligninolysis. Proc. Natl. Acad. Sci. U. S. A. 109:5458–5463.

Floudas D, Binder M, Riley R, Barry K, Blanchette RA, Henrissat B, Martínez AT, Otillar R, Spatafora JW, Yadav JS, et al. 2012. The Paleozoic origin of enzymatic lignin decomposition reconstructed from 31 fungal genomes. Science 336:1715–1719.

Futagami T, Mori K, Yamashita A, Wada S, Kajiwara Y, Takashita H, Omori T, Takegawa K, Tashiro K, Kuhara S, et al. 2011. Genome sequence of the white koji mold Aspergillus kawachii IFO 4308, used for brewing the Japanese distilled spirit shochu. Eukaryot. Cell 10:1586–1587.

Galagan JE, Calvo SE, Borkovich KA, Selker EU, Read ND, Jaffe D, FitzHugh W, Ma L-J, Smirnov S, Purcell S, et al. 2003. The genome sequence of the filamentous fungus Neurospora crassa. Nature 422:859–868.

Galagan JE, Calvo SE, Cuomo C, Ma L-J, Wortman JR, Batzoglou S, Lee S-I, Baştürkmen M, Spevak

CC, Clutterbuck J, et al. 2005. Sequencing of Aspergillus nidulans and comparative analysis with A. fumigatus and A. oryzae. Nature 438:1105–1115.

Gao Q, Jin K, Ying S-H, Zhang Y, Xiao G, Shang Y, Duan Z, Hu X, Xie X-Q, Zhou G, et al. 2011. Genome sequencing and comparative transcriptomics of the model entomopathogenic fungi Metarhizium anisopliae and M. acridum. PLoS Genet. 7:e1001264.

Gardiner DM, McDonald MC, Covarelli L, Solomon PS, Rusu AG, Marshall M, Kazan K, Chakraborty S, McDonald BA, Manners JM. 2012. Comparative pathogenomics reveals horizontally acquired novel virulence genes in fungi infecting cereal hosts. PLoS Pathog. 8:e1002952.

Génolevures Consortium, Souciet J-L, Dujon B, Gaillardin C, Johnston M, Baret PV, Cliften P, Sherman DJ, Weissenbach J, Westhof E, et al. 2009. Comparative genomics of protoploid Saccharomycetaceae. Genome Res. 19:1696–1709.

Goffeau A, Barrell BG, Bussey H, Davis RW, Dujon B, Feldmann H, Galibert F, Hoheisel JD, Jacq C, Johnston M, et al. 1996. Life with 6000 genes. Science 274:546, 563–567.

Goodwin SB, M'barek SB, Dhillon B, Wittenberg AHJ, Crane CF, Hane JK, Foster AJ, Van der Lee TAJ, Grimwood J, Aerts A, et al. 2011. Finished genome of the fungal wheat pathogen Mycosphaerella graminicola reveals dispensome structure, chromosome plasticity, and stealth pathogenesis. PLoS Genet. 7:e1002070.

Gordon JL, Armisén D, Proux-Wéra E, ÓhÉigeartaigh SS, Byrne KP, Wolfe KH. 2011. Evolutionary erosion of yeast sex chromosomes by mating-type switching accidents. Proc. Natl. Acad. Sci. U. S. A. 108:20024–20029.

Hane JK, Lowe RGT, Solomon PS, Tan K-C, Schoch CL, Spatafora JW, Crous PW, Kodira C, Birren BW, Galagan JE, et al. 2007. Dothideomycete plant interactions illuminated by genome sequencing and EST analysis of the wheat pathogen Stagonospora nodorum. Plant Cell 19:3347–3368.

Jackson AP, Gamble JA, Yeomans T, Moran GP, Saunders D, Harris D, Aslett M, Barrell JF, Butler G, Citiulo F, et al. 2009. Comparative genomics of the fungal pathogens Candida dubliniensis and Candida albicans. Genome Res. 19:2231–2244.

Jeffries TW, Grigoriev IV, Grimwood J, Laplaza JM, Aerts A, Salamov A, Schmutz J, Lindquist E, Dehal P, Shapiro H, et al. 2007. Genome sequence of the lignocellulose-bioconverting and xylose-fermenting yeast Pichia stipitis. Nat. Biotechnol. 25:319–326.

Kämper J, Kahmann R, Bölker M, Ma L-J, Brefort T, Saville BJ, Banuett F, Kronstad JW, Gold SE, Müller O, et al. 2006. Insights from the genome of the biotrophic fungal plant pathogen Ustilago maydis. Nature 444:97–101.

Klosterman SJ, Subbarao KV, Kang S, Veronese P, Gold SE, Thomma BPHJ, Chen Z, Henrissat B, Lee Y-H, Park J, et al. 2011. Comparative genomics yields insights into niche adaptation of plant vascular wilt pathogens. PLoS Pathog. 7:e1002137.

Kubicek CP, Herrera-Estrella A, Seidl-Seiboth V, Martinez DA, Druzhinina IS, Thon M, Zeilinger S, Casas-Flores S, Horwitz BA, Mukherjee PK, et al. 2011. Comparative genome sequence

analysis underscores mycoparasitism as the ancestral life style of Trichoderma. Genome Biol. 12:R40.

Laurie JD, Ali S, Linning R, Mannhaupt G, Wong P, Güldener U, Münsterkötter M, Moore R, Kahmann R, Bakkeren G, et al. 2012. Genome comparison of barley and maize smut fungi reveals targeted loss of RNA silencing components and species-specific presence of transposable elements. Plant Cell 24:1733–1745.

Loftus BJ, Fung E, Roncaglia P, Rowley D, Amedeo P, Bruno D, Vamathevan J, Miranda M, Anderson IJ, Fraser JA, et al. 2005. The genome of the basidiomycetous yeast and human pathogen Cryptococcus neoformans. Science 307:1321–1324.

Mabey Gilsenan J, Cooley J, Bowyer P. 2012. CADRE: the Central Aspergillus Data REpository 2012. Nucleic Acids Res. 40:D660–D666.

Machida M, Asai K, Sano M, Tanaka T, Kumagai T, Terai G, Kusumoto K-I, Arima T, Akita O, Kashiwagi Y, et al. 2005. Genome sequencing and analysis of Aspergillus oryzae. Nature 438:1157–1161.

Ma L-J, van der Does HC, Borkovich KA, Coleman JJ, Daboussi M-J, Di Pietro A, Dufresne M, Freitag M, Grabherr M, Henrissat B, et al. 2010. Comparative genomics reveals mobile pathogenicity chromosomes in Fusarium. Nature 464:367–373.

Ma L-J, Ibrahim AS, Skory C, Grabherr MG, Burger G, Butler M, Elias M, Idnurm A, Lang BF, Sone T, et al. 2009. Genomic analysis of the basal lineage fungus Rhizopus oryzae reveals a whole-genome duplication. PLoS Genet. 5:e1000549.

Martinez DA, Oliver BG, Gräser Y, Goldberg JM, Li W, Martinez-Rossi NM, Monod M, Shelest E, Barton RC, Birch E, et al. 2012. Comparative genome analysis of Trichophyton rubrum and related dermatophytes reveals candidate genes involved in infection. mBio 3:e00259–00212.

Martinez D, Berka RM, Henrissat B, Saloheimo M, Arvas M, Baker SE, Chapman J, Chertkov O, Coutinho PM, Cullen D, et al. 2008. Genome sequencing and analysis of the biomass-degrading fungus Trichoderma reesei (syn. Hypocrea jecorina). Nat. Biotechnol. 26:553–560.

Martinez D, Challacombe J, Morgenstern I, Hibbett D, Schmoll M, Kubicek CP, Ferreira P, Ruiz-Duenas FJ, Martinez AT, Kersten P, et al. 2009. Genome, transcriptome, and secretome analysis of wood decay fungus Postia placenta supports unique mechanisms of lignocellulose conversion. Proc. Natl. Acad. Sci. U. S. A. 106:1954–1959.

Martin F, Aerts A, Ahrén D, Brun A, Danchin EGJ, Duchaussoy F, Gibon J, Kohler A, Lindquist E, Pereda V, et al. 2008. The genome of Laccaria bicolor provides insights into mycorrhizal symbiosis. Nature 452:88–92.

Martin F, Kohler A, Murat C, Balestrini R, Coutinho PM, Jaillon O, Montanini B, Morin E, Noel B, Percudani R, et al. 2010. Périgord black truffle genome uncovers evolutionary origins and mechanisms of symbiosis. Nature 464:1033–1038.

Mondego JMC, Carazzolle MF, Costa GGL, Formighieri EF, Parizzi LP, Rincones J, Cotomacci C, Carraro DM, Cunha AF, Carrer H, et al. 2008. A genome survey of Moniliophthora perniciosa

gives new insights into Witches' Broom Disease of cacao. BMC Genomics 9:548.

Morin E, Kohler A, Baker AR, Foulongne-Oriol M, Lombard V, Nagy LG, Ohm RA, Patyshakuliyeva A, Brun A, Aerts AL, et al. 2012. Genome sequence of the button mushroom Agaricus bisporus reveals mechanisms governing adaptation to a humic-rich ecological niche. Proc. Natl. Acad. Sci. U. S. A. 109:17501–17506.

Nierman WC, Pain A, Anderson MJ, Wortman JR, Kim HS, Arroyo J, Berriman M, Abe K, Archer DB, Bermejo C, et al. 2005. Genomic sequence of the pathogenic and allergenic filamentous fungus Aspergillus fumigatus. Nature 438:1151–1156.

Nishida H, Nagatsuka Y, Sugiyama J. 2011. Draft genome sequencing of the enigmatic basidiomycete Mixia osmundae. J. Gen. Appl. Microbiol. 57:63–67.

Nowrousian M, Stajich JE, Chu M, Engh I, Espagne E, Halliday K, Kamerewerd J, Kempken F, Knab B, Kuo H-C, et al. 2010. De novo assembly of a 40 Mb eukaryotic genome from short sequence reads: Sordaria macrospora, a model organism for fungal morphogenesis. PLoS Genet. 6:e1000891.

O'Connell RJ, Thon MR, Hacquard S, Amyotte SG, Kleemann J, Torres MF, Damm U, Buiate EA, Epstein L, Alkan N, et al. 2012. Lifestyle transitions in plant pathogenic Colletotrichum fungi deciphered by genome and transcriptome analyses. Nat. Genet. [Internet]. Available from: http://www.ncbi.nlm.nih.gov/pubmed/22885923

Ohm RA, Feau N, Henrissat B, Schoch CL, Horwitz BA, Barry KW, Condon BJ, Copeland AC, Dhillon B, Glaser F, et al. 2012. Diverse lifestyles and strategies of plant pathogenesis encoded in the genomes of eighteen Dothideomycetes fungi. PLoS Pathog. 8:e1003037.

Ohm RA, de Jong JF, Lugones LG, Aerts A, Kothe E, Stajich JE, de Vries RP, Record E, Levasseur A, Baker SE, et al. 2010. Genome sequence of the model mushroom Schizophyllum commune. Nat. Biotechnol. 28:957–963.

Padamsee M, Kumar TKA, Riley R, Binder M, Boyd A, Calvo AM, Furukawa K, Hesse C, Hohmann S, James TY, et al. 2012. The genome of the xerotolerant mold Wallemia sebi reveals adaptations to osmotic stress and suggests cryptic sexual reproduction. Fungal Genet. Biol. FG B 49:217–226.

Paoletti M, Saupe SJ. 2008. The genome sequence of Podospora anserina, a classic model fungus. Genome Biol. 9:223.

Pel HJ, de Winde JH, Archer DB, Dyer PS, Hofmann G, Schaap PJ, Turner G, de Vries RP, Albang R, Albermann K, et al. 2007. Genome sequencing and analysis of the versatile cell factory Aspergillus niger CBS 513.88. Nat. Biotechnol. 25:221–231.

Ramezani-Rad M, Hollenberg CP, Lauber J, Wedler H, Griess E, Wagner C, Albermann K, Hani J, Piontek M, Dahlems U, et al. 2003. The Hansenula polymorpha (strain CBS4732) genome sequencing and analysis. FEMS Yeast Res. 4:207–215.

Rhind N, Chen Z, Yassour M, Thompson DA, Haas BJ, Habib N, Wapinski I, Roy S, Lin MF, Heiman DI, et al. 2011. Comparative functional genomics of the fission yeasts. Science 332:930–936.

Rouxel T, Grandaubert J, Hane JK, Hoede C, van de Wouw AP, Couloux A, Dominguez V, Anthouard V, Bally P, Bourras S, et al. 2011. Effector diversification within compartments of the Leptosphaeria maculans genome affected by Repeat-Induced Point mutations. Nat. Commun. 2:202.

Scannell DR, Frank AC, Conant GC, Byrne KP, Woolfit M, Wolfe KH. 2007. Independent sorting-out of thousands of duplicated gene pairs in two yeast species descended from a whole-genome duplication. Proc. Natl. Acad. Sci. U. S. A. 104:8397–8402.

Schirawski J, Mannhaupt G, Münch K, Brefort T, Schipper K, Doehlemann G, Di Stasio M, Rössel N, Mendoza-Mendoza A, Pester D, et al. 2010. Pathogenicity determinants in smut fungi revealed by genome comparison. Science 330:1546–1548.

De Schutter K, Lin Y-C, Tiels P, Van Hecke A, Glinka S, Weber-Lehmann J, Rouzé P, Van de Peer Y, Callewaert N. 2009. Genome sequence of the recombinant protein production host Pichia pastoris. Nat. Biotechnol. 27:561–566.

Sharpton TJ, Stajich JE, Rounsley SD, Gardner MJ, Wortman JR, Jordar VS, Maiti R, Kodira CD, Neafsey DE, Zeng Q, et al. 2009. Comparative genomic analyses of the human fungal pathogens Coccidioides and their relatives. Genome Res. 19:1722–1731.

Spanu PD, Abbott JC, Amselem J, Burgis TA, Soanes DM, Stüber K, Ver Loren van Themaat E, Brown JKM, Butcher SA, Gurr SJ, et al. 2010. Genome expansion and gene loss in powdery mildew fungi reveal tradeoffs in extreme parasitism. Science 330:1543–1546.

Stajich JE, Wilke SK, Ahrén D, Au CH, Birren BW, Borodovsky M, Burns C, Canbäck B, Casselton LA, Cheng CK, et al. 2010. Insights into evolution of multicellular fungi from the assembled chromosomes of the mushroom Coprinopsis cinerea (Coprinus cinereus). Proc. Natl. Acad. Sci. U. S. A. 107:11889–11894.

Thatcher LF, Gardiner DM, Kazan K, Manners JM. 2012. A highly conserved effector in Fusarium oxysporum is required for full virulence on Arabidopsis. Mol. Plant-Microbe Interact. MPMI 25:180–190.

Wendland J, Walther A. 2011. Genome evolution in the eremothecium clade of the Saccharomyces complex revealed by comparative genomics. G3 Bethesda Md 1:539–548.

Wohlbach DJ, Kuo A, Sato TK, Potts KM, Salamov AA, Labutti KM, Sun H, Clum A, Pangilinan JL, Lindquist EA, et al. 2011. Comparative genomics of xylose-fermenting fungi for enhanced biofuel production. Proc. Natl. Acad. Sci. U. S. A. 108:13212–13217.

Wood V, Gwilliam R, Rajandream M-A, Lyne M, Lyne R, Stewart A, Sgouros J, Peat N, Hayles J, Baker S, et al. 2002. The genome sequence of Schizosaccharomyces pombe. Nature 415:871–880.

Wortman JR, Gilsenan JM, Joardar V, Deegan J, Clutterbuck J, Andersen MR, Archer D, Bencina M, Braus G, Coutinho P, et al. 2009. The 2008 update of the Aspergillus nidulans genome annotation: a community effort. Fungal Genet. Biol. FG B 46 Suppl 1:S2–S13.

Xiao G, Ying S-H, Zheng P, Wang Z-L, Zhang S, Xie X-Q, Shang Y, St Leger RJ, Zhao G-P, Wang C,

et al. 2012. Genomic perspectives on the evolution of fungal entomopathogenicity in Beauveria bassiana. Sci. Rep. 2:483.

Xu J, Saunders CW, Hu P, Grant RA, Boekhout T, Kuramae EE, Kronstad JW, Deangelis YM, Reeder NL, Johnstone KR, et al. 2007. Dandruff-associated Malassezia genomes reveal convergent and divergent virulence traits shared with plant and human fungal pathogens. Proc. Natl. Acad. Sci. U. S. A. 104:18730–18735.

Yang J, Wang L, Ji X, Feng Y, Li X, Zou C, Xu J, Ren Y, Mi Q, Wu J, et al. 2011. Genomic and proteomic analyses of the fungus Arthrobotrys oligospora provide insights into nematode-trap formation. PLoS Pathog. 7:e1002179.

Yang RY, Li HT, Zhu H, Zhou GP, Wang M, Wang L. 2012. Genome sequence of the Trichosporon asahii environmental strain CBS 8904. Eukaryot. Cell 11:1586–1587.

Youssar L, Grüning BA, Erxleben A, Günther S, Hüttel W. 2012. Genome sequence of the fungus Glarea lozoyensis: the first genome sequence of a species from the Helotiaceae family. Eukaryot. Cell 11:250.

Zheng P, Xia Y, Xiao G, Xiong C, Hu X, Zhang S, Zheng H, Huang Y, Zhou Y, Wang S, et al. 2011. Genome sequence of the insect pathogenic fungus Cordyceps militaris, a valued traditional Chinese medicine. Genome Biol. 12:R116.

Zhu S, Cao Y-Z, Jiang C, Tan B-Y, Wang Z, Feng S, Zhang L, Su X-H, Brejova B, Vinar T, et al. 2012. Sequencing the genome of Marssonina brunnea reveals fungus-poplar co-evolution. BMC Genomics 13:382.

Zuccaro A, Lahrmann U, Güldener U, Langen G, Pfiffi S, Biedenkopf D, Wong P, Samans B, Grimm C, Basiewicz M, et al. 2011. Endophytic life strategies decoded by genome and transcriptome analyses of the mutualistic root symbiont Piriformospora indica. PLoS Pathog. 7:e1002290.

Supplementary Table ST2. Single-copy, orthologous, housekeeping genes chosen for species tree reconstruction. Choice was made on basis of topological score over 90% (as reported in FUNYBASE (Aguileta et al. 2008; Marthey et al. 2008)).

**Gene name**	**Over 95%**	***Saccharomyces cerevisiae* S288c GI** **(for reference protein sequence)**	***Saccharomyces cerevisiae* S288c accession** **(for reference protein sequence)**	**Optimal model** **(according to BIC)**	**Description**
Cct8p	Y	6322452	NP_012526	LG+I+G	T-complex protein 1 subunit theta; molecular chaperone
Crm1p	Y	398366207	NP_011734	LG+I+G	chromosome region maintenance protein 1; exportin 1; acts as receptor for the leucine-rich nuclear export signal
Elp3p	Y	6325171	NP_015239	LG+I+G	elongator complex protein 3; acts during transcription, as catalytic subunit of the RNA polymerase II elongator complex
Erb1p	Y	6323693	NP_013764	LG+I+G	eukaryotic ribosome biogenesis protein 1; component of NOP7 complex required for rRNA maturation and formation of 60S subunit
Gdh2p	Y	6319986	NP_010066	LG+I+G	NAD-specific glutamate dehydrogenase; involved in glutamate degradation
Hsh49p	Y	6324895	NP_014964	LG+I+G	protein HSH49; possible SF-B3 like factor
Icp55p	Y	6320922	NP_011001	LG+I+G	intermediate cleaving peptidase 55; involved in stabilisation of mitochondrial proteome
Ils1p	Y	6319395	NP_009477	LG+I+G	isoleucine--tRNA ligase, cytoplasmic
Ilv2p	Y	6323755	NP_013826	LG+I+G	acetolactate synthase catalytic subunit, mitochondrial; involved in amino acid biosynthesis (valine and isoleucine)
Kog1p	Y	6321980	NP_012056	LG+I+G+F	target of rapamycin complex 1 subunit KOG1; kontroller of growth

					protein 1; part of TORC1 complex 1 involved in control of cellular growth in response to environmental stimuli
Mcm7p	Y	398365471	NP_009761	LG+I+G	DNA replication licensing factor MCM7; minichromosome maintenance protein 7
Msh3p	Y	157285763	NP_010016	LG+I+G	DNA mismatch repair protein MSH3; part of post-replicative DNA mismatch repair system
Nat3p	Y	31126970	NP_015456	LG+I+G	N-terminal acetyltransferase B complex catalytic subunit NAT3; involved in amino terminal acetylation of methionines
Nhx1p	Y	398366611	NP_010744	LG+I+G+F	endosomal/prevacuolar sodium/hydrogen exchanger; vacuolar protein sorting-associated protein 44
Pol1p	Y	398364843	NP_014297	LG+I+G	DNA polymerase I subunit A
Pwp2p	Y	6319903	NP_009984	LG+I+G+F	periodic tryptophan protein 2; U3 snoRNA-associated protein 1; required for bud site selection and cell separation, involved in nucleolar preprocessing of pre-18S RNA
Rvb1p	Y	6320396	NP_010476	LG+I+G	RuvB family ATP-dependent DNA helicase pontin; involved in chromatin remodelling complexes
Rvb2p	Y	6325021	NP_015089	LG+I+G	RuvB family ATP-dependent DNA helicase reptin; involved in chromatin remodelling complexes
Ssl2p	Y	6322048	NP_012123	LG+I+G	DNA repair helicase RAD25; supressor of stem loop mutation 2
Tif5p	Y	6325298	NP_015366	LG+I+G	eukaryotic translation initiation factor 5 (eIF5); GTP hydrolase
Tsr1p	Y	6320143	NP_010223	LG+I+G	ribosome biogenesis protein TSR1; required for transport of pre-40S molecules from the nucleus to cytoplasm
Uba1p	Y	6322639	NP_012712	LG+I+G+F	ubiquitin activating enzyme E1
Vma4p	Y	398366277	NP_014977	LG+I+G	vacuolar proton ATPase subunit E

					(V-ATPase component)

References:

Aguileta G, Marthey S, Chiapello H, Lebrun M-H, Rodolphe F, Fournier E, Gendrault-Jacquemard A, Giraud T. 2008. Assessing the performance of single-copy genes for recovering robust phylogenies. Syst. Biol. 57:613–627.

Marthey S, Aguileta G, Rodolphe F, Gendrault A, Giraud T, Fournier E, Lopez-Villavicencio M, Gautier A, Lebrun M-H, Chiapello H. 2008. FUNYBASE: a FUNgal phYlogenomic dataBASE. BMC Bioinformatics 9:456.

Supplementary Table ST3. Dating constraints introduced for calibration. All ages are denoted in million years. Delinating taxa are given for most recent common ancestor (MRCA) identification of the dated node.

**Constraint**	**Delineating nodes (MRCA)**	**Minimum age**	**Maximum age**	**Source**
Fungi-Animalia split	*Caenorhabditis elegans* - *Aspergillus niger* ATCC 1015	875	1061	(Gueidan et al. 2011)
Basidiomycetes-Ascomycetes split	*Mixia osmundae* IAM 14324 – *Aspergillus niger* ATCC 1015	506	662	(Gueidan et al. 2011)
Taphrinomycotina/ Ascomycotina split	*Schizosaccharomyces pombe* 972h – *Aspergillus niger* ATCC 1015	462	612	(Gueidan et al. 2011)
Orbiliomycetes-Leotiomyceta split	*Arthrobotrys oligospora* ATCC 24927 – *Aspergillus niger* ATCC 1015	396	516	(Gueidan et al. 2011)
Pezizomycetes-Leotiomyceta split	*Tuber melanosporum* Mel28 – *Aspergillus niger* ATCC 1015	386	498	(Gueidan et al. 2011)
Hypocreales clade origin	*Nectria haematococca* MPVI – *Beauveria bassiana* ARSEF 2860	150	213	(Sung et al. 2008)
Hypocreaceae-Clavicipitaceae split	*Trichoderma atroviride* IMI 206040 – *Beauveria bassiana* ARSEF 2860	146	206	(Sung et al. 2008)
Fusarium solani clade origin	*Nectria haematococca* MPVI – *Fusarium oxysporum* f. sp. *lycopersici* 4287	32	108	(O'Donnell et al. 2013)
Dothideomycetes clade origin	*Baudoinia compniacensis* UAMH 10762 – *Cochliobolus heterostrophus* ATCC 48331	248	354	(Gueidan et al. 2011)
Mycospherellaceae clade origin	*Dothistroma septosporum* NZE10 – *Zymoseptoria tritici* IPO323	87	117	(Ohm et al. 2012)
Pleosporaceae clade origin	*Pyrenophora teres f. teres* 0-1 – *Cochliobolus heterostrophus* ATCC 48331	17	23	(Ohm et al. 2012)
Eurotiomycetes clade origin	*Exophiala dermatitidis* NIH/UT8656 – *Aspergillus niger* ATCC 1015	293	391	(Gueidan et al. 2011)

References:

Gueidan C, Ruibal C, de Hoog GS, Schneider H. 2011. Rock-inhabiting fungi originated during periods of dry climate in the late Devonian and middle Triassic. Fungal Biol. 115:987–996.

O'Donnell K, Rooney AP, Proctor RH, Brown DW, McCormick SP, Ward TJ, Frandsen RJN, Lysøe E, Rehner SA, Aoki T, et al. 2013. Phylogenetic analyses of RPB1 and RPB2 support a middle Cretaceous origin for a clade comprising all agriculturally and medically important fusaria. Fungal Genet. Biol. FG B 52:20–31.

Ohm RA, Feau N, Henrissat B, Schoch CL, Horwitz BA, Barry KW, Condon BJ, Copeland AC, Dhillon B, Glaser F, et al. 2012. Diverse lifestyles and strategies of plant pathogenesis encoded in the genomes of eighteen Dothideomycetes fungi. PLoS Pathog. 8:e1003037.

Sung G-H, Poinar Jr. GO, Spatafora JW. 2008. The oldest fossil evidence of animal parasitism by fungi supports a Cretaceous diversification of fungal–arthropod symbioses. Mol. Phylogenet. Evol. 49:495–502.

Supplementary Table ST4. Reference non-reducing PKSs with known activity and/or (end) products.

Gene name	Synonym	Description	Source
FGSG_03964	*pks14*	involved in orsellinic acid and orcinol biosynthesis	(Jørgensen et al. 2014)
CC1G_05377	*pks1*	involved in orsellinic acid synthesis (evidence from heterologous expression in yeast)	(Ishiuchi et al. 2012)
PMAA_101600	*pks12*	involved in mitorubrinol/mitorubrinic acid biosynthesis	(Woo et al. 2012)
ANIA_06448	*pkbA*	involved in cichorine biosynthesis	(Sanchez, Entwistle, et al. 2012)
CHGG_10128	-	involved in 6-methylorsellinic acid synthesis (evidence from heterologous expression in yeast)	(Ishiuchi et al. 2012)
ATEG_10080	*trt4*	involved in terretonin biosynthesis	(Guo et al. 2012)
ANIA_08383	*ausA*	involved in austinol biosynthesis	(Lo et al. 2012)
Pc21g05070	*sorbB*	involved in sorbicillinoid biosynthesis	(al Fahad et al. 2014)
PMAA_101860	*pks3*	involved in biosynthesis of red pigment/monascorubrin/ankaflavin/ citrinin	(Woo et al. 2014)
PMAA_101550	*pks11*	involved in mitorubrinol/mitorubrinic acid biosynthesis	(Woo et al. 2012)
TSTA_117750	*tropA*	involved in tropolone biosynthesis	(Davison et al. 2012)
ANIA_07903	*dbaI*	involved in biosynthesis of DHMBA (dihydroxy-3-methyl-6-(2-oxopropyl)benzaldehyde) and violaceols	(Gerke et al. 2012)
CHGG_10027	-	involved in 6-methylorsellinic acid synthesis (evidence from heterologous expression in yeast)	(Ishiuchi et al. 2012)
ANIA_03230	*pkfA*	involved in biosynthesis of aspernidine	(Yaegashi et al. 2013)
ANIA_01034	*afoE*	involved in asperfuranone biosynthesis	(Chiang et al. 2009)
ANIA_07909	*orsA*	involved in biosynthesis of orsellinic acid, F-9975 and violaceol derivatives	(Sanchez et al. 2010)
FGSG_02395	*zea1, pks13*	involved in zearalenone biosynthesis	(Gaffoor and Trail 2006)
ANIA_07071	*pkgA*	involved in alternariol biosynthesis	(Ahuja et al. 2012)
AFUA_4G00210	*encA*	involved in endocrocin biosynthesis	(Lim et al. 2012)
ANIA_06000	*aptA*	involved in asperthecin biosynthesis	(Szewczyk et al. 2008)
NFIA_112240	*nscA*	involved in neosartoricin biosynthesis	(Chooi et al. 2013)
ANIA_00150	*mdpG*	involved in biosynthesis of monodictyphenone, emodin and xanthone derivatives	(Sanchez et al. 2011)
ATEG_08451	*ACAS, gedC*	involved in biosynthesis of geodin and atrochrysone	(Awakawa et al. 2009; Nielsen et al. 2013)
NCU03584	*THNS*	tetrahydroxynaphtalene synthase, involved in melanin biosynthesis (single NR-PKS in *Neurospora crassa* genome)	-
CHGG_00542	-	involved in biosynthesis of O-methyldiaporthin	(Ishiuchi et al. 2012)

		(evidence from heterologous expression in yeast), ortholog of THNS	
HMPREF1120_03173	*WdPKS1*, *THNS*	tetrahydroxynaphtalene synthase, involved in melanin biosynthesis	(Feng et al. 2001)
NechaG33672	*pksN*	involved in biosynthesis of red perithecial pigment	(Graziani et al. 2004)
ATEG_07500	*pksP*	putative *pksP* homolog involved in YWA1/perithecial pigment biosynthesis (similarity-based annotation)	(Sanchez, Somoza, et al. 2012)
AFUB_033290	*alb1, pksP*	involved in melanin biosynthesis through naphtopyrone route	(Langfelder et al. 1998)
An09g05730/ ANI_1_726084	*fwnA, albA*	involved in melanin biosynthesis through naphtopyrone route	(Jørgensen et al. 2011)
FVEG_03379	*bikS*	involved in bikaverin synthesis (evidence from orthology with *Fusarium fujikuroi*, observed chemotypes)	(Wiemann et al. 2009)
FOXG_04757	*bikS*	involved in bikaverin synthesis (evidence from orthology with *Fusarium fujikuroi,* observed chemotypes)	(Wiemann et al. 2009)
FGSG_02324	*aur1, pks12*	involved in aurofusarin biosynthesis	(Frandsen et al. 2006)
TRIREDRAFT_82208	*pks4*	involved in conidial pigment biosynthesis	(Atanasova et al. 2013: 4)
ANIA_07825	*stcA, pksST*	involved in sterigmatocystin biosynthesis	(Yu et al. 2004)
AFLA_139410	*aflC*	involved in aflatoxin biosynthesis	(Yu et al. 2004)
PODANSg8570	-	involved in sterigmatocystin biosynthesis	(Slot and Rokas 2011)
NechaG101778	*pgl1, fsr1*	unknown product	(Wiemann et al. 2009)
FGSG_09182	*pgl1*	involved in biosynthesis of violet perithecial pigment (by orthology with *F. fujikuroi*)	(Wiemann et al. 2009)
FVEG_03695	*pgl1, fsr1*	involved in biosynthesis of perithecial pigment B and fusarubin (by orthology with *F. fujikuroi*)	(Wiemann et al. 2009)

References:

Ahuja M, Chiang Y-M, Chang S-L, Praseuth MB, Entwistle R, Sanchez JF, Lo H-C, Yeh H-H, Oakley BR, Wang CCC. 2012. Illuminating the diversity of aromatic polyketide synthases in Aspergillus nidulans. J. Am. Chem. Soc. 134:8212–8221.

Atanasova L, Knox BP, Kubicek CP, Druzhinina IS, Baker SE. 2013. The polyketide synthase gene pks4 of Trichoderma reesei provides pigmentation and stress resistance. Eukaryot. Cell 12:1499–1508.

Awakawa T, Yokota K, Funa N, Doi F, Mori N, Watanabe H, Horinouchi S. 2009. Physically discrete beta-lactamase-type thioesterase catalyzes product release in atrochrysone synthesis by iterative type I polyketide synthase. Chem. Biol. 16:613–623.

Chiang Y-M, Szewczyk E, Davidson AD, Keller N, Oakley BR, Wang CCC. 2009. A gene cluster containing two fungal polyketide synthases encodes the biosynthetic pathway for a polyketide, asperfuranone, in Aspergillus nidulans. J. Am. Chem. Soc. 131:2965–2970.

Chooi Y-H, Fang J, Liu H, Filler SG, Wang P, Tang Y. 2013. Genome mining of a prenylated and immunosuppressive polyketide from pathogenic fungi. Org. Lett. 15:780–783.

Davison J, al Fahad A, Cai M, Song Z, Yehia SY, Lazarus CM, Bailey AM, Simpson TJ, Cox RJ. 2012. Genetic, molecular, and biochemical basis of fungal tropolone biosynthesis. Proc. Natl. Acad. Sci. U. S. A. 109:7642–7647.

Al Fahad A, Abood A, Fisch KM, Osipow A, Davison J, Avramović M, Butts CP, Piel J, Simpson TJ, Cox RJ. 2014. Oxidative dearomatisation: the key step of sorbicillinoid biosynthesis. Chem. Sci. 5:523–527.

Feng B, Wang X, Hauser M, Kaufmann S, Jentsch S, Haase G, Becker JM, Szaniszlo PJ. 2001. Molecular cloning and characterization of WdPKS1, a gene involved in dihydroxynaphthalene melanin biosynthesis and virulence in Wangiella (Exophiala) dermatitidis. Infect. Immun. 69:1781–1794.

Frandsen RJN, Nielsen NJ, Maolanon N, Sørensen JC, Olsson S, Nielsen J, Giese H. 2006. The biosynthetic pathway for aurofusarin in Fusarium graminearum reveals a close link between the naphthoquinones and naphthopyrones. Mol. Microbiol. 61:1069–1080.

Gaffoor I, Trail F. 2006. Characterization of two polyketide synthase genes involved in zearalenone biosynthesis in Gibberella zeae. Appl. Environ. Microbiol. 72:1793–1799.

Gerke J, Bayram O, Feussner K, Landesfeind M, Shelest E, Feussner I, Braus GH. 2012. Breaking the silence: protein stabilization uncovers silenced biosynthetic gene clusters in the fungus Aspergillus nidulans. Appl. Environ. Microbiol. 78:8234–8244.

Graziani S, Vasnier C, Daboussi M-J. 2004. Novel polyketide synthase from Nectria haematococca. Appl. Environ. Microbiol. 70:2984–2988.

Guo C-J, Knox BP, Chiang Y-M, Lo H-C, Sanchez JF, Lee K-H, Oakley BR, Bruno KS, Wang CCC. 2012. Molecular genetic characterization of a cluster in A. terreus for biosynthesis of the meroterpenoid terretonin. Org. Lett. 14:5684–5687.

Ishiuchi K, Nakazawa T, Ookuma T, Sugimoto S, Sato M, Tsunematsu Y, Ishikawa N, Noguchi H, Hotta K, Moriya H, et al. 2012. Establishing a new methodology for genome mining and biosynthesis of polyketides and peptides through yeast molecular genetics. Chembiochem Eur. J. Chem. Biol. 13:846–854.

Jørgensen SH, Frandsen RJN, Nielsen KF, Lysøe E, Sondergaard TE, Wimmer R, Giese H, Sørensen JL. 2014. Fusarium graminearum PKS14 is involved in orsellinic acid and orcinol synthesis. Fungal Genet. Biol. FG B 70:24–31.

Jørgensen TR, Park J, Arentshorst M, van Welzen AM, Lamers G, Vankuyk PA, Damveld RA, van den Hondel CAM, Nielsen KF, Frisvad JC, et al. 2011. The molecular and genetic basis of conidial pigmentation in Aspergillus niger. Fungal Genet. Biol. FG B 48:544–553.

Langfelder K, Jahn B, Gehringer H, Schmidt A, Wanner G, Brakhage AA. 1998. Identification of a polyketide synthase gene (pksP) of Aspergillus fumigatus involved in conidial pigment biosynthesis and virulence. Med. Microbiol. Immunol. (Berl.) 187:79–89.

Lim FY, Hou Y, Chen Y, Oh J-H, Lee I, Bugni TS, Keller NP. 2012. Genome-based cluster deletion reveals an endocrocin biosynthetic pathway in Aspergillus fumigatus. Appl. Environ. Microbiol. 78:4117–4125.

Lo H-C, Entwistle R, Guo C-J, Ahuja M, Szewczyk E, Hung J-H, Chiang Y-M, Oakley BR, Wang CCC. 2012. Two separate gene clusters encode the biosynthetic pathway for the

meroterpenoids austinol and dehydroaustinol in Aspergillus nidulans. J. Am. Chem. Soc. 134:4709–4720.

Nielsen MT, Nielsen JB, Anyaogu DC, Anyaogu DC, Holm DK, Nielsen KF, Larsen TO, Mortensen UH. 2013. Heterologous reconstitution of the intact geodin gene cluster in Aspergillus nidulans through a simple and versatile PCR based approach. PloS One 8:e72871.

Sanchez JF, Chiang Y-M, Szewczyk E, Davidson AD, Ahuja M, Elizabeth Oakley C, Woo Bok J, Keller N, Oakley BR, Wang CCC. 2010. Molecular genetic analysis of the orsellinic acid/F9775 gene cluster of Aspergillus nidulans. Mol. Biosyst. 6:587–593.

Sanchez JF, Entwistle R, Corcoran D, Oakley BR, Wang CCC. 2012. Identification and molecular genetic analysis of the cichorine gene cluster in Aspergillus nidulans. MedChemComm 3.

Sanchez JF, Entwistle R, Hung J-H, Yaegashi J, Jain S, Chiang Y-M, Wang CCC, Oakley BR. 2011. Genome-based deletion analysis reveals the prenyl xanthone biosynthesis pathway in Aspergillus nidulans. J. Am. Chem. Soc. 133:4010–4017.

Sanchez JF, Somoza AD, Keller NP, Wang CCC. 2012. Advances in Aspergillus secondary metabolite research in the post-genomic era. Nat. Prod. Rep. 29:351–371.

Slot JC, Rokas A. 2011. Horizontal transfer of a large and highly toxic secondary metabolic gene cluster between fungi. Curr. Biol. CB 21:134–139.

Szewczyk E, Chiang Y-M, Oakley CE, Davidson AD, Wang CCC, Oakley BR. 2008. Identification and characterization of the asperthecin gene cluster of Aspergillus nidulans. Appl. Environ. Microbiol. 74:7607–7612.

Wiemann P, Willmann A, Straeten M, Kleigrewe K, Beyer M, Humpf H-U, Tudzynski B. 2009. Biosynthesis of the red pigment bikaverin in Fusarium fujikuroi: genes, their function and regulation. Mol. Microbiol. 72:931–946.

Woo PCY, Lam C-W, Tam EWT, Lee K-C, Yung KKY, Leung CKF, Sze K-H, Lau SKP, Yuen K-Y. 2014. The biosynthetic pathway for a thousand-year-old natural food colorant and citrinin in Penicillium marneffei. Sci. Rep. 4:6728.

Woo PCY, Lam C-W, Tam EWT, Leung CKF, Wong SSY, Lau SKP, Yuen K-Y. 2012. First discovery of two polyketide synthase genes for mitorubrinic acid and mitorubrinol yellow pigment biosynthesis and implications in virulence of Penicillium marneffei. PLoS Negl. Trop. Dis. 6:e1871.

Yaegashi J, Praseuth MB, Tyan S-W, Sanchez JF, Entwistle R, Chiang Y-M, Oakley BR, Wang CCC. 2013. Molecular genetic characterization of the biosynthesis cluster of a prenylated isoindolinone alkaloid aspernidine A in Aspergillus nidulans. Org. Lett. 15:2862–2865.

Yu J, Chang P-K, Ehrlich KC, Cary JW, Bhatnagar D, Cleveland TE, Payne GA, Linz JE, Woloshuk CP, Bennett JW. 2004. Clustered pathway genes in aflatoxin biosynthesis. Appl. Environ. Microbiol. 70:1253–1262.

Supplementary Table ST5. Event counts and optimal solution costs across different parameter values for DTL-RANGER analysis of the original (Bayesian Inference consensus) and amalgamated (ALE) tree topologies. The shown parameter combinations are limited to transfer costs between 5 and 20. DTL-RANGER solutions could not be obtained for the cost combination of <D=4, T=5, L=3> (indicated by N/A values).

Costs			Event counts (average; 1000 random optima)								Optimal reconciliation cost	
D	T	L	Duplications		Transfers		Losses		Σ (all)			
			original	ALE	original	ALE	original	ALE	original	ALE	original	ALE
**4**	**5**	**1**	11.63	17.79	189.03	153.74	149.32	217.13	349.98	388.66	1141	1057
**4**	**6**	**1**	20.65	25.66	168.24	135.65	224.99	285.49	413.88	446.79	1317	1202
**4**	**7**	**1**	45.47	47.19	133.31	87.90	353.97	511.90	532.75	647.00	1469	1316
**4**	**8**	**1**	73.05	62.56	81.60	61.62	630.03	650.79	784.68	774.97	1575	1394
**4**	**9**	**1**	84.67	77.01	66.33	40.99	712.34	764.04	863.34	882.04	1648	1441
**4**	**10**	**1**	92.98	85.06	56.55	28.42	774.59	848.53	924.12	962.01	1712	1473
**4**	**11**	**1**	103.29	88.00	43.71	25.00	864.02	871.00	1011.02	984.00	1758	1498
**4**	**12**	**1**	119.07	88.50	24.93	24.50	1016.58	875.00	1160.58	988.00	1792	1523
**4**	**13**	**1**	126.84	96.27	17.47	15.73	1077.52	954.43	1221.83	1066.43	1812	1544
**4**	**14**	**1**	131.53	97.47	13.98	14.53	1105.18	965.69	1250.69	1077.69	1827	1559
**4**	**15**	**1**	133.80	99.80	12.60	13.10	1115.81	977.29	1262.21	1090.19	1840	1573
**4**	**16**	**1**	140.50	104.51	8.51	10.49	1151.94	998.12	1300.94	1113.12	1850	1584
**4**	**17**	**1**	144.00	106.62	6.00	9.04	1179.00	1013.91	1329.00	1129.57	1857	1594
**4**	**18**	**1**	144.00	110.51	6.00	6.49	1179.00	1042.11	1329.00	1159.11	1863	1601
**4**	**19**	**1**	144.96	111.00	5.52	6.00	1184.29	1049.00	1334.77	1166.00	1869	1607
**4**	**20**	**1**	146.00	111.00	5.00	6.00	1190.00	1049.00	1341.00	1166.00	1874	1613
**4**	**5**	**2**	8.00	6.40	218.00	204.14	46.00	54.35	272.00	264.89	1214	1155
**4**	**6**	**2**	8.57	7.02	217.01	194.55	47.83	78.31	273.41	279.88	1432	1352
**4**	**7**	**2**	11.54	12.00	212.46	187.00	56.32	93.00	280.32	292.00	1646	1543
**4**	**8**	**2**	20.92	19.86	180.31	164.37	161.93	164.79	363.16	349.02	1850	1724
**4**	**9**	**2**	29.01	26.00	171.67	145.00	181.48	233.00	382.15	404.00	2024	1875
**4**	**10**	**2**	35.09	32.99	163.60	136.12	207.82	263.42	406.51	432.53	2192	2020
**4**	**11**	**2**	47.24	44.668	138.98	112.664	312.14	361.012	498.35	518.34	2342	2140
**4**	**12**	**2**	56.86	50.368	123.37	102.988	383.05	405.336	563.28	558.69	2474	2248

**4**	**13**	**2**	67.03	62.550	103.94	74.994	483.32	555.439	654.29	692.98	2586	2336
**4**	**14**	**2**	79.91	69.212	79.65	58.666	622.61	650.914	782.17	778.79	2680	2400
**4**	**15**	**2**	86.55	78.000	68.89	40.000	686.20	769.000	841.65	887.00	2752	2450
**4**	**16**	**2**	91.19	79.317	61.99	38.539	730.69	778.054	883.87	895.91	2818	2490
**4**	**17**	**2**	95.64	86.000	56.36	28.000	767.16	849.000	919.16	963.00	2875	2518
**4**	**18**	**2**	104.23	88.000	45.85	25.000	842.85	871.000	992.94	984.00	2928	2544
**4**	**19**	**2**	109.00	88.000	40.00	25.000	886.00	871.000	1035.00	984.00	2968	2569
**4**	**20**	**2**	122.11	88.516	24.42	24.484	1013.61	875.128	1160.14	988.13	3004	2594
**4**	**5**	**3**	8.00	N/A	223.00	N/A	36.00	N/A	267.00	0.00	1255	N/A
**4**	**6**	**3**	8.00	7.000	221.79	212.542	38.42	35.916	268.21	255.46	1478	1411
**4**	**7**	**3**	8.58	10.976	217.42	202.914	46.58	50.566	272.58	264.46	1696	1616
**4**	**8**	**3**	10.68	19.377	215.66	193.377	48.00	62.492	274.34	275.25	1912	1812
**4**	**9**	**3**	14.00	19.000	211.96	192.199	54.14	66.403	280.09	277.60	2126	2005
**4**	**10**	**3**	14.67	18.810	210.33	184.772	58.35	90.680	283.35	294.26	2337	2195
**4**	**11**	**3**	29.00	21.348	175.00	177.304	168.00	113.088	372.00	311.74	2545	2375
**4**	**12**	**3**	31.00	25.000	171.85	160.180	177.62	172.280	380.46	357.46	2719	2539
**4**	**13**	**3**	34.22	28.977	166.78	143.023	194.67	236.931	395.67	408.93	2889	2686
**4**	**14**	**3**	39.00	35.550	160.00	133.900	218.00	269.400	417.00	438.85	3050	2825
**4**	**15**	**3**	42.00	43.733	152.00	116.784	253.00	341.436	447.00	501.95	3207	2951
**4**	**16**	**3**	53.18	46.802	132.82	109.646	335.70	374.152	521.70	530.60	3345	3064
**4**	**17**	**3**	59.85	53.272	121.29	101.364	389.91	409.908	571.04	564.54	3471	3166
**4**	**18**	**3**	63.82	60.467	113.28	81.555	431.23	517.714	608.33	659.74	3588	3263
**4**	**19**	**3**	70.16	70.000	99.74	66.000	505.46	600.000	675.35	736.00	3692	3334
**4**	**20**	**3**	84.00	71.018	78.00	61.964	627.00	625.216	789.00	758.20	3777	3399

Supplementary Table ST6. Fraction of event labels (speciation, transfer donor, duplication) shared between DTL-RANGER and ALE for both original and amalgamated (ALE) tree topology. For DTL-RANGER type of event was determined by frequency within sampled reconciliations. Solutions could not be obtained for the cost combination of <D=4, T=5, L=3> (indicated by N/A values). The shown parameter combinations are limited to transfer costs between 5 and 20. For the original tree (Bayesian consensus) only the 352 non-rearranged bipartitions (shared between amalgamated and original trees) were taken into account.

Costs			Frequency of identical predictions between DTL-RANGER and ALE							
D	T	L	Duplication		Speciation		Transfer		Σ (all)	
			original	ALE	original	ALE	original	ALE	original	ALE
**4**	**5**	**1**	1.000	1.000	0.842	0.937	0.173	0.160	0.582	0.792
**4**	**6**	**1**	1.000	1.000	0.833	0.941	0.198	0.182	0.622	0.817
**4**	**7**	**1**	0.806	1.000	0.851	0.958	0.238	0.284	0.670	0.888
**4**	**8**	**1**	0.803	0.969	0.897	0.971	0.414	0.400	0.801	0.930
**4**	**9**	**1**	0.817	0.974	0.932	0.987	0.489	0.600	0.849	0.967
**4**	**10**	**1**	0.805	0.976	0.940	0.993	0.575	0.857	0.869	0.987
**4**	**11**	**1**	0.767	0.977	0.940	0.997	0.645	0.960	0.872	0.994
**4**	**12**	**1**	0.760	0.977	0.945	0.997	0.789	0.960	0.886	0.994
**4**	**13**	**1**	0.730	0.897	0.928	0.987	0.733	1.000	0.864	0.977
**4**	**14**	**1**	0.724	0.897	0.932	0.987	0.900	1.000	0.869	0.977
**4**	**15**	**1**	0.724	0.870	0.932	0.985	0.900	1.000	0.869	0.971
**4**	**16**	**1**	0.694	0.829	0.932	0.983	1.000	1.000	0.858	0.964
**4**	**17**	**1**	0.675	0.829	0.919	0.983	1.000	1.000	0.841	0.964
**4**	**18**	**1**	0.675	0.784	0.919	0.977	1.000	1.000	0.841	0.952
**4**	**19**	**1**	0.675	0.784	0.919	0.977	1.000	1.000	0.841	0.952
**4**	**20**	**1**	0.664	0.784	0.927	0.977	1.000	1.000	0.841	0.952
**4**	**5**	**2**	0.833	1.000	0.868	0.953	0.146	0.123	0.531	0.748
**4**	**6**	**2**	0.857	1.000	0.878	0.947	0.146	0.128	0.537	0.754
**4**	**7**	**2**	0.900	1.000	0.884	0.949	0.149	0.134	0.548	0.765
**4**	**8**	**2**	0.938	1.000	0.847	0.945	0.179	0.151	0.597	0.787
**4**	**9**	**2**	0.846	1.000	0.855	0.946	0.190	0.172	0.616	0.812
**4**	**10**	**2**	0.774	1.000	0.870	0.950	0.198	0.184	0.631	0.825

**4**	**11**	**2**	0.732	1.000	0.863	0.957	0.226	0.223	0.656	0.859
**4**	**12**	**2**	0.739	1.000	0.867	0.960	0.250	0.238	0.682	0.870
**4**	**13**	**2**	0.772	0.969	0.891	0.977	0.320	0.333	0.750	0.920
**4**	**14**	**2**	0.812	0.971	0.934	0.980	0.429	0.414	0.830	0.939
**4**	**15**	**2**	0.803	0.974	0.926	0.987	0.460	0.600	0.835	0.967
**4**	**16**	**2**	0.795	0.974	0.931	0.987	0.489	0.600	0.844	0.967
**4**	**17**	**2**	0.782	0.977	0.936	0.996	0.537	0.857	0.855	0.989
**4**	**18**	**2**	0.770	0.977	0.949	0.997	0.677	0.960	0.881	0.994
**4**	**19**	**2**	0.756	0.977	0.948	0.997	0.690	0.960	0.878	0.994
**4**	**20**	**2**	0.765	0.977	0.958	0.997	0.889	0.960	0.901	0.994
**4**	**5**	**3**	0.833	N/A	0.875	N/A	0.141	N/A	0.520	N/A
**4**	**6**	**3**	0.833	1.000	0.875	0.957	0.141	0.117	0.520	0.739
**4**	**7**	**3**	0.833	1.000	0.873	0.962	0.145	0.123	0.531	0.756
**4**	**8**	**3**	0.875	0.947	0.878	0.962	0.147	0.130	0.540	0.767
**4**	**9**	**3**	0.917	0.947	0.889	0.962	0.150	0.130	0.554	0.767
**4**	**10**	**3**	0.917	1.000	0.889	0.955	0.150	0.137	0.554	0.775
**4**	**11**	**3**	0.846	0.952	0.858	0.957	0.186	0.140	0.611	0.781
**4**	**12**	**3**	0.786	1.000	0.852	0.951	0.188	0.155	0.605	0.798
**4**	**13**	**3**	0.767	1.000	0.859	0.948	0.194	0.174	0.616	0.815
**4**	**14**	**3**	0.765	1.000	0.874	0.951	0.202	0.185	0.636	0.828
**4**	**15**	**3**	0.722	1.000	0.867	0.955	0.212	0.214	0.642	0.852
**4**	**16**	**3**	0.732	1.000	0.870	0.957	0.231	0.223	0.665	0.859
**4**	**17**	**3**	0.755	1.000	0.876	0.964	0.258	0.245	0.696	0.878
**4**	**18**	**3**	0.750	0.983	0.873	0.969	0.273	0.298	0.705	0.902
**4**	**19**	**3**	0.783	0.957	0.900	0.978	0.333	0.364	0.764	0.927
**4**	**20**	**3**	0.792	0.958	0.934	0.981	0.426	0.400	0.827	0.937

Supplementary Table ST7. Fraction of event mappings (species tree nodes) shared between DTL-RANGER and ALE for both original and amalgamated (ALE) trees. Measure is tentative as ALE and DTL-RANGER model transfer process differently (with ALE explicitly modelling two-step transfer through extinct intermediate lineages). Mapping was taken to be in accord, if (all of) the most frequent predictions by DTL-RANGER were indicated in the ALE output for given node. Solutions could not be obtained for the cost combination of <D=4, T=5, L=3> (indicated by N/A values). The shown parameter combinations are limited to transfer costs between 5 and 20. For the original tree (Bayesian consensus) only the 352 non-rearranged bipartitions (shared between amalgamated and original trees) were taken into account.

**Costs**			**Frequency of identical predictions between DTL-RANGER and ALE**							
**D**	**T**	**L**	**Duplication**		**Speciation**		**Transfer**		**Σ (all)**	
			**original**	**ALE**	**original**	**ALE**	**original**	**ALE**	**original**	**ALE**
**4**	**5**	**1**	0.700	0.824	0.828	0.948	0.086	0.122	0.531	0.789
**4**	**6**	**1**	0.533	0.792	0.819	0.950	0.099	0.153	0.560	0.813
**4**	**7**	**1**	0.778	0.872	0.851	0.967	0.158	0.227	0.645	0.882
**4**	**8**	**1**	0.869	0.938	0.906	0.977	0.310	0.400	0.801	0.932
**4**	**9**	**1**	0.887	0.949	0.919	0.987	0.383	0.500	0.841	0.960
**4**	**10**	**1**	0.896	0.976	0.932	0.993	0.525	0.821	0.878	0.985
**4**	**11**	**1**	0.919	0.989	0.936	0.996	0.548	0.880	0.898	0.992
**4**	**12**	**1**	0.938	0.989	0.937	0.996	0.737	0.920	0.926	0.993
**4**	**13**	**1**	0.930	0.938	0.920	0.983	0.667	0.867	0.912	0.976
**4**	**14**	**1**	0.933	0.938	0.920	0.983	0.700	0.867	0.918	0.976
**4**	**15**	**1**	0.933	0.930	0.920	0.980	0.700	0.846	0.918	0.972
**4**	**16**	**1**	0.892	0.886	0.923	0.982	1.000	1.000	0.915	0.970
**4**	**17**	**1**	0.860	0.886	0.911	0.982	1.000	0.900	0.895	0.968
**4**	**18**	**1**	0.860	0.838	0.911	0.976	1.000	1.000	0.895	0.958
**4**	**19**	**1**	0.860	0.838	0.911	0.976	1.000	1.000	0.895	0.958
**4**	**20**	**1**	0.845	0.838	0.918	0.976	1.000	1.000	0.895	0.958
**4**	**5**	**2**	0.667	0.667	0.830	0.961	0.079	0.083	0.477	0.742
**4**	**6**	**2**	0.714	0.857	0.834	0.952	0.073	0.092	0.477	0.748
**4**	**7**	**2**	0.500	0.583	0.834	0.950	0.075	0.080	0.477	0.748
**4**	**8**	**2**	0.563	0.611	0.827	0.952	0.090	0.114	0.534	0.776

**4**	**9**	**2**	0.500	0.846	0.835	0.950	0.087	0.124	0.543	0.801
**4**	**10**	**2**	0.516	0.758	0.860	0.953	0.124	0.140	0.577	0.811
**4**	**11**	**2**	0.537	0.711	0.878	0.963	0.132	0.179	0.614	0.842
**4**	**12**	**2**	0.717	0.735	0.876	0.966	0.188	0.200	0.668	0.855
**4**	**13**	**2**	0.754	0.862	0.886	0.977	0.227	0.278	0.724	0.907
**4**	**14**	**2**	0.855	0.928	0.921	0.980	0.286	0.345	0.807	0.931
**4**	**15**	**2**	0.901	0.949	0.918	0.987	0.360	0.500	0.835	0.960
**4**	**16**	**2**	0.890	0.949	0.927	0.987	0.383	0.500	0.847	0.960
**4**	**17**	**2**	0.897	0.988	0.927	0.994	0.390	0.821	0.858	0.988
**4**	**18**	**2**	0.897	0.989	0.940	0.996	0.516	0.920	0.892	0.993
**4**	**19**	**2**	0.911	0.989	0.944	0.996	0.552	0.920	0.903	0.993
**4**	**20**	**2**	0.939	0.989	0.945	0.996	0.889	0.920	0.940	0.993
**4**	**5**	**3**	0.667	N/A	0.835	N/A	0.071	N/A	0.463	N/A
**4**	**6**	**3**	0.667	0.571	0.841	0.960	0.065	0.070	0.463	0.726
**4**	**7**	**3**	0.667	0.417	0.834	0.967	0.079	0.074	0.477	0.739
**4**	**8**	**3**	0.625	0.368	0.829	0.967	0.074	0.078	0.474	0.745
**4**	**9**	**3**	0.417	0.368	0.833	0.966	0.069	0.062	0.472	0.741
**4**	**10**	**3**	0.417	0.588	0.844	0.957	0.081	0.093	0.483	0.758
**4**	**11**	**3**	0.500	0.524	0.832	0.957	0.078	0.084	0.531	0.758
**4**	**12**	**3**	0.536	0.520	0.832	0.955	0.102	0.112	0.543	0.777
**4**	**13**	**3**	0.567	0.786	0.838	0.951	0.105	0.125	0.557	0.801
**4**	**14**	**3**	0.529	0.714	0.859	0.954	0.126	0.133	0.580	0.810
**4**	**15**	**3**	0.500	0.636	0.867	0.959	0.142	0.162	0.597	0.829
**4**	**16**	**3**	0.561	0.711	0.884	0.963	0.144	0.188	0.628	0.844
**4**	**17**	**3**	0.673	0.750	0.881	0.969	0.161	0.206	0.662	0.861
**4**	**18**	**3**	0.673	0.831	0.873	0.972	0.193	0.274	0.673	0.891
**4**	**19**	**3**	0.767	0.871	0.891	0.978	0.236	0.318	0.736	0.916
**4**	**20**	**3**	0.861	0.889	0.925	0.981	0.333	0.367	0.821	0.928

Supplementary Table ST8. Splice site locations significantly associated with monophyletic clades (P<0.0001). Locations mapped to consensus sequences of core NR-PKS domains (SAT – starter acyl transferase, KS – ketoacyl synthase, AT – acyl transferase, PT – product template, TE – thioesterase). Inherited splice junctions (i.e. overrepresented at an ancestor node) are given in parentheses.

**Clade(s)**	**Gene tree node**	**SAT**	**KS**	**AT**	**PT**	**TE**
α	*g66*	403	36, 219, 283	262	-	299
β	*g182*	(455)	258	48	-	-
	g341	455	-	-	-	-
**γ.C2-C7a**	*g404*	(174),(404)	(515), 622	-	-	-
	g405	(174),(404)	515	-	-	-
**γ.C6-C11**	*g454*	(174),(404)	(122), 278	-	(67)	-
	g494	(174),(404)	122	-	(67)	-
	g550	(174),(404)	254	-	(67)	-
**γ.C2-C7b**	*g626*	(174),(404)	-	-	(67)	597
**γ.C2-C7c**	*g628*	(174),(404)	230, (334)	-	(67), (616)	-
	g725	(174),(404)	334	-	(67), (616)	-
**γ.C4-C9**	*g809*	(174), 203, (404)	-	-	(67), (247), (616)	(592)
	g813	(174),(404)	-	-	(67), (247), (616)	592
	g814	(174),(404)	-	-	(67), 247, (616)	-
**γ.C2-C7c+γ.C4-C9**	*g815*	(174),(404)	-	-	(67), 616	-
**γ.C2-C7b/c+γ.C4-C9+γ.C6-C11**	*g820*	(174),(404)	-	-	67	-
**γ.C2-C7a/b/c+γ.C4-C9+γ.C6-C11**	*g821*	174,404	-	-	-	-

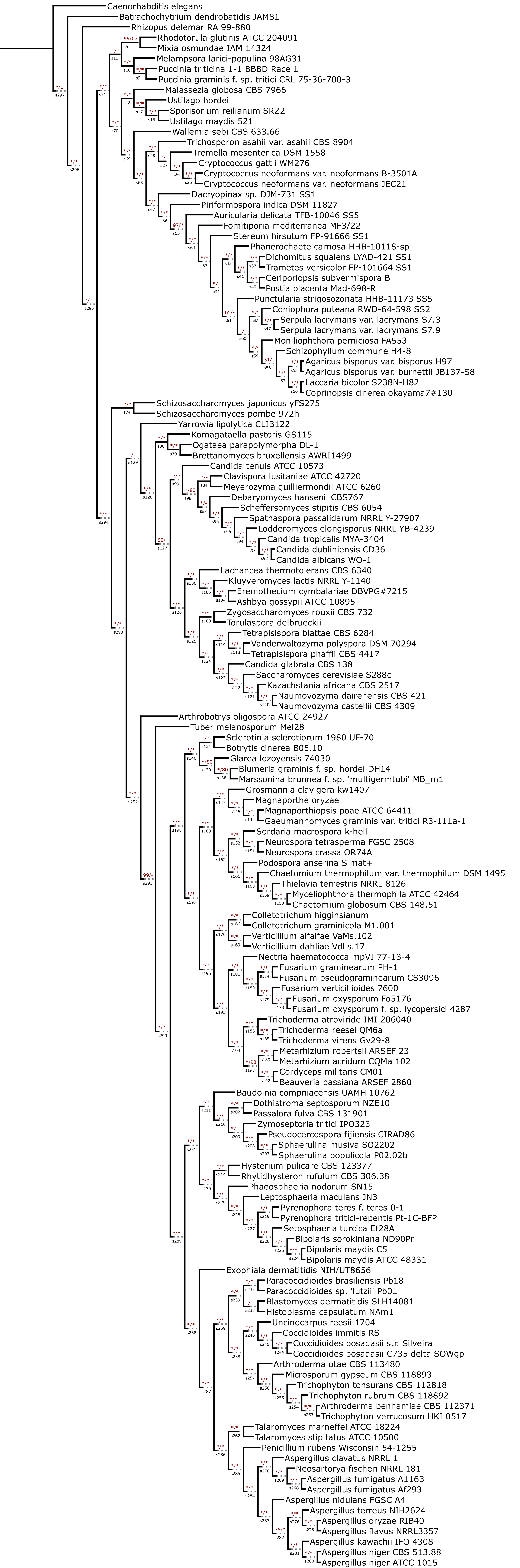
Caenorhabditis elegans
Batrachochytrium dendrobatidis JAM81
Rhizopus delemar RA 99-880
Rhodotorula glutinis ATCC 204091
Mixia osmundae IAM 14324
Melampsora larici-populina 98AG31
Puccinia triticina 1-1 BBBD Race 1
Puccinia graminis f. sp. tritici CRL 75-36-700-3
Malassezia globosa CBS 7966
Ustilago hordei
Sporisorium reilianum SRZ2
Ustilago maydis 521
Wallemia sebi CBS 633.66
Trichosporon asahii var. asahii CBS 8904
Tremella mesenterica DSM 1558
Cryptococcus gattii WM276
Cryptococcus neoformans var. neoformans B-3501A
Cryptococcus neoformans var. neoformans JEC21
Dacryopinax sp. DJM-731 SS1
Piriformospora indica DSM 11827
Auricularia delicata TFB-10046 SS5
Fomitiporia mediterranea MF3/22
Stereum hirsutum FP-91666 SS1
Phanerochaete carnosa HHB-10118-sp
Dichomitus squalens LYAD-421 SS1
Trametes versicolor FP-101664 SS1
Ceriporiopsis subvermispora B
Postia placenta Mad-698-R
Punctularia strigosozonata HHB-11173 SS5
Coniophora puteana RWD-64-598 SS2
Serpula lacrymans var. lacrymans S7.3
Serpula lacrymans var. lacrymans S7.9
Moniliophthora perniciosa FA553
Schizophyllum commune H4-8
Agaricus bisporus var. bisporus H97
Agaricus bisporus var. burnettii JB137-S8
Laccaria bicolor S238N-H82
Coprinopsis cinerea okayama7#130
Schizosaccharomyces japonicus yFS275
Schizosaccharomyces pombe 972h-
Yarrowia lipolytica CLIB122
Komagataella pastoris GS115
Ogataea parapolymorpha DL-1
Brettanomyces bruxellensis AWRI1499
Candida tenuis ATCC 10573
Clavispora lusitaniae ATCC 42720
Meyerozyma guilliermondii ATCC 6260
Debaryomyces hansenii CBS767
Scheffersomyces stipitis CBS 6054
Spathaspora passalidarum NRRL Y-27907
Lodderomyces elongisporus NRRL YB-4239
Candida tropicalis MYA-3404
Candida dubliniensis CD36
Candida albicans WO-1
Lachancea thermotolerans CBS 6340
Kluyveromyces lactis NRRL Y-1140
Eremothecium cymbalariae DBVPG#7215
Ashbya gossypii ATCC 10895
Zygosaccharomyces rouxii CBS 732
Torulaspora delbrueckii
Tetrapisispora blattae CBS 6284
Vanderwaltozyma polyspora DSM 70294
Tetrapisispora phaffii CBS 4417
Candida glabrata CBS 138
Saccharomyces cerevisiae S288c
Kazachstania africana CBS 2517
Naumovozyma dairenensis CBS 421
Naumovozyma castellii CBS 4309
Arthrobotrys oligospora ATCC 24927
Tuber melanosporum Mel28
Sclerotinia sclerotiorum 1980 UF-70
Botrytis cinerea B05.10
Glarea lozoyensis 74030
Blumeria graminis f. sp. hordei DH14
Marssonina brunnea f. sp. 'multigermtubi' MB_m1
Grosmannia clavigera kw1407
Magnaporthe oryzae
Magnaporthiopsis poae ATCC 64411
Gaeumannomyces graminis var. tritici R3-111a-1
Sordaria macrospora k-hell
Neurospora tetrasperma FGSC 2508
Neurospora crassa OR74A
Podospora anserina S mat+
Chaetomium thermophilum var. thermophilum DSM 1495
Thielavia terrestris NRRL 8126
Myceliophthora thermophila ATCC 42464
Chaetomium globosum CBS 148.51
Colletotrichum higginsianum
Colletotrichum graminicola M1.001
Verticillium alfalfae VaMs.102
Verticillium dahliae VdLs.17
Nectria haematococca mpVI 77-13-4
Fusarium graminearum PH-1
Fusarium pseudograminearum CS3096
Fusarium verticillioides 7600
Fusarium oxysporum Fo5176
Fusarium oxysporum f. sp. lycopersici 4287
Trichoderma atroviride IMI 206040
Trichoderma reesei QM6a
Trichoderma virens Gv29-8
Metarhizium robertsii ARSEF 23
Metarhizium acridum CQMa 102
Cordyceps militaris CM01
Beauveria bassiana ARSEF 2860
Baudoinia compniacensis UAMH 10762
Dothistroma septosporum NZE10
Passalora fulva CBS 131901
Zymoseptoria tritici IPO323
Pseudocercospora fijiensis CIRAD86
Sphaerulina musiva SO2202
Sphaerulina populicola P02.02b
Hysterium pulicare CBS 123377
Rhytidhysteron rufulum CBS 306.38
Phaeosphaeria nodorum SN15
Leptosphaeria maculans JN3
Pyrenophora teres f. teres 0-1
Pyrenophora tritici-repentis Pt-1C-BFP
Setosphaeria turcica Et28A
Bipolaris sorokiniana ND90Pr
Bipolaris maydis C5
Bipolaris maydis ATCC 48331
Exophiala dermatitidis NIH/UT8656
Paracoccidioides brasiliensis Pb18
Paracoccidioides sp. 'lutzii' Pb01
Blastomyces dermatitidis SLH14081
Histoplasma capsulatum NAm1
Uncinocarpus reesii 1704
Coccidioides immitis RS
Coccidioides posadasii str. Silveira
Coccidioides posadasii C735 delta SOWgp
Arthroderma otae CBS 113480
Microsporum gypseum CBS 118893
Trichophyton tonsurans CBS 112818
Trichophyton rubrum CBS 118892
Arthroderma benhamiae CBS 112371
Trichophyton verrucosum HKI 0517
Talaromyces marneffei ATCC 18224
Talaromyces stipitatus ATCC 10500
Penicillium rubens Wisconsin 54-1255
Aspergillus clavatus NRRL 1
Neosartorya fischeri NRRL 181
Aspergillus fumigatus A1163
Aspergillus fumigatus Af293
Aspergillus nidulans FGSC A4
Aspergillus terreus NIH2624
Aspergillus oryzae RIB40
Aspergillus flavus NRRL3357
Aspergillus kawachii IFO 4308
Aspergillus niger CBS 513.88
Aspergillus niger ATCC 1015

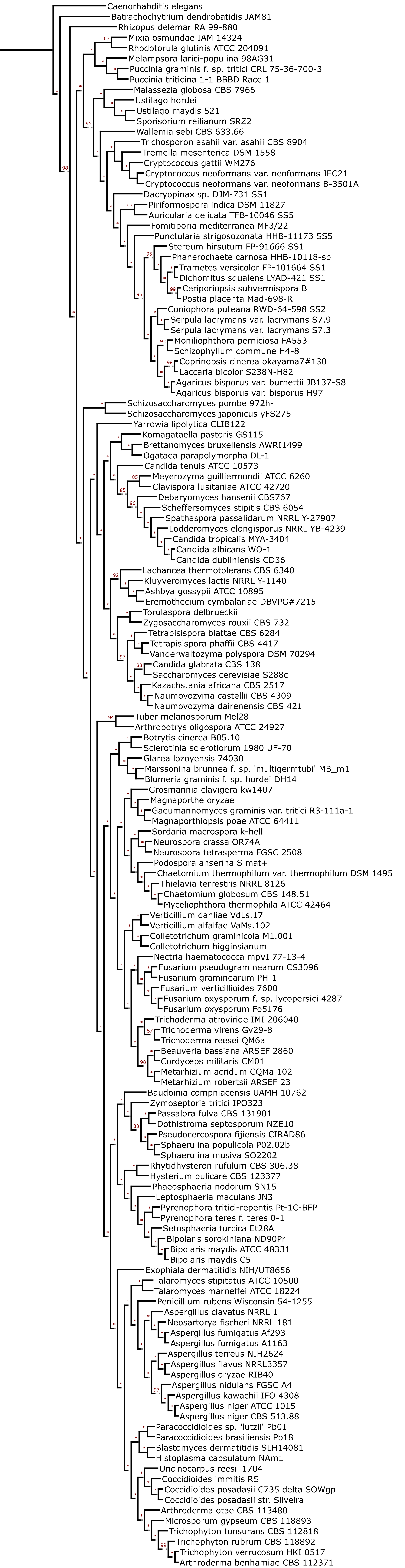

Caenorhabditis elegans
Batrachochytrium dendrobatidis JAM81
Rhizopus delemar RA 99-880
Mixia osmundae IAM 14324
Rhodotorula glutinis ATCC 204091
Melampsora larici-populina 98AG31
Puccinia graminis f. sp. tritici CRL 75-36-700-3
Puccinia triticina 1-1 BBBD Race 1
Malassezia globosa CBS 7966
Ustilago hordei
Ustilago maydis 521
Sporisorium reilianum SRZ2
Wallemia sebi CBS 633.66
Trichosporon asahii var. asahii CBS 8904
Tremella mesenterica DSM 1558
Cryptococcus gattii WM276
Cryptococcus neoformans var. neoformans JEC21
Cryptococcus neoformans var. neoformans B-3501A
Dacryopinax sp. DJM-731 SS1
Piriformospora indica DSM 11827
Auricularia delicata TFB-10046 SS5
Fomitiporia mediterranea MF3/22
Punctularia strigosozonata HHB-11173 SS5
Stereum hirsutum FP-91666 SS1
Phanerochaete carnosa HHB-10118-sp
Trametes versicolor FP-101664 SS1
Dichomitus squalens LYAD-421 SS1
Ceriporiopsis subvermispora B
Postia placenta Mad-698-R
Coniophora puteana RWD-64-598 SS2
Serpula lacrymans var. lacrymans S7.9
Serpula lacrymans var. lacrymans S7.3
Moniliophthora perniciosa FA553
Schizophyllum commune H4-8
Coprinopsis cinerea okayama7#130
Laccaria bicolor S238N-H82
Agaricus bisporus var. burnettii JB137-S8
Agaricus bisporus var. bisporus H97
Schizosaccharomyces pombe 972h-
Schizosaccharomyces japonicus yFS275
Yarrowia lipolytica CLIB122
Komagataella pastoris GS115
Brettanomyces bruxellensis AWRI1499
Ogataea parapolymorpha DL-1
Candida tenuis ATCC 10573
Meyerozyma guilliermondii ATCC 6260
Clavispora lusitaniae ATCC 42720
Debaryomyces hansenii CBS767
Scheffersomyces stipitis CBS 6054
Spathaspora passalidarum NRRL Y-27907
Lodderomyces elongisporus NRRL YB-4239
Candida tropicalis MYA-3404
Candida albicans WO-1
Candida dubliniensis CD36
Lachancea thermotolerans CBS 6340
Kluyveromyces lactis NRRL Y-1140
Ashbya gossypii ATCC 10895
Eremothecium cymbalariae DBVPG#7215
Torulaspora delbrueckii
Zygosaccharomyces rouxii CBS 732
Tetrapisispora blattae CBS 6284
Tetrapisispora phaffii CBS 4417
Vanderwaltozyma polyspora DSM 70294
Candida glabrata CBS 138
Saccharomyces cerevisiae S288c
Kazachstania africana CBS 2517
Naumovozyma castellii CBS 4309
Naumovozyma dairenensis CBS 421
Tuber melanosporum Mel28
Arthrobotrys oligospora ATCC 24927
Botrytis cinerea B05.10
Sclerotinia sclerotiorum 1980 UF-70
Glarea lozoyensis 74030
Marssonina brunnea f. sp. 'multigermtubi' MB_m1
Blumeria graminis f. sp. hordei DH14
Grosmannia clavigera kw1407
Magnaporthe oryzae
Gaeumannomyces graminis var. tritici R3-111a-1
Magnaporthiopsis poae ATCC 64411
Sordaria macrospora k-hell
Neurospora crassa OR74A
Neurospora tetrasperma FGSC 2508
Podospora anserina S mat+
Chaetomium thermophilum var. thermophilum DSM 1495
Thielavia terrestris NRRL 8126
Chaetomium globosum CBS 148.51
Myceliophthora thermophila ATCC 42464
Verticillium dahliae VdLs.17
Verticillium alfalfae VaMs.102
Colletotrichum graminicola M1.001
Colletotrichum higginsianum
Nectria haematococca mpVI 77-13-4
Fusarium pseudograminearum CS3096
Fusarium graminearum PH-1
Fusarium verticillioides 7600
Fusarium oxysporum f. sp. lycopersici 4287
Fusarium oxysporum Fo5176
Trichoderma atroviride IMI 206040
Trichoderma virens Gv29-8
Trichoderma reesei QM6a
Beauveria bassiana ARSEF 2860
Cordyceps militaris CM01
Metarhizium acridum CQMa 102
Metarhizium robertsii ARSEF 23
Baudoinia compniacensis UAMH 10762
Zymoseptoria tritici IPO323
Passalora fulva CBS 131901
Dothistroma septosporum NZE10
Pseudocercospora fijiensis CIRAD86
Sphaerulina populicola P02.02b
Sphaerulina musiva SO2202
Rhytidhysteron rufulum CBS 306.38
Hysterium pulicare CBS 123377
Phaeosphaeria nodorum SN15
Leptosphaeria maculans JN3
Pyrenophora tritici-repentis Pt-1C-BFP
Pyrenophora teres f. teres 0-1
Setosphaeria turcica Et28A
Bipolaris sorokiniana ND90Pr
Bipolaris maydis ATCC 48331
Bipolaris maydis C5
Exophiala dermatitidis NIH/UT8656
Talaromyces stipitatus ATCC 10500
Talaromyces marneffei ATCC 18224
Penicillium rubens Wisconsin 54-1255
Aspergillus clavatus NRRL 1
Neosartorya fischeri NRRL 181
Aspergillus fumigatus Af293
Aspergillus fumigatus A1163
Aspergillus terreus NIH2624
Aspergillus flavus NRRL3357
Aspergillus oryzae RIB40
Aspergillus nidulans FGSC A4
Aspergillus kawachii IFO 4308
Aspergillus niger ATCC 1015
Aspergillus niger CBS 513.88
Paracoccidioides sp. 'lutzii' Pb01
Paracoccidioides brasiliensis Pb18
Blastomyces dermatitidis SLH14081
Histoplasma capsulatum NAm1
Uncinocarpus reesii 1704
Coccidioides immitis RS
Coccidioides posadasii C735 delta SOWgp
Coccidioides posadasii str. Silveira
Arthroderma otae CBS 113480
Microsporum gypseum CBS 118893
Trichophyton tonsurans CBS 112818
Trichophyton rubrum CBS 118892
Trichophyton verrucosum HKI 0517
Arthroderma benhamiae CBS 112371

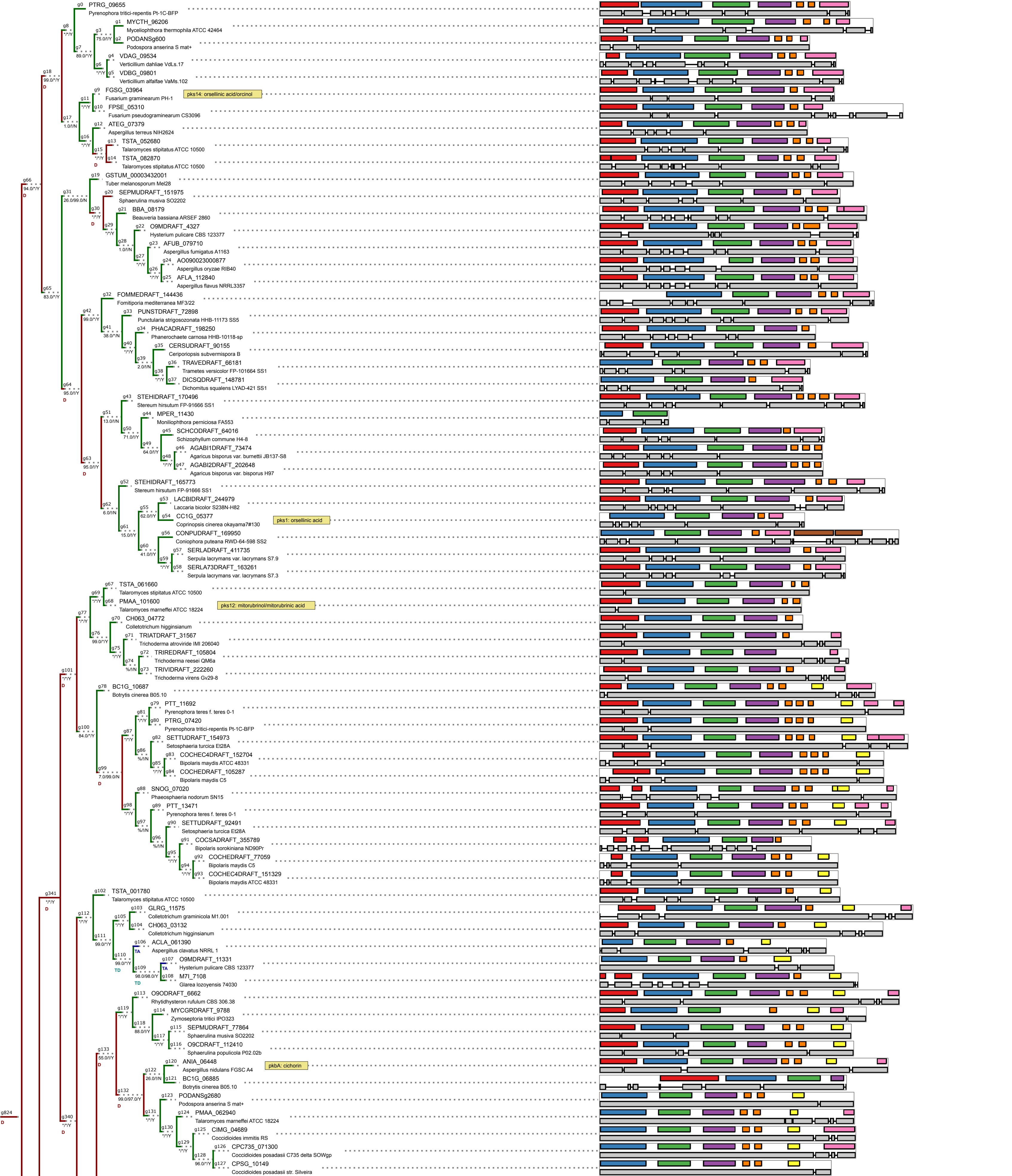

PTRG_09655
Pyrenophora tritici-repentis Pt-1C-BFP
MYCTH_96206
Myceliophthora thermophila ATCC 42464
PODANSg600
Podospora anserina S mat+
VDAG_09534
Verticillium dahliae VdLs.17
VDBG_09801
Verticillium alfalfae VaMs.102
FGSG_03964
Fusarium graminearum PH-1
pks14: orsellinic acid/orcinol
FPSE_05310
Fusarium pseudograminearum CS3096
ATEG_07379
Aspergillus terreus NIH2624
TSTA_052680
Talaromyces stipitatus ATCC 10500
TSTA_082870
Talaromyces stipitatus ATCC 10500
GSTUM_00003432001
Tuber melanosporum Mel28
SEPMUDRAFT_151975
Sphaerulina musiva SO2202
BBA_08179
Beauveria bassiana ARSEF 2860
O9MDRAFT_4327
Hysterium pulicare CBS 123377
AFUB_079710
Aspergillus fumigatus A1163
AO090023000877
Aspergillus oryzae RIB40
AFLA_112840
Aspergillus flavus NRRL3357
FOMMEDRAFT_144436
Fomitiporia mediterranea MF3/22
PUNSTDRAFT_72898
Punctularia strigosozonata HHB-11173 SS5
PHACADRAFT_198250
Phanerochaete carnosa HHB-10118-sp
CERSUDRAFT_90155
Ceriporiopsis subvermispora B
TRAVEDRAFT_66181
Trametes versicolor FP-101664 SS1
DICSQDRAFT_148781
Dichomitus squalens LYAD-421 SS1
STEHIDRAFT_170496
Stereum hirsutum FP-91666 SS1
MPER_11430
Moniliophthora perniciosa FA553
SCHCODRAFT_64016
Schizophyllum commune H4-8
AGABI1DRAFT_73474
Agaricus bisporus var. burnettii JB137-S8
AGABI2DRAFT_202648
Agaricus bisporus var. bisporus H97
STEHIDRAFT_165773
Stereum hirsutum FP-91666 SS1
LACBIDRAFT_244979
Laccaria bicolor S238N-H82
CC1G_05377
Coprinopsis cinerea okayama7#130
pks1: orsellinic acid
CONPUDRAFT_169950
Coniophora puteana RWD-64-598 SS2
SERLADRAFT_411735
Serpula lacrymans var. lacrymans S7.9
SERLA73DRAFT_163261
Serpula lacrymans var. lacrymans S7.3
TSTA_061660
Talaromyces stipitatus ATCC 10500
PMAA_101600
Talaromyces marneffei ATCC 18224
pks12: mitorubrinol/mitorubrinic acid
CH063_04772
Colletotrichum higginsianum
TRIATDRAFT_31567
Trichoderma atroviride IMI 206040
TRIREDRAFT_105804
Trichoderma reesei QM6a
TRIVIDRAFT_222260
Trichoderma virens Gv29-8
BC1G_10687
Botrytis cinerea B05.10
PTT_11692
Pyrenophora teres f. teres 0-1
PTRG_07420
Pyrenophora tritici-repentis Pt-1C-BFP
SETTUDRAFT_154973
Setosphaeria turcica Et28A
COCHEC4DRAFT_152704
Bipolaris maydis ATCC 48331
COCHEDRAFT_105287
Bipolaris maydis C5
SNOG_07020
Phaeosphaeria nodorum SN15
PTT_13471
Pyrenophora teres f. teres 0-1
SETTUDRAFT_92491
Setosphaeria turcica Et28A
COCSADRAFT_355789
Bipolaris sorokiniana ND90Pr
COCHEDRAFT_77059
Bipolaris maydis C5
COCHEC4DRAFT_151329
Bipolaris maydis ATCC 48331
TSTA_001780
Talaromyces stipitatus ATCC 10500
GLRG_11575
Colletotrichum graminicola M1.001
CH063_03132
Colletotrichum higginsianum
ACLA_061390
Aspergillus clavatus NRRL 1
O9MDRAFT_11331
Hysterium pulicare CBS 123377
M7I_7108
Glarea lozoyensis 74030
O9ODRAFT_6662
Rhytidhysteron rufulum CBS 306.38
MYCGRDRAFT_9788
Zymoseptoria tritici IPO323
SEPMUDRAFT_77864
Sphaerulina musiva SO2202
O9CDRAFT_112410
Sphaerulina populicola P02.02b
ANIA_06448
Aspergillus nidulans FGSC A4
pkbA: cichorin
BC1G_06885
Botrytis cinerea B05.10
PODANSg2680
Podospora anserina S mat+
PMAA_062940
Talaromyces marneffei ATCC 18224
CIMG_04689
Coccidioides immitis RS
CPC735_071300
Coccidioides posadasii C735 delta SOWgp
CPSG_10149
Coccidioides posadasii str. Silveira

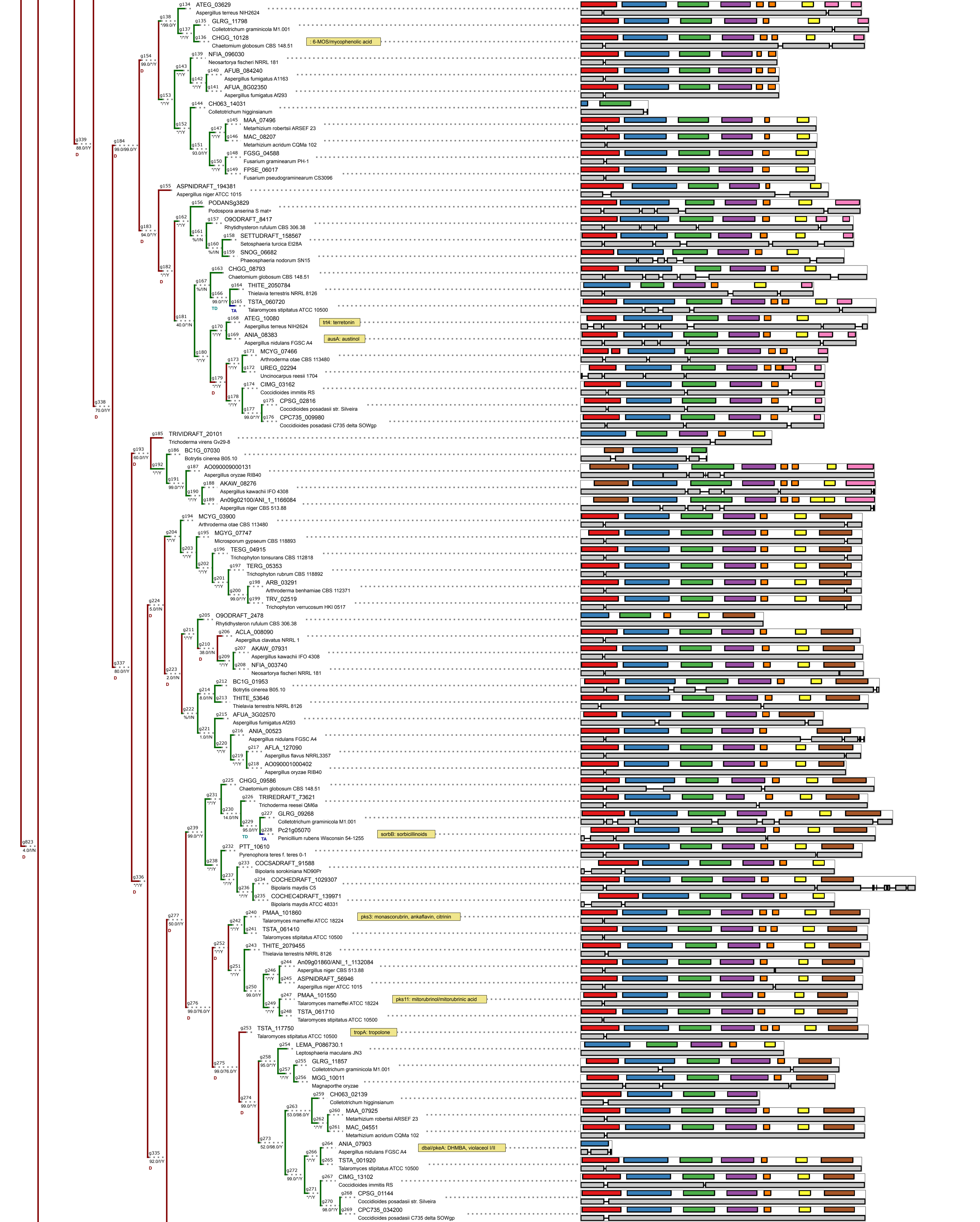

g134 ATEG_03629
Aspergillus terreus NIH2624
g138 */99.0/Y
g135 GLRG_11798
Colletotrichum graminicola M1.001
g137 */*/Y
g136 CHGG_10128
Chaetomium globosum CBS 148.51
6-MOS/mycophenolic acid
g154 99.0/*/Y D
g139 NFIA_096030
Neosartorya fischeri NRRL 181
g143 */*/Y
g140 AFUB_084240
Aspergillus fumigatus A1163
g142 */*/Y
g141 AFUA_8G02350
Aspergillus fumigatus Af293
g153 */*/Y
g144 CH063_14031
Colletotrichum higginsianum
g152 */*/Y
g145 MAA_07496
Metarhizium robertsii ARSEF 23
g147 */*/Y
g146 MAC_08207
Metarhizium acridum CQMa 102
g151 93.0/!/Y
g148 FGSG_04588
Fusarium graminearum PH-1
g150 */*/Y
g149 FPSE_06017
Fusarium pseudograminearum CS3096
g339 88.0/!/Y D
g184 99.0/99.0/Y D
g155 ASPNIDRAFT_194381
Aspergillus niger ATCC 1015
g156 PODANSg3829
Podospora anserina S mat+
g162 */*/Y
g157 O9ODRAFT_8417
Rhytidhysteron rufulum CBS 306.38
g161 %/!/N
g158 SETTUDRAFT_158567
Setosphaeria turcica Et28A
g160 %/!/N
g159 SNOG_06682
Phaeosphaeria nodorum SN15
g183 94.0/*/Y D
g182 */*/Y D
g163 CHGG_08793
Chaetomium globosum CBS 148.51
g167 %/!/N
g164 THITE_2050784
Thielavia terrestris NRRL 8126
g166 99.0/*/Y TD
g165 TA TSTA_060720
Talaromyces stipitatus ATCC 10500
g181 40.0/*/N
g168 ATEG_10080
Aspergillus terreus NIH2624
trt4: terretonin
g170 */*/Y
g169 ANIA_08383
Aspergillus nidulans FGSC A4
ausA: austinol
g180 */*/Y
g171 MCYG_07466
Arthroderma otae CBS 113480
g173 */*/Y
g172 UREG_02294
Uncinocarpus reesii 1704
g179 */*/Y D
g174 CIMG_03162
Coccidioides immitis RS
g178 */*/Y
g175 CPSG_02816
Coccidioides posadasii str. Silveira
g177 99.0/*/Y
g176 CPC735_009980
Coccidioides posadasii C735 delta SOWgp
g338 70.0/!/Y D
g185 TRIVIDRAFT_20101
Trichoderma virens Gv29-8
g193 60.0/!/Y D
g186 BC1G_07030
Botrytis cinerea B05.10
g192 */*/Y
g187 AO090009000131
Aspergillus oryzae RIB40
g191 99.0/*/Y
g188 AKAW_08276
Aspergillus kawachii IFO 4308
g190 */*/Y
g189 An09g02100/ANI_1_1166084
Aspergillus niger CBS 513.88
g194 MCYG_03900
Arthroderma otae CBS 113480
g204 */*/Y
g195 MGYG_07747
Microsporum gypseum CBS 118893
g203 */*/Y
g196 TESG_04915
Trichophyton tonsurans CBS 112818
g202 */*/Y
g197 TERG_05353
Trichophyton rubrum CBS 118892
g201 */*/Y
g198 ARB_03291
Arthroderma benhamiae CBS 112371
g200 99.0/*/Y
g199 TRV_02519
Trichophyton verrucosum HKI 0517
g224 5.0/!/N D
g205 O9ODRAFT_2478
Rhytidhysteron rufulum CBS 306.38
g211 */*/Y
g206 ACLA_008090
Aspergillus clavatus NRRL 1
g210 38.0/!/N D
g207 AKAW_07931
Aspergillus kawachii IFO 4308
g209 */*/Y
g208 NFIA_003740
Neosartorya fischeri NRRL 181
g337 80.0/!/Y D
g223 2.0/!/N D
g212 BC1G_01953
Botrytis cinerea B05.10
g214 8.0/!/N
g213 THITE_53646
Thielavia terrestris NRRL 8126
g222 %/!/N
g215 AFUA_3G02570
Aspergillus fumigatus Af293
g221 1.0/!/N
g216 ANIA_00523
Aspergillus nidulans FGSC A4
g220 */*/Y
g217 AFLA_127090
Aspergillus flavus NRRL3357
g219 */*/Y
g218 AO090001000402
Aspergillus oryzae RIB40
g225 CHGG_09586
Chaetomium globosum CBS 148.51
g231 */*/Y
g226 TRIREDRAFT_73621
Trichoderma reesei QM6a
g230 14.0/!/N
g227 GLRG_09268
Colletotrichum graminicola M1.001
g229 95.0/*/Y TD
g228 TA Pc21g05070
Penicillium rubens Wisconsin 54-1255
sorbB: sorbicillinoids
g239 99.0/*/Y
g823 4.0/!/N D
g232 PTT_10610
Pyrenophora teres f. teres 0-1
g238 */*/Y
g233 COCSADRAFT_91588
Bipolaris sorokiniana ND90Pr
g237 */*/Y
g234 COCHEDRAFT_1029307
Bipolaris maydis C5
g236 */*/Y
g235 COCHEC4DRAFT_139971
Bipolaris maydis ATCC 48331
g336 */*/Y D
g240 PMAA_101860
Talaromyces marneffei ATCC 18224
pks3: monascorubrin, ankaflavin, citrinin
g242 */*/Y
g241 TSTA_061410
Talaromyces stipitatus ATCC 10500
g277 50.0/!/Y D
g252 */*/Y D
g243 THITE_2079455
Thielavia terrestris NRRL 8126
g251 */*/Y
g244 An09g01860/ANI_1_1132084
Aspergillus niger CBS 513.88
g246 */*/Y
g245 ASPNIDRAFT_56946
Aspergillus niger ATCC 1015
g250 99.0/!/Y
g247 PMAA_101550
Talaromyces marneffei ATCC 18224
pks11: mitorubrinol/mitorubrinic acid
g249 */*/Y
g248 TSTA_061710
Talaromyces stipitatus ATCC 10500
g276 99.0/76.0/Y D
g253 TSTA_117750
Talaromyces stipitatus ATCC 10500
tropA: tropolone
g254 LEMA_P086730.1
Leptosphaeria maculans JN3
g258 95.0/*/Y
g255 GLRG_11857
Colletotrichum graminicola M1.001
g257 */*/Y
g256 MGG_10011
Magnaporthe oryzae
g275 99.0/76.0/Y D
g274 99.0/*/Y D
g259 CH063_02139
Colletotrichum higginsianum
g263 53.0/98.0/Y
g260 MAA_07925
Metarhizium robertsii ARSEF 23
g262 */*/Y
g261 MAC_04551
Metarhizium acridum CQMa 102
g273 52.0/98.0/Y
g264 ANIA_07903
Aspergillus nidulans FGSC A4
dbaI/pkeA: DHMBA, violaceol I/II
g266 */*/Y
g265 TSTA_001920
Talaromyces stipitatus ATCC 10500
g335 92.0/!/Y D
g272 99.0/*/Y
g267 CIMG_13102
Coccidioides immitis RS
g271 */*/Y
g268 CPSG_01144
Coccidioides posadasii str. Silveira
g270 98.0/*/Y
g269 CPC735_034200
Coccidioides posadasii C735 delta SOWgp

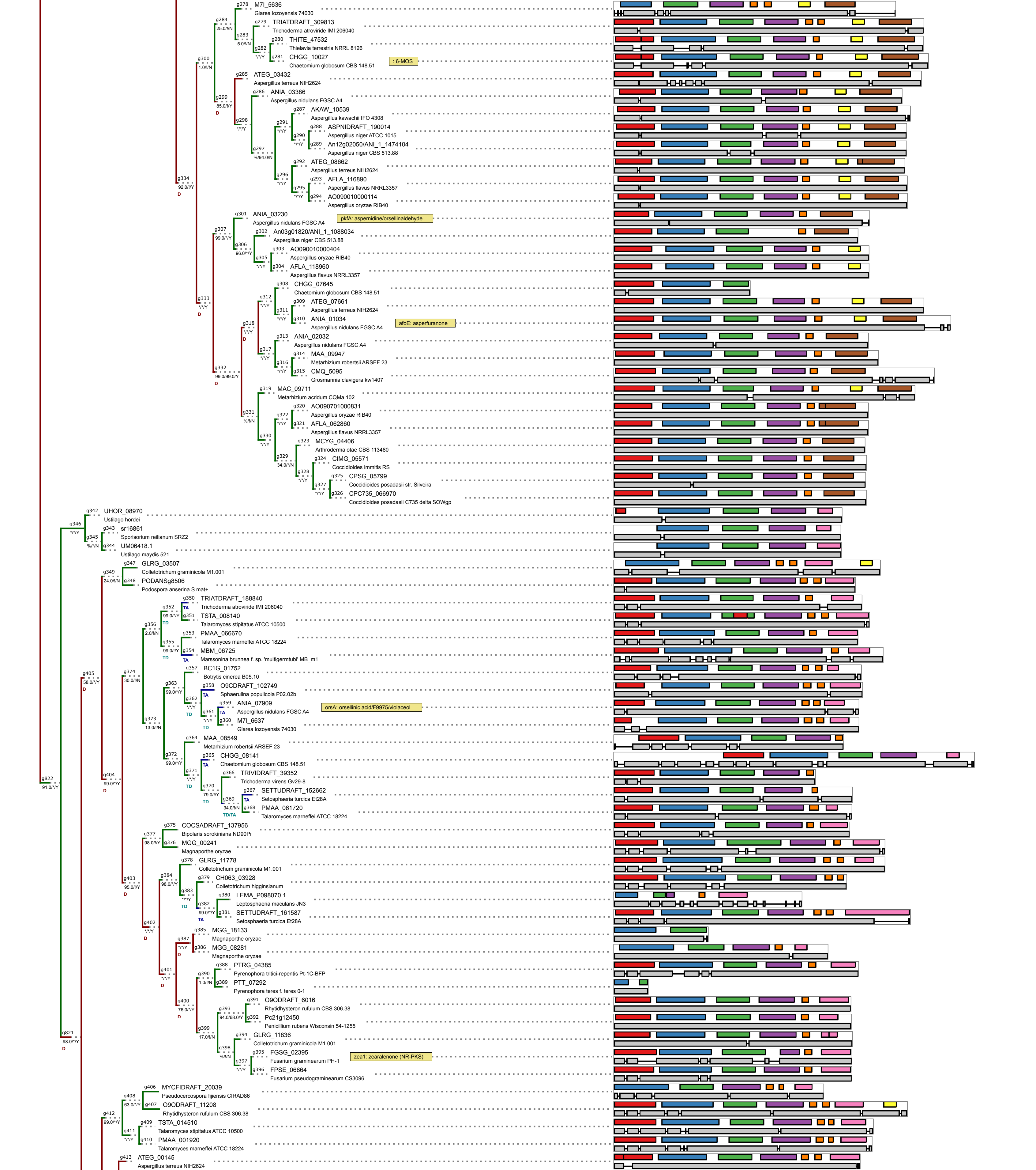

M7I_5636
Glarea lozoyensis 74030
TRIATDRAFT_309813
Trichoderma atroviride IMI 206040
THITE_47532
Thielavia terrestris NRRL 8126
CHGG_10027
Chaetomium globosum CBS 148.51
: 6-MOS
ATEG_03432
Aspergillus terreus NIH2624
ANIA_03386
Aspergillus nidulans FGSC A4
AKAW_10539
Aspergillus kawachii IFO 4308
ASPNIDRAFT_190014
Aspergillus niger ATCC 1015
An12g02050/ANI_1_1474104
Aspergillus niger CBS 513.88
ATEG_08662
Aspergillus terreus NIH2624
AFLA_116890
Aspergillus flavus NRRL3357
AO090010000114
Aspergillus oryzae RIB40
ANIA_03230
Aspergillus nidulans FGSC A4
pkfA: aspernidine/orsellinaldehyde
An03g01820/ANI_1_1088034
Aspergillus niger CBS 513.88
AO090010000404
Aspergillus oryzae RIB40
AFLA_118960
Aspergillus flavus NRRL3357
CHGG_07645
Chaetomium globosum CBS 148.51
ATEG_07661
Aspergillus terreus NIH2624
ANIA_01034
Aspergillus nidulans FGSC A4
afoE: asperfuranone
ANIA_02032
Aspergillus nidulans FGSC A4
MAA_09947
Metarhizium robertsii ARSEF 23
CMQ_5095
Grosmannia clavigera kw1407
MAC_09711
Metarhizium acridum CQMa 102
AO090701000831
Aspergillus oryzae RIB40
AFLA_062860
Aspergillus flavus NRRL3357
MCYG_04406
Arthroderma otae CBS 113480
CIMG_05571
Coccidioides immitis RS
CPSG_05799
Coccidioides posadasii str. Silveira
CPC735_066970
Coccidioides posadasii C735 delta SOWgp
UHOR_08970
Ustilago hordei
sr16861
Sporisorium reilianum SRZ2
UM06418.1
Ustilago maydis 521
GLRG_03507
Colletotrichum graminicola M1.001
PODANSg8506
Podospora anserina S mat+
TRIATDRAFT_188840
Trichoderma atroviride IMI 206040
TSTA_008140
Talaromyces stipitatus ATCC 10500
PMAA_066670
Talaromyces marneffei ATCC 18224
MBM_06725
Marssonina brunnea f. sp. 'multigermtubi' MB_m1
BC1G_01752
Botrytis cinerea B05.10
O9CDRAFT_102749
Sphaerulina populicola P02.02b
ANIA_07909
Aspergillus nidulans FGSC A4
orsA: orsellinic acid/F9975/violaceol
M7I_6637
Glarea lozoyensis 74030
MAA_08549
Metarhizium robertsii ARSEF 23
CHGG_08141
Chaetomium globosum CBS 148.51
TRIVIDRAFT_39352
Trichoderma virens Gv29-8
SETTUDRAFT_152662
Setosphaeria turcica Et28A
PMAA_061720
Talaromyces marneffei ATCC 18224
COCSADRAFT_137956
Bipolaris sorokiniana ND90Pr
MGG_00241
Magnaporthe oryzae
GLRG_11778
Colletotrichum graminicola M1.001
CH063_03928
Colletotrichum higginsianum
LEMA_P098070.1
Leptosphaeria maculans JN3
SETTUDRAFT_161578
Setosphaeria turcica Et28A
MGG_18133
Magnaporthe oryzae
MGG_08281
Magnaporthe oryzae
PTRG_04385
Pyrenophora tritici-repentis Pt-1C-BFP
PTT_07292
Pyrenophora teres f. teres 0-1
O9ODRAFT_6016
Rhytidhysteron rufulum CBS 306.38
Pc21g12450
Penicillium rubens Wisconsin 54-1255
GLRG_11836
Colletotrichum graminicola M1.001
FGSG_02395
Fusarium graminearum PH-1
zea1: zearalenone (NR-PKS)
FPSE_06864
Fusarium pseudograminearum CS3096
MYCFIDRAFT_20039
Pseudocercospora fijiensis CIRAD86
O9ODRAFT_11208
Rhytidhysteron rufulum CBS 306.38
TSTA_014510
Talaromyces stipitatus ATCC 10500
PMAA_001920
Talaromyces marneffei ATCC 18224
ATEG_00145
Aspergillus terreus NIH2624

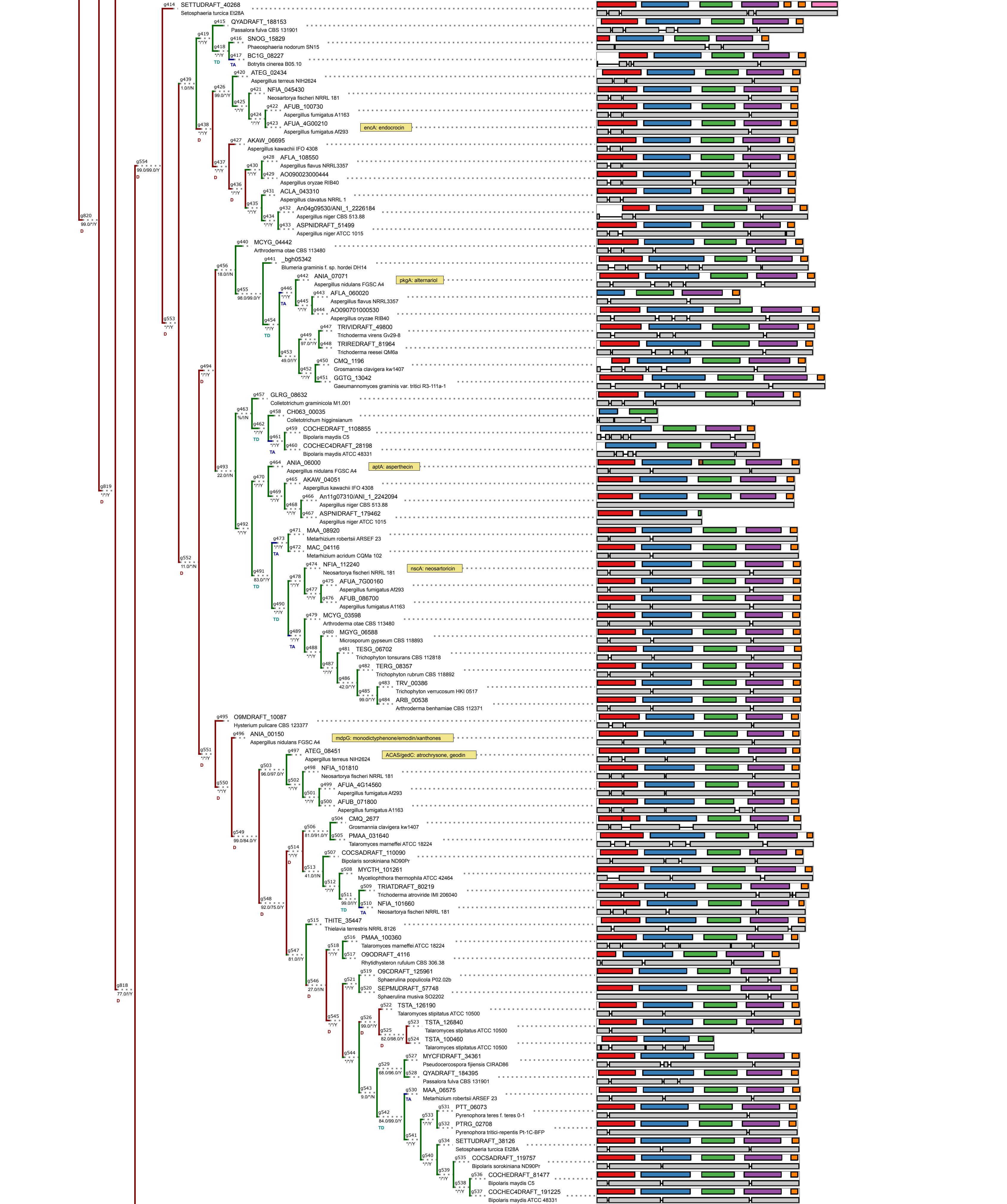
SETTUDRAFT_40268
Setosphaeria turcica Et28A
QYADRAFT_188153
Passalora fulva CBS 131901
SNOG_15829
Phaeosphaeria nodorum SN15
BC1G_08227
Botrytis cinerea B05.10
ATEG_02434
Aspergillus terreus NIH2624
NFIA_045430
Neosartorya fischeri NRRL 181
AFUB_100730
Aspergillus fumigatus A1163
AFUA_4G00210
Aspergillus fumigatus Af293
encA: endocrocin
AKAW_06695
Aspergillus kawachii IFO 4308
AFLA_108550
Aspergillus flavus NRRL3357
AO090023000444
Aspergillus oryzae RIB40
ACLA_043310
Aspergillus clavatus NRRL 1
An04g09530/ANI_1_2226184
Aspergillus niger CBS 513.88
ASPNIDRAFT_51499
Aspergillus niger ATCC 1015
MCYG_04442
Arthroderma otae CBS 113480
_bgh05342
Blumeria graminis f. sp. hordei DH14
ANIA_07071
Aspergillus nidulans FGSC A4
pkgA: alternariol
AFLA_060020
Aspergillus flavus NRRL3357
AO090701000530
Aspergillus oryzae RIB40
TRIVIDRAFT_49800
Trichoderma virens Gv29-8
TRIREDRAFT_81964
Trichoderma reesei QM6a
CMQ_1196
Grosmannia clavigera kw1407
GGTG_13042
Gaeumannomyces graminis var. tritici R3-111a-1
GLRG_08632
Colletotrichum graminicola M1.001
CH063_00035
Colletotrichum higginsianum
COCHEDRAFT_1108855
Bipolaris maydis C5
COCHEC4DRAFT_28198
Bipolaris maydis ATCC 48331
ANIA_06000
Aspergillus nidulans FGSC A4
aptA: asperthecin
AKAW_04051
Aspergillus kawachii IFO 4308
An11g07310/ANI_1_2242094
Aspergillus niger CBS 513.88
ASPNIDRAFT_179462
Aspergillus niger ATCC 1015
MAA_08920
Metarhizium robertsii ARSEF 23
MAC_04116
Metarhizium acridum CQMa 102
NFIA_112240
Neosartorya fischeri NRRL 181
nscA: neosartoricin
AFUA_7G00160
Aspergillus fumigatus Af293
AFUB_086700
Aspergillus fumigatus A1163
MCYG_03598
Arthroderma otae CBS 113480
MGYG_06588
Microsporum gypseum CBS 118893
TESG_06702
Trichophyton tonsurans CBS 112818
TERG_08357
Trichophyton rubrum CBS 118892
TRV_00386
Trichophyton verrucosum HKI 0517
ARB_00538
Arthroderma benhamiae CBS 112371
O9MDRAFT_10087
Hysterium pulicare CBS 123377
ANIA_00150
Aspergillus nidulans FGSC A4
mdpG: monodictyphenone/emodin/xanthones
ATEG_08451
Aspergillus terreus NIH2624
ACAS/gedC: atrochrysone, geodin
NFIA_101810
Neosartorya fischeri NRRL 181
AFUA_4G14560
Aspergillus fumigatus Af293
AFUB_071800
Aspergillus fumigatus A1163
CMQ_2677
Grosmannia clavigera kw1407
PMAA_031640
Talaromyces marneffei ATCC 18224
COCSADRAFT_110090
Bipolaris sorokiniana ND90Pr
MYCTH_101261
Myceliophthora thermophila ATCC 42464
TRIATDRAFT_80219
Trichoderma atroviride IMI 206040
NFIA_101660
Neosartorya fischeri NRRL 181
THITE_35447
Thielavia terrestris NRRL 8126
PMAA_100360
Talaromyces marneffei ATCC 18224
O9ODRAFT_4116
Rhytidhysteron rufulum CBS 306.38
O9CDRAFT_125961
Sphaerulina populicola P02.02b
SEPMUDRAFT_57748
Sphaerulina musiva SO2202
TSTA_126190
Talaromyces stipitatus ATCC 10500
TSTA_126840
Talaromyces stipitatus ATCC 10500
TSTA_100460
Talaromyces stipitatus ATCC 10500
MYCFIDRAFT_34361
Pseudocercospora fijiensis CIRAD86
QYADRAFT_184395
Passalora fulva CBS 131901
MAA_06575
Metarhizium robertsii ARSEF 23
PTT_06073
Pyrenophora teres f. teres 0-1
PTRG_02708
Pyrenophora tritici-repentis Pt-1C-BFP
SETTUDRAFT_38126
Setosphaeria turcica Et28A
COCSADRAFT_119757
Bipolaris sorokiniana ND90Pr
COCHEDRAFT_81477
Bipolaris maydis C5
COCHEC4DRAFT_191225
Bipolaris maydis ATCC 48331

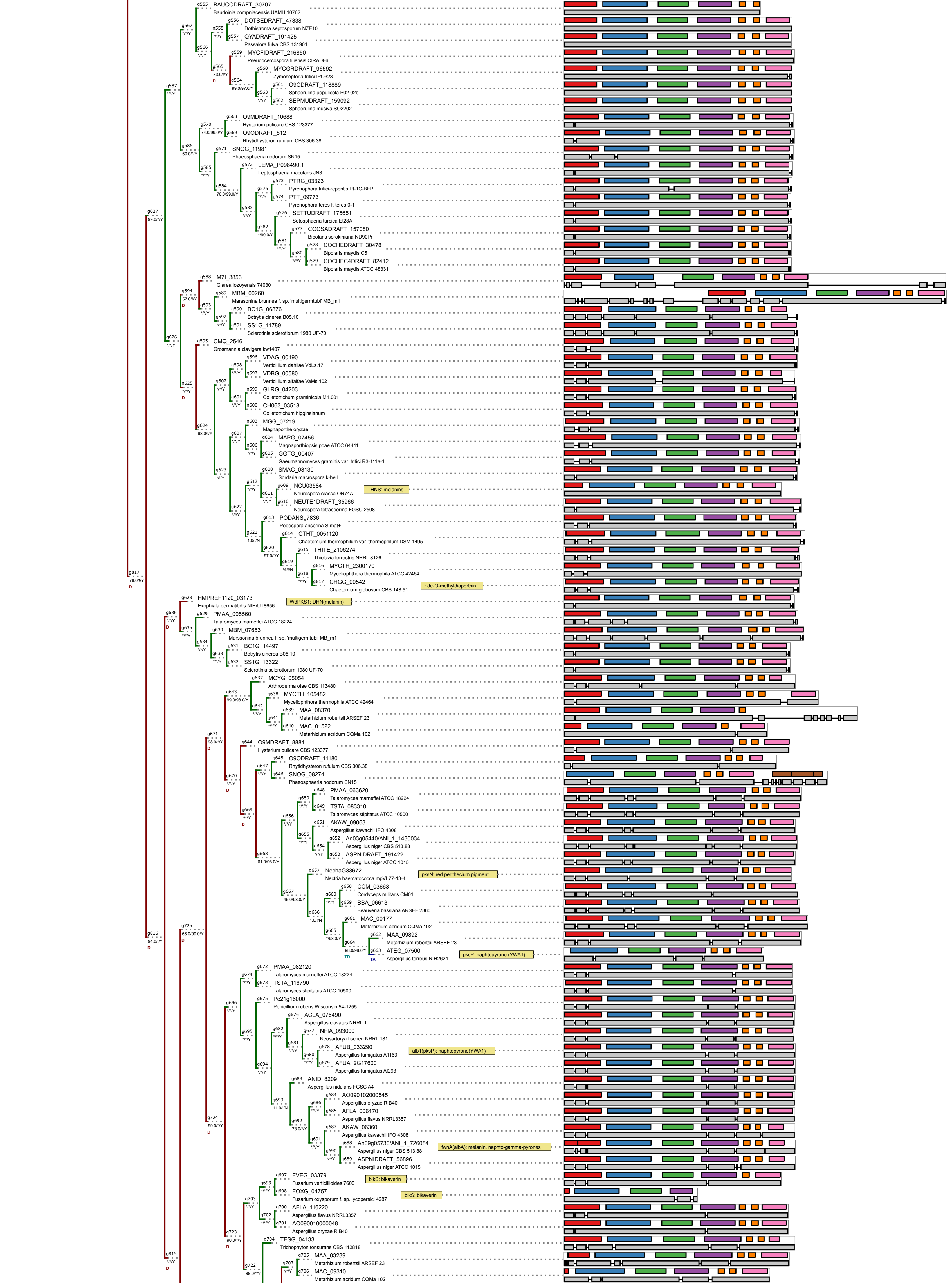

BAUCODRAFT_30707
Baudoinia compniacensis UAMH 10762
DOTSEDRAFT_47338
Dothistroma septosporum NZE10
QYADRAFT_191425
Passalora fulva CBS 131901
MYCFIDRAFT_216850
Pseudocercospora fijiensis CIRAD86
MYCGRDRAFT_96592
Zymoseptoria tritici IPO323
O9CDRAFT_118889
Sphaerulina populicola P02.02b
SEPMUDRAFT_159092
Sphaerulina musiva SO2202
O9MDRAFT_10688
Hysterium pulicare CBS 123377
O9ODRAFT_812
Rhytidhysteron rufulum CBS 306.38
SNOG_11981
Phaeosphaeria nodorum SN15
LEMA_P098490.1
Leptosphaeria maculans JN3
PTRG_03323
Pyrenophora tritici-repentis Pt-1C-BFP
PTT_09773
Pyrenophora teres f. teres 0-1
SETTUDRAFT_175651
Setosphaeria turcica Et28A
COCSADRAFT_157080
Bipolaris sorokiniana ND90Pr
COCHEDRAFT_30478
Bipolaris maydis C5
COCHEC4DRAFT_82412
Bipolaris maydis ATCC 48331
M7I_3853
Glarea lozoyensis 74030
MBM_00260
Marssonina brunnea f. sp. 'multigermtubi' MB_m1
BC1G_06876
Botrytis cinerea B05.10
SS1G_11789
Sclerotinia sclerotiorum 1980 UF-70
CMQ_2546
Grosmannia clavigera kw1407
VDAG_00190
Verticillium dahliae VdLs.17
VDBG_00580
Verticillium alfalfae VaMs.102
GLRG_04203
Colletotrichum graminicola M1.001
CH063_03518
Colletotrichum higginsianum
MGG_07219
Magnaporthe oryzae
MAPG_07456
Magnaporthiopsis poae ATCC 64411
GGTG_00407
Gaeumannomyces graminis var. tritici R3-111a-1
SMAC_03130
Sordaria macrospora k-hell
NCU03584
Neurospora crassa OR74A
THNS: melanins
NEUTE1DRAFT_35966
Neurospora tetrasperma FGSC 2508
PODANSg7836
Podospora anserina S mat+
CTHT_0051120
Chaetomium thermophilum var. thermophilum DSM 1495
THITE_2106274
Thielavia terrestris NRRL 8126
MYCTH_2300170
Myceliophthora thermophila ATCC 42464
CHGG_00542
Chaetomium globosum CBS 148.51
: de-O-methyldiaporthin
HMPREF1120_03173
Exophiala dermatitidis NIH/UT8656
WdPKS1: DHN(melanin)
PMAA_095560
Talaromyces marneffei ATCC 18224
MBM_07653
Marssonina brunnea f. sp. 'multigermtubi' MB_m1
BC1G_14497
Botrytis cinerea B05.10
SS1G_13322
Sclerotinia sclerotiorum 1980 UF-70
MCYG_05054
Arthroderma otae CBS 113480
MYCTH_105482
Myceliophthora thermophila ATCC 42464
MAA_08370
Metarhizium robertsii ARSEF 23
MAC_01522
Metarhizium acridum CQMa 102
O9MDRAFT_8884
Hysterium pulicare CBS 123377
O9ODRAFT_11180
Rhytidhysteron rufulum CBS 306.38
SNOG_08274
Phaeosphaeria nodorum SN15
PMAA_063620
Talaromyces marneffei ATCC 18224
TSTA_083310
Talaromyces stipitatus ATCC 10500
AKAW_09063
Aspergillus kawachii IFO 4308
An03g05440/ANI_1_1430034
Aspergillus niger CBS 513.88
ASPNIDRAFT_191422
Aspergillus niger ATCC 1015
NechaG33672
Nectria haematococca mpVI 77-13-4
pksN: red perithecium pigment
CCM_03663
Cordyceps militaris CM01
BBA_06613
Beauveria bassiana ARSEF 2860
MAC_00177
Metarhizium acridum CQMa 102
MAA_09892
Metarhizium robertsii ARSEF 23
ATEG_07500
Aspergillus terreus NIH2624
pksP: naphtopyrone (YWA1)
PMAA_082120
Talaromyces marneffei ATCC 18224
TSTA_116790
Talaromyces stipitatus ATCC 10500
Pc21g16000
Penicillium rubens Wisconsin 54-1255
ACLA_076490
Aspergillus clavatus NRRL 1
NFIA_093000
Neosartorya fischeri NRRL 181
AFUB_033290
Aspergillus fumigatus A1163
alb1(pksP): naphtopyrone(YWA1)
AFUA_2G17600
Aspergillus fumigatus Af293
ANID_8209
Aspergillus nidulans FGSC A4
AO090102000545
Aspergillus oryzae RIB40
AFLA_006170
Aspergillus flavus NRRL3357
AKAW_06360
Aspergillus kawachii IFO 4308
An09g05730/ANI_1_726084
Aspergillus niger CBS 513.88
fwnA(albA): melanin, naphto-gamma-pyrones
ASPNIDRAFT_56896
Aspergillus niger ATCC 1015
FVEG_03379
Fusarium verticillioides 7600
bikS: bikaverin
FOXG_04757
Fusarium oxysporum f. sp. lycopersici 4287
bikS: bikaverin
AFLA_116220
Aspergillus flavus NRRL3357
AO090010000048
Aspergillus oryzae RIB40
TESG_04133
Trichophyton tonsurans CBS 112818
MAA_03239
Metarhizium robertsii ARSEF 23
MAC_09310
Metarhizium acridum CQMa 102

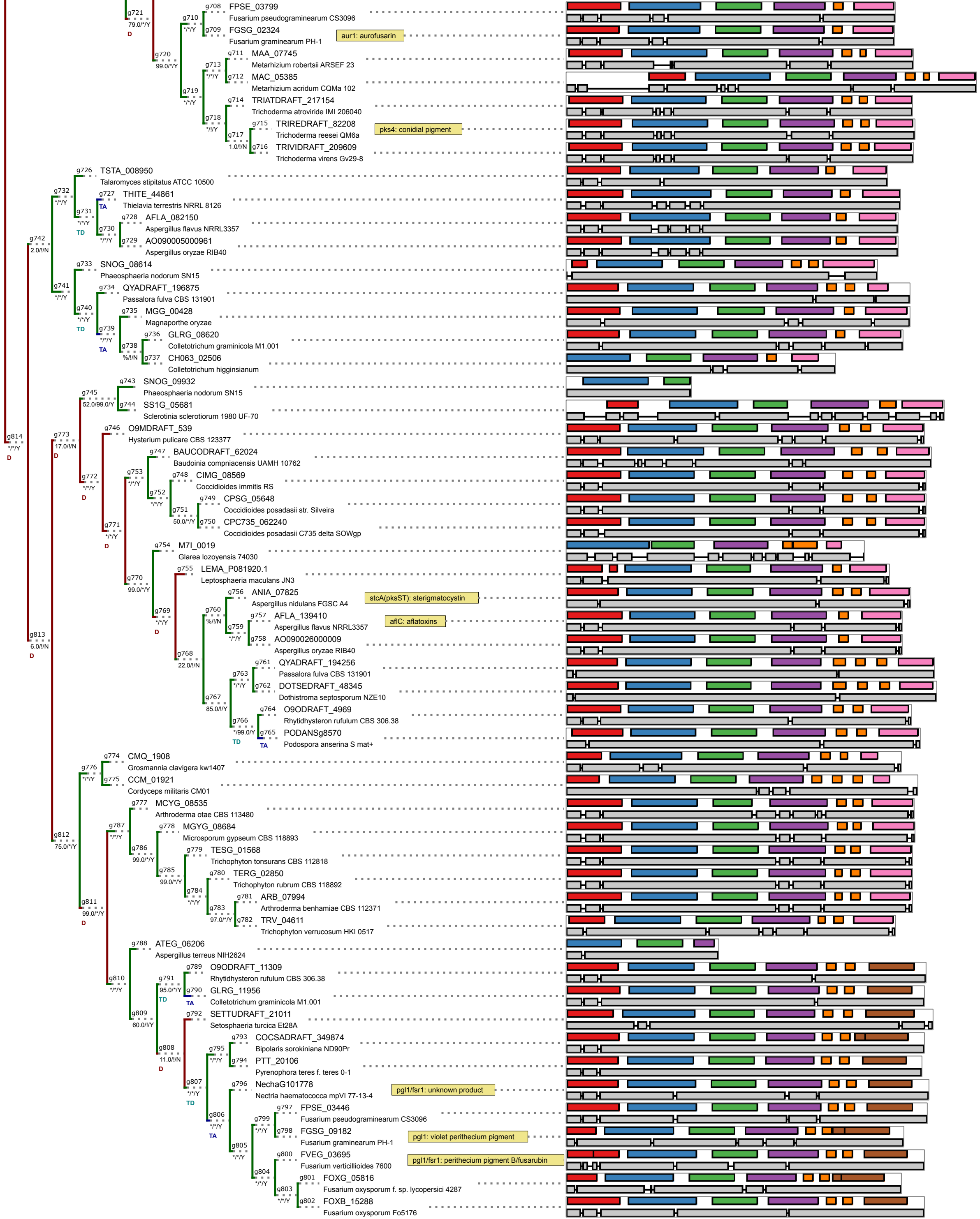

g721 79.0/*/Y D
g708 FPSE_03799
Fusarium pseudograminearum CS3096
g710 */*/Y
g709 FGSG_02324
Fusarium graminearum PH-1
aur1: aurofusarin
g720 99.0/*/Y
g711 MAA_07745
Metarhizium robertsii ARSEF 23
g713 */*/Y
g712 MAC_05385
Metarhizium acridum CQMa 102
g719 */*/Y
g714 TRIATDRAFT_217154
Trichoderma atroviride IMI 206040
g718 */!/Y
g715 TRIREDRAFT_82208
Trichoderma reesei QM6a
pks4: conidial pigment
g717 1.0/!/N
g716 TRIVIDRAFT_209609
Trichoderma virens Gv29-8
g726 TSTA_008950
Talaromyces stipitatus ATCC 10500
g732 */*/Y
g727 THITE_44861 TA
Thielavia terrestris NRRL 8126
g731 */*/Y TD
g728 AFLA_082150
Aspergillus flavus NRRL3357
g730 */*/Y
g729 AO090005000961
Aspergillus oryzae RIB40
g742 2.0/!/N
g733 SNOG_08614
Phaeosphaeria nodorum SN15
g741 */*/Y
g734 QYADRAFT_196875
Passalora fulva CBS 131901
g740 */*/Y TD
g735 MGG_00428
Magnaporthe oryzae
g739 */*/Y TA
g736 GLRG_08620
Colletotrichum graminicola M1.001
g738 %/!/N
g737 CH063_02506
Colletotrichum higginsianum
g743 SNOG_09932
Phaeosphaeria nodorum SN15
g745 52.0/99.0/Y
g744 SS1G_05681
Sclerotinia sclerotiorum 1980 UF-70
g814 */*/Y D
g773 17.0/!/N D
g746 O9MDRAFT_539
Hysterium pulicare CBS 123377
g747 BAUCODRAFT_62024
Baudoinia compniacensis UAMH 10762
g753 */*/Y
g748 CIMG_08569
Coccidioides immitis RS
g772 */*/Y D
g752 */*/Y
g749 CPSG_05648
Coccidioides posadasii str. Silveira
g751 50.0/*/Y
g750 CPC735_062240
Coccidioides posadasii C735 delta SOWgp
g771 */*/Y D
g754 M7I_0019
Glarea lozoyensis 74030
g755 LEMA_P081920.1
Leptosphaeria maculans JN3
g770 99.0/*/Y
g769 */*/Y D
g756 ANIA_07825
Aspergillus nidulans FGSC A4
stcA(pksST): sterigmatocystin
g760 %/!/N
g757 AFLA_139410
Aspergillus flavus NRRL3357
aflC: aflatoxins
g759 */*/Y
g758 AO090026000009
Aspergillus oryzae RIB40
g813 6.0/!/N D
g768 22.0/!/N
g761 QYADRAFT_194256
Passalora fulva CBS 131901
g763 */*/Y
g762 DOTSEDRAFT_48345
Dothistroma septosporum NZE10
g767 85.0/!/Y
g764 O9ODRAFT_4969
Rhytidhysteron rufulum CBS 306.38
g766 */99.0/Y TD
g765 PODANSg8570 TA
Podospora anserina S mat+
g774 CMQ_1908
Grosmannia clavigera kw1407
g776 */*/Y
g775 CCM_01921
Cordyceps militaris CM01
g777 MCYG_08535
Arthroderma otae CBS 113480
g787 */*/Y
g778 MGYG_08684
Microsporum gypseum CBS 118893
g786 99.0/*/Y
g779 TESG_01568
Trichophyton tonsurans CBS 112818
g812 75.0/*/Y
g785 99.0/*/Y
g780 TERG_02850
Trichophyton rubrum CBS 118892
g784 */*/Y
g781 ARB_07994
Arthroderma benhamiae CBS 112371
g811 99.0/*/Y D
g783 97.0/*/Y
g782 TRV_04611
Trichophyton verrucosum HKI 0517
g788 ATEG_06206
Aspergillus terreus NIH2624
g789 O9ODRAFT_11309
Rhytidhysteron rufulum CBS 306.38
g810 */*/Y
g791 95.0/*/Y TD
g790 GLRG_11956 TA
Colletotrichum graminicola M1.001
g792 SETTUDRAFT_21011
Setosphaeria turcica Et28A
g809 60.0/!/Y
g793 COCSADRAFT_349874
Bipolaris sorokiniana ND90Pr
g808 11.0/!/N D
g795 */*/Y
g794 PTT_20106
Pyrenophora teres f. teres 0-1
g796 NechaG101778
Nectria haematococca mpVI 77-13-4
pgl1/fsr1: unknown product
g807 */*/Y TD
g797 FPSE_03446
Fusarium pseudograminearum CS3096
g806 */*/Y TA
g799 */*/Y
g798 FGSG_09182
Fusarium graminearum PH-1
pgl1: violet perithecium pigment
g805 */*/Y
g800 FVEG_03695
Fusarium verticillioides 7600
pgl1/fsr1: perithecium pigment B/fusarubin
g804 */*/Y
g801 FOXG_05816
Fusarium oxysporum f. sp. lycopersici 4287
g803 */*/Y
g802 FOXB_15288
Fusarium oxysporum Fo5176

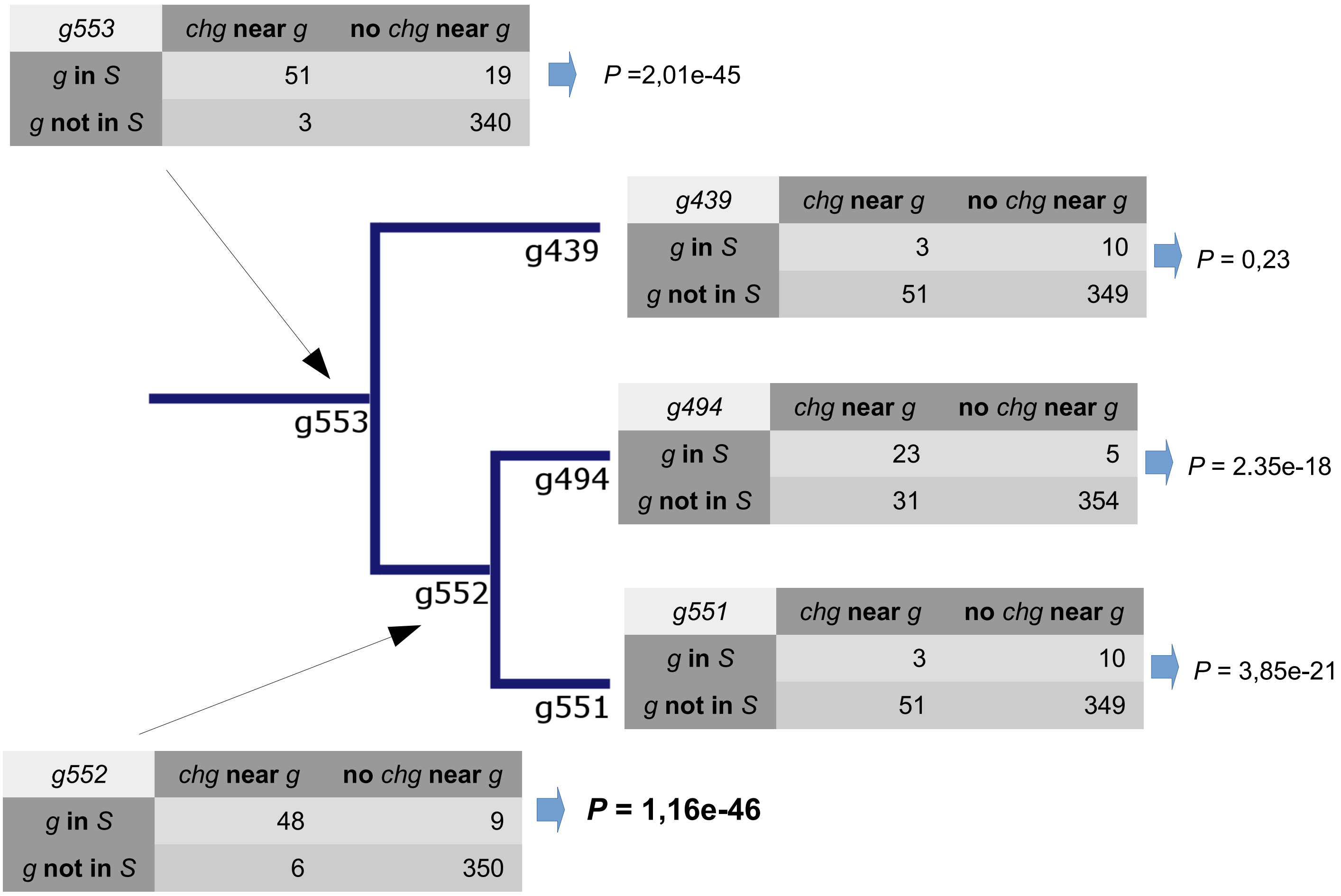

Supplementary Figure SF4. Example of statistical testing conducted to detect strongest, distinct relationships between monophyletic clades and presence/absence of candidate homolog. In the example, β-lactamases (key accessory enzyme for C6-C11 cyclisation) show strongest association with subtree (node) g552, where P-value (pre-Bonferroni correction; one-sided F0isher exact test) hits a distinct minimum. All such non-overlapping minima, scoring above 0.0001 post-correction were further analysed (see Table 2). Notation used: *g* – gene (core NR-PKS), *S* – subtree, *chg* – candidate homolog group member gene.
